# Supplementary material for: IMC-Denoise: a content aware denoising pipeline to enhance Imaging Mass Cytometry
Source: Nat Commun. 2023 Mar 23;14:1601. doi: 10.1038/s41467-023-37123-6 (PMC10036333; doi:10.1038/s41467-023-37123-6)
Supplement: Supplementary file 1 — Supplementary Information [file 41467_2023_37123_MOESM1_ESM.pdf]

# Contents


**Supplementary Note 1: IMC-Denoise framework** **3**

1.1 Imaging mass cytometry (IMC) imaging model . . . . . . . . . . . . . . . . . . . . . . . . . . 3

1.2 Rationale for IMC denoising . . . . . . . . . . . . . . . . . . . . . . . . . . . . . . . . 4

1.2.1 Impact on pixel scale . . . . . . . . . . . . . . . . . . . . . . . . . . . . . . 4

1.2.2 Impact on cell scale . . . . . . . . . . . . . . . . . . . . . . . . . . . . . . . 4

1.3 Differential intensity map-based restoration algorithm for hot pixel removal (DIMR) . . . 5

1.3.1 Hot pixel unmixing . . . . . . . . . . . . . . . . . . . . . . . . . . . . . . . 5

1.3.2 Hot pixel detection . . . . . . . . . . . . . . . . . . . . . . . . . . . . . . . 7

1.4 Self-supervised deep learning-based algorithm for shot noise image filtering (DeepSNiF) . 9

1.4.1 Optimal loss function derivation . . . . . . . . . . . . . . . . . . . . . . . . 9

1.4.2 Hessian norm regularization as a booster for the denoising task . . . . . . . . . . . 12

1.4.3 Image normalization . . . . . . . . . . . . . . . . . . . . . . . . . . . . . . . 13

**Supplementary Note 2: Reference methods** **16**

2.1 Hot pixel removal methods . . . . . . . . . . . . . . . . . . . . . . . . . . . . . . . . 16

2.2 Deep learning-based shot noise filtering methods . . . . . . . . . . . . . . . . . . . . . 17

2.2.1 Noise2Void . . . . . . . . . . . . . . . . . . . . . . . . . . . . . . . . . . . 17

2.2.2 Modified Noise2Void with the Anscombe transformation and rectified linear unit (ReLU) activation . . . . . . . . . . . . . . . . . . . . . . . . . . . . . . . . 18

2.2.3 Noise2True . . . . . . . . . . . . . . . . . . . . . . . . . . . . . . . . . . . 18

2.3 Traditional statistics-based shot noise filtering methods . . . . . . . . . . . . . . . . . . 19

2.3.1 Gaussian filter . . . . . . . . . . . . . . . . . . . . . . . . . . . . . . . . . . 19

2.3.2 Non-local means (NLM) algorithm . . . . . . . . . . . . . . . . . . . . . . . . 19

2.3.3 Batch-matching and 3D filtering (BM3D) algorithm with Anscombe transformation 19

**Supplementary Note 3: Simulation** **20**

3.1 Simulated data generation . . . . . . . . . . . . . . . . . . . . . . . . . . . . . . . . 20

3.2 Accuracy metrics and statistical analysis in simulation . . . . . . . . . . . . . . . . . . 21

3.3 Hot pixel removal methods evaluation . . . . . . . . . . . . . . . . . . . . . . . . . . 22

3.3.1 Optimal iteration number selection and running time evaluation . . . . . . . . . . 22

3.3.2 Benchmark DIMR with other hot pixel removal methods . . . . . . . . . . . . . . 23

3.4 Shot noise filtering methods evaluation . . . . . . . . . . . . . . . . . . . . . . . . . . 26

3.4.1 Compare DeepSNiF with Noise2Void and modified Noise2Void . . . . . . . . . . 26

3.4.2 The effect of Hessian norm regularization on DeepSNiF . . . . . . . . . . . . . . 28

3.4.3 Compare DeepSNiF with traditonal denoising methods . . . . . . . . . . . . . . . 31

**Supplementary Note 4: Tutorial of IMC-Denoise software package** **32**

**Other Supplementary Figures** **34**

**Other Supplementary Tables** **69**

**References** **78**

# Supplementary Note 1: IMC-Denoise framework

## 1.1 Imaging mass cytometry (IMC) imaging model

In the IMC imaging process, there are three common noise sources: hot pixels, ion shot noise, and spillover. Hot pixels are concentrated areas of high counts which are uncorrelated with any biological structures. In IMC images this artifiact is most often observed as single hot pixels; however, small areas or clusters with several consecutive pixels may also be found. Ion shot noise exists because of the ion counting imaging process. The higher ion counts, the lower the shot noise level will be. Spillover, which is signal detected in one channel that is originating from an adjacent channel, can be neglected if the marker panel is well designed and properly titrated. Even with the existence of spillover, the contributions to total noise are very weak, which is approximately only one percent of the original intensity from the originating channel [1]. While applying low concentrations of staining antibodies minimizes spillover concerns, this will also result in even lower ion counts, and thus higher shot noise levels.

Here, we model the ion counting imaging as a Poisson process and hot pixels as outliers with much larger intensity than their adjacent pixels. As a result, the IMC imaging model is built as Eq. (1).

$$\mathbf{R} = \mathcal{P}[\mathbf{X} + \mathbf{X}^{\text{spillover}}] + \mathbf{Q}, \tag{1}$$

where $\mathbf{R} = \{r_p\}$ is the raw image set, $\mathbf{X} = \{x_p\}$ the true signals without noise, $\mathbf{X}^{\text{spillover}} = \{x_p^{\text{spillover}}\}$ the spillover signals without noise, $\mathbf{Q} = \{q_p\}$ the hot pixels, $p$ the pixel index and $\mathcal{P}[x]$ the Poisson noise with mean $x$.

In this paper, we only consider the hot pixels and ion shot noise in raw IMC images. If spillover is observed, it should be corrected after the restoration of these two noise sources, as its signal is contaminated by them as well. Therefore, the IMC imaging model is simplified as Eq. (2).

$$\mathbf{R} = \mathcal{P}[\mathbf{X}] + \mathbf{Q}, \tag{2}$$

## 1.2 Rationale for IMC denoising

### 1.2.1 Impact on pixel scale

First we consider a signal pixel $s$ and a background pixel $b$. Assume that the intensity of the background pixel $x_b>0$ because of unspecific staining or staining artifacts. Without noise, we have $x_s>x_b$. However, with the shot noise and hot pixel artifact, the two pixel values become $r_s = \mathcal{P}[x_s] + q_s$ and $r_b = \mathcal{P}[x_b] + q_b$. Because the Poisson model can be feasibly estimated as a Gaussian process [2], Eq. (2) can be converted as

$$\mathbf{R} = \mathcal{N}(\mathbf{X}, \mathbf{X}) + \mathbf{Q}. \tag{3}$$

As a result, the two pixel values are approximated as $x_s + \mathcal{N}(0, x_s) + q_s$ and $x_b + \mathcal{N}(0, x_b) + q_b$. Thus, the noise terms of pixels $s$ and $b$ are $\mathcal{N}(0, x_s) + q_s$ and $\mathcal{N}(0, x_b) + q_b$, respectively. With the impact of the noise terms, it is possible that $r_s<r_b$, resulting in error detection of IMC signals. Specifically, the signal-to-noise ratio (SNR) for the shot noise can be defined as

$$\text{SNR} = \frac{x}{\sqrt{x}} = \sqrt{x}. \tag{4}$$

Therefore, the lower the signal $x$, the lower SNR and the higher chance $r_s<r_b$ will be.

### 1.2.2 Impact on cell scale

Subsequently, we consider a positive cell with marker intensity $\frac{1}{M}\sum_{i=1}^{M} x_i$ and a negative cell with intensity $\frac{1}{N}\sum_{j=1}^{N} x_j$, where $M$ and $N$ are the pixel numbers of these two cell masks, respectively. Without the noise impact, we can assume $\frac{1}{M}\sum_{i=1}^{M} x_i > \frac{1}{N}\sum_{j=1}^{N} x_j$ even with the existance of unspecific staining. However, after contaminated by the shot and hot pixel noises, the two terms become

$$\frac{1}{M}\sum_{i=1}^{M} r_i = \frac{1}{M}\sum_{i=1}^{M}(\mathcal{P}[x_i] + q_i). \tag{5}$$

$$\frac{1}{N}\sum_{j=1}^{N} r_j = \frac{1}{N}\sum_{j=1}^{N}(\mathcal{P}[x_j] + q_j). \tag{6}$$

Similar to pixel scale analysis, these two terms can be further approximated as

$$\frac{1}{M}\sum_{i=1}^{M} r_i = \frac{1}{M}\sum_{i=1}^{M} x_i + \mathcal{N}(0, \frac{1}{M}\sum_{i=1}^{M} x_i) + \frac{1}{M}\sum_{i=1}^{M} q_i. \tag{7}$$

$$\frac{1}{N}\sum_{j=1}^{N} r_j = \frac{1}{N}\sum_{j=1}^{N} x_j + \mathcal{N}(0, \frac{1}{N}\sum_{j=1}^{N} x_j) + \frac{1}{N}\sum_{j=1}^{N} q_j. \tag{8}$$

Under this condition, it is possible that $\frac{1}{M}\sum_{i=1}^{M} r_i < \frac{1}{N}\sum_{j=1}^{N} r_j$ such that a positive cell is falsely regarded as a negative one or a negative cell is falsely detected as a positive one. Also similar to the pixel scale analysis, for shot noise the lower the signal values, the higher chance the detection errors will occur.

To summarize, the noise sources can result false detection of signal and positive cell markers, so as to impact downstream analysis. Therefore, it is essential to develop algorithms to filter the hot pixel artifact and to account for the shot noise.

## 1.3 Differential intensity map-based restoration algorithm for hot pixel removal (DIMR)

### 1.3.1 Hot pixel unmixing

From Eq. (3), larger true signal $\mathbf{X}$ will result in larger variance. Thus, the contaminated signal $\mathcal{P}[\mathbf{X}]$ with larger $\mathbf{X}$ is more likely to be considered as hot pixels $\mathbf{Q}$ and vice versa. To avoid such false detection, we stabilize the variance of the signal $\mathbf{X}$ with the Anscombe transformation [3] as Eq. (9).

$$\mathbf{R}' = \mathbf{X}' + \mathcal{N}(0, 1) + \mathbf{Q}', \tag{9}$$

where $\mathcal{N}(0, 1)$ is the additive noise with standard Gaussian distribution, and $\mathbf{R}'$, $\mathbf{X}'$ and $\mathbf{Q}'$ are the transformed raw image, "clean" signal and hot pixels, respectively. As hot pixels are local maxima in IMC images, we detect them by comparing adjacent pixels in a 3×3 sliding window. Considering the nonlinearity of the Anscombe transformation [4], pixels with intensities lower than 4 in $\mathbf{R}$ are omitted directly in order to exclude the impact of background regions, which cannot be outliers. Additionally, the difference between adjacent pixels can be fitted as a generalized Gaussian distribution [5]. Thus, in the 3×3 sliding window, we derive Eq. (10) by calculating the differences between the center pixel and its 8 neighbours. In Eq. (10), $i$ is the neighbour index in the sliding window ($i \in \{1, 2, ..., 8\}$), $\mathcal{G}(\mu, \alpha, \beta)$ is a generalized

Gaussian distribution with location $\mu$, scale $\alpha$ and shape $\beta$, and $\mathbf{R}' - \mathbf{R}_i''$, $\mathbf{X}' - \mathbf{X}_i''$ and $\mathbf{Q}' - \mathbf{Q}_i''$ are the differences of the raw image, "clean" signal and hot pixels in the $i$-th direction, respectively. Without the hot pixel component $\mathbf{Q}' - \mathbf{Q}_i''$, $\mathbf{D}_i$ can also be approximated as a generalized Gaussian distribution.

$$\begin{aligned} \mathbf{D}_i &= \mathbf{R}' - \mathbf{R}_i'' = \mathbf{X}' - \mathbf{X}_i'' + \mathcal{N}(0,2) + \mathbf{Q}' - \mathbf{Q}_i'' \\ &= \mathcal{G}(\mu_i, \alpha_i, \beta_i) + \mathcal{N}(0,2) + \mathbf{Q}' - \mathbf{Q}_i''. \end{aligned} \tag{10}$$

Similar to fluorescence microscopy, in IMC images the tissue or background pixels should be continuous. Thus, for any normal pixel $p$ the distance between $d_i^p$ and the mean $\mu_i$ is always less than that of a single hot pixel, where $d_i^p$ is the pixel $p$'s value in the distribution $\mathbf{D}_i$. Therefore, we can define $\triangle_i^p = |d_i^p - \mu_i|$ as the measure to determine whether a pixel $p$ is a hot pixel. However, this might not hold for consecutive hot pixels. For instance, if two consecutive hot pixels sharing similar intensities, their difference may be very close to $\mu_i$. To detect consecutive hot pixels, it is reasonable to assume there are at least $l$ pixels close to a normal pixel $p$. $l$ is normally set as 4, which corresponds to half neighbours. Consequently, we sort $\triangle_i^p$ for every pixel $p$ in an ascending direction as Eq. (11).

$$\triangle_{(i)}^p = \text{sort}(\triangle_i^p), \tag{11}$$

where $(i)$ is the sorted index and $i \in \{1, 2, ..., 8\}$. Then we define the sum of the first $l$ $\triangle_{(i)}^p$ as Eq. (12).

$$s_l^p = \sum_{i=1}^{l} \triangle_{(i)}^p, \tag{12}$$

For a normal pixel $p$, its $s_l^p$ should be less than that of a hot pixel. Because $s_l^p$ measures the relationship between the center pixel and its multiple neighbours, it is more robust than a single $\triangle_i^p$, especially for consecutive hot pixels.

Due to the spatial continuity and isotropic resolution of IMC images, $\mu_i$ from different directions can be regarded as equal to each other. For the sake of simplicity, we define $\mu_i = \mu$ for $i \in \{1, 2, ..., 8\}$. To further separate normal and hot pixels, Eq. (13) is consequently derived based on the triangle inequality [6].

$$s_l^p = \sum_{i=1}^{l} \left| d_{(i)}^p - \mu \right| \geq \left| t_l^p - l\mu \right|, \tag{13}$$

where $t_l^p = \sum_{i=1}^{l} d_{(i)}^p$. The equality holds only when $d_{(i)}^p \geq \mu$ or $d_{(i)}^p \leq \mu$ for all $i \in \{1, 2, ..., l\}$. The first case is always true for single hot pixels and pixels with the largest intensities in hot clusters. Otherwise, $t_l^p$ will shrink towards $l\mu$. Therefore, $t_l^p$ will further unmix hot pixels from normal ones. Combining all the $t_l^p$, a new distribution $\mathbf{T}_l$ is generated, and outliers are located beyond its right tail. In fact, some consecutive hot pixels can shrink towards $l\mu$ as well. For example, in the case of a hot pixel that is larger than all of its normal neighbours but smaller than the largest hot pixel in the 3×3 sliding window, it is possible that $t_{l-1}^p - (l-1)\mu = \mu - d_{(l)}^p > 0$. To solve this issue, we implement multiple iterations of the sorting to adequately remove the hot pixel noise. Normally the iteration number $N_{\text{iter}}$ is set as 3 such that at least 3 consecutive hot pixels will be removed after 3 iterations.

### 1.3.2 Hot pixel detection

In order to search for outliers, the shape of $\mathbf{T}_l$ should be investigated first. Therefore, let us also define $u_l^p = \sum_{i=1}^{l} d_i^p$, and combine $u_l^p$ from all pixels to form a distribution $\mathbf{U}_l$. Without hot pixels, $\mathbf{U}_l$ is approximately a generalized Gaussian distribution with mean $l\mu$. Because

$$\sum_p \sum_{i=1}^{l} (\triangle_{(i)}^p)^2 \leq \sum_p \sum_{i=1}^{l} (\triangle_i^p)^2, \tag{14}$$

where the two items are proportional to the variances of $\mathbf{T}_l$ and $\mathbf{U}_l$, respectively, $\mathbf{T}_l$ can be regarded as a generalized Gaussian distribution with mean $l\mu$ and smaller variance than $\mathbf{U}_l$.

Because the histogram of $\mathbf{T}_l$ is discretized, we apply the kernel density estimation algorithm [7], as Eq. (15) shows, to fit a continuous curve.

$$\hat{g}_h(x) = \frac{1}{mh} \sum_{i=1}^{m} K\left(\frac{x - x_i}{h}\right), \tag{15}$$

where $K$ is a Gaussian kernel, $h$ the bandwidth, $m$ the point number and $x_i$ the sampled points. The bandwidth $h$ is set as $1.06\hat{\sigma}l^{-\frac{1}{5}}$ [7], where $\hat{\sigma}$ is the standard deviation of the sampled points. The point interval is set as 1 for adequate sampling as well as avoiding comb-like structures. Subsequently, a moving mean filter with window size of 3 is optionally utilized to further eliminate minor fluctuations of the fitted curve. With the fitted curve $(x, \hat{g}_h(x))$, a threshold point $x_T$ is defined and then any points $x > x_T$ are considered as outliers. Because outliers are located beyond the right tail of $\mathbf{T}_l$, it is reasonable to set $x_T$ when $\frac{d\hat{g}_h(x)}{dx} \to 0$, which means the current distribution ends. Likewise, the shape of the curve will not change from convex

to concave on its right tail. Thus, it is also reasonable to set $x_T$ when $\frac{d^2\hat{g}_h(x-\triangle x)}{dx^2} \geq 0$ and $\frac{d^2\hat{g}_h(x)}{dx^2} \leq 0$, where $\triangle x$ represents a small value. Because the pixel values of the raw images are discrete, $\triangle x$ is normally set as 1. The detected outliers are filtered by a $3\times3$ median filter in each iteration. When the iterations terminate, the image is transformed to its original scale by the direct algebraic inverse Anscombe transformation [8]. Additionally, we substitute the mean $\mu_i^p$ of each distribution $\mathbf{D}_i$ with the median $\tilde{\mu}_i^p$ when implementing this algorithm, as median is more robust than mean when encountering outliers.

The DIMR algorithm is summarized as Supplementary Algorithm 1 and Supplementary Figure 1.

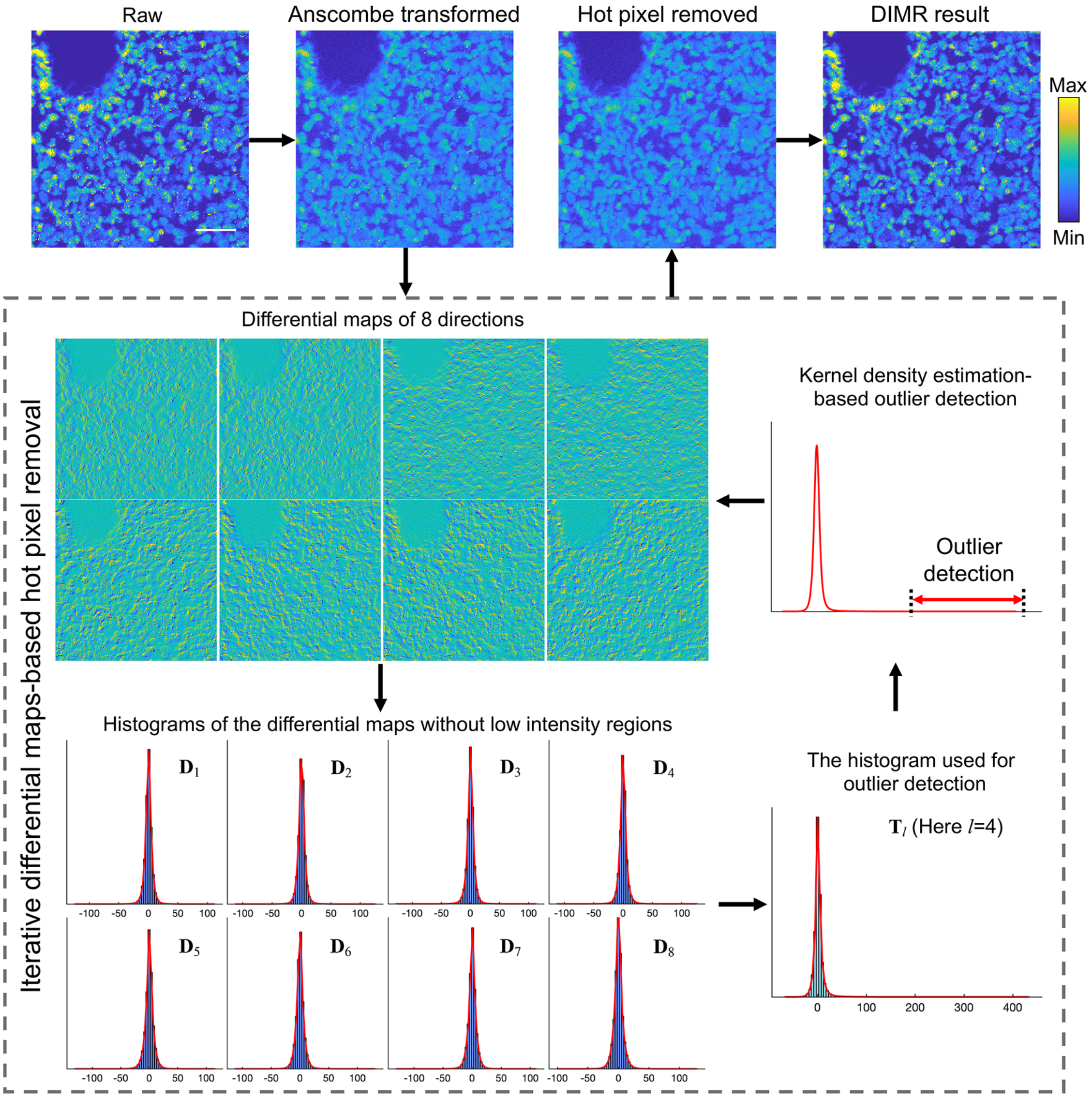


**Supplementary Figure 1.** The schematic of the DIMR algorithm. Scale bar: 74 $\mu$m.

**Supplementary Algorithm 1** DIMR algorithm

**Input:** Raw image **R**;
Hyperparameters $l$ and $N_{\text{iter}}$;
**Output:** Hot pixel removed image;

Apply the Anscombe transformation to **R**;
**for** each $k$=1, 2, ..., $N_{\text{iter}}$ **do**
    Calculate the 8 differential maps of the Anscombe-transformed raw image $\mathbf{R}'$ in a sliding 3×3 window;
    Remove the pixels with value less than 4 for each differential map and form the distributions $\mathbf{D}_i$ where $i \in \{1, 2, ..., 8\}$;
    For every remaining pixel $p$, calculate $\triangle_i^p = |d_i^p - \tilde{\mu}_i|$, where $\tilde{\mu}_i$ is the median of $\mathbf{D}_i$;
    Sort all the $\triangle_i^p$ and get the corresponding sorted index $(i)$ for every pixel $p$. With the index $(i)$, calculate $t_l^p$ for each pixel $p$ to form a new distribution $\mathbf{T}_l$;
    Apply the kernel density estimation algorithm (Eq. (15)) to generate a continuous curve $(x, \hat{g}_h(x))$ from the histogram of $\mathbf{T}_l$, followed by moving mean filtering with window size of 3 (optional).
    Starting from the right tail of the curve, if (1) $\frac{d\hat{g}_h(x)}{dx} \to 0$ or (2) $\frac{d^2\hat{g}_h(x-\triangle x)}{dx^2} \geq 0$ and $\frac{d^2\hat{g}_h(x)}{dx^2} \leq 0$, set the point $x$ as $x_T$;
    **if** There is no $x > x_T$ **then**
        Break
    **else**
        Set any points $x > x_T$ as outliers and filter the corresponding pixels with a 3×3 median filter.
    **end if**
**end for**
Apply the direct algebraic inverse Anscombe transformation to the processed image.

## 1.4 Self-supervised deep learning-based algorithm for shot noise image filtering (DeepSNiF)

### 1.4.1 Optimal loss function derivation

After hot pixel removal, the IMC imaging model is simplified as Eq. (16).

$$\mathbf{R} = \mathcal{P}[\mathbf{X}]. \tag{16}$$

In a supervised learning framework, the noisy and clean images **R** and **X** are both known. Therefore, for all the pixels $p$ from 1 to $L$, pairs of $(r_p, x_p)$ are formed as a training set. A deep convolutional neural network with forward model $\mathcal{F}_\theta$ ($\mathbb{E}^L \mapsto \mathbb{E}^L$) is then built to filter the Poisson noise such that $\mathbf{X} = \mathcal{F}_\theta[\mathbf{R}]$, where $\theta$ reflects the parameters of the neural network. Ideally, $\mathcal{F}_\theta[\mathbf{R}]_p$ should be identical to that of $x_p$ for all pixels. In this sense, the optimal estimator for denoising can be derived through a binary signal detection task. This task requires an observer to classify the denoised signals $\mathcal{F}_\theta[\mathbf{R}]_p$under a hypothesis $H_0$ from the true clean signals $x_p$ under another hypothesis $H_1$. Given noisy data $r_p$, these two hypotheses can be

described as:

$$H_0 : \text{denoised signals} : \mathcal{F}_\theta[\mathbf{R}]_p, 1 \le p \le L$$
$$H_1 : \text{true signals} : x_p, 1 \le p \le L \tag{17}$$

Because the IMC data follows a Poisson distribution, the corresponding likelihood functions for these two hypotheses are expressed as Supplementary Eqs. (18) and (19). From Eq. (19), $x_p$ can be regarded as the maximum likelihood estimation (MLE) solution of $r_p$.

$$\Pr(\mathbf{R}|\mathcal{F}_\theta[\mathbf{R}]) = \prod_{p=1}^{L} \exp(-\mathcal{F}_\theta[\mathbf{R}]_p)\mathcal{F}_\theta[\mathbf{R}]_p^{r_p}/r_p!. \tag{18}$$

$$\Pr(\mathbf{R}|\mathbf{X}) = \prod_{p=1}^{L} \exp(-x_p)x_p^{r_p}/r_p!. \tag{19}$$

The log-likelihood functions of the two hypotheses are derived as Supplementary Eqs. (20) and (21).

$$\log[\Pr(\mathbf{R}|\mathcal{F}_\theta[\mathbf{R}])] = \sum_{p=1}^{L}(r_p \log(\mathcal{F}_\theta[\mathbf{R}]_p) - \mathcal{F}_\theta[\mathbf{R}]_p - \log r_p!). \tag{20}$$

$$\log[\Pr(\mathbf{R}|\mathbf{X})] = \sum_{p=1}^{L}(r_p \log(x_p) - x_p - \log r_p!). \tag{21}$$

Then, we define the log-likelihood ratio $\mathcal{L}(H_0, H_1)$ to measure the difference between the two hypotheses:

$$\begin{aligned}\mathcal{L}(H_0, H_1) &= \log[\Pr(\mathbf{R}|\mathbf{X})] - \log[\Pr(\mathbf{R}|\mathcal{F}_\theta[\mathbf{R}])] \\ &= \sum_{p=1}^{L}(r_p \log \frac{x_p}{\mathcal{F}_\theta[\mathbf{R}]_p} - x_p + \mathcal{F}_\theta[\mathbf{R}]_p).\end{aligned} \tag{22}$$

The learning process aims to train $\mathcal{F}_\theta$ such that $x_p = \mathcal{F}_\theta[\mathbf{R}]_p$ for all the $p$ from 1 to $L$. Thus, the expectation of this loss function $\mathcal{L}$ under hypothesis $H_1$, $E[\mathcal{L}(H_0, H_1)|H_1]$, as Eq. (23) demonstrates, should be as small as possible. In that sense, the observer in the binary detection task will be more difficult to differ these two hypotheses as $E[\mathcal{L}(H_0, H_1)|H_1]$ decreases. When $E[\mathcal{L}(H_0, H_1)|H_1] = 0$, the two hypotheses are identical and the denoised signal $\mathcal{F}_\theta[\mathbf{R}]_p$ will be identical to the true signal $x_p$ for all the pixels, which means

$\mathcal{F}_\theta[\mathbf{R}]_p$ will be the MLE solution of $r_p$ as well.

$$
\begin{aligned}
E[\mathcal{L}(H_0,H_1)|H_1] &= \sum_{p=1}^{L}(E[r_p|H_1]\log\frac{x_p}{\mathcal{F}_\theta[\mathbf{R}]_p} - x_p + \mathcal{F}_\theta[\mathbf{R}]_p) \\
&= \sum_{p=1}^{L}(x_p\log\frac{x_p}{\mathcal{F}_\theta[\mathbf{R}]_p} - x_p + \mathcal{F}_\theta[\mathbf{R}]_p).
\end{aligned} \tag{23}
$$

Eq. (23) is generally known as I-divergence [9]. Here, we set it as the loss function, and thus the optimal parameter of the network $\hat{\theta}^*$ is defined as Eq. (24).

$$
\hat{\theta}^* = \operatorname{argmin}_\theta \sum_{p=1}^{L}(x_p\log\frac{x_p}{\mathcal{F}_\theta[\mathbf{R}]_p} - x_p + \mathcal{F}_\theta[\mathbf{R}]_p). \tag{24}
$$

Due to the difficulties associated with acquiring high SNR images as ground truths and the impossibility to repetitively scan the same tissue in IMC, a supervised learning approach is unavailable here. Fortunately, we find self-supervised approaches are also qualified for this denoising task. Let us define a function $f$ demonstrating a random pixel masking approach for images such as those in Noise2Void [10] and Noise2Self [11]. In such strategies, multiple pixels are randomly masked and replaced by their adjacent pixels or random values. The new pixel value $f(\mathbf{R})_p$ at pixel $p$ can be regarded as the true value $x_p$ contaminated by another noise process. In this case, the true value $x_p$ can be predicted by the adjacent pixels because of the spatial continuity of IMC images. Therefore, in a self-supervised learning problem, the loss function will be

$$
\begin{aligned}
\mathcal{L}(\mathbf{R},\mathcal{F}_\theta[f(\mathbf{R})]) &= \sum_{p=1}^{L}(r_p\log\frac{r_p}{\mathcal{F}_\theta[f(\mathbf{R})]_p} - r_p + \mathcal{F}_\theta[f(\mathbf{R})]_p) \\
&= \sum_{p=1}^{L}(r_p\log r_p - (x_p + r_p - x_p)\log\mathcal{F}_\theta[f(\mathbf{R})]_p - (x_p + r_p - x_p) + \mathcal{F}_\theta[f(\mathbf{R})]_p) \\
&= \sum_{p=1}^{L}(x_p\log\frac{x_p}{\mathcal{F}_\theta[f(\mathbf{R})]_p} - x_p + \mathcal{F}_\theta[f(\mathbf{R})]_p) + \sum_{p=1}^{L}(r_p\log r_p - x_p\log x_p) \\
&\quad - \sum_{p=1}^{L}(r_p - x_p) - \sum_{p=1}^{L}(r_p - x_p)\log\mathcal{F}_\theta[f(\mathbf{R})]_p.
\end{aligned} \tag{25}
$$

In order to ensure that the training process with the self-supervised learning loss is identical to that with known true signals, the last three terms in Eq. (25) should be constants. Obviously, the second term

$\sum_{p=1}^{L}(r_p \log r_p - x_p \log x_p)$ fulfills this requirement. On the other hand, in the last two terms, $r_p - x_p$ and $\mathcal{F}_\theta[f(\mathbf{R})]_p$ are the noise component and predicted value of pixel $p$, respectively. Because the Poisson noise is a pixel-independent stochastic process and $\mathcal{F}_\theta[f(\mathbf{R})]_p$ is determined by its neighbours in the proposed self-supervised learning framework, they are uncorrelated with each other, except the masked pixel $p$ is replaced by itself. As a result, the last two term in Eq. (25) can be approximated as Eq. (26).

$$\begin{aligned}\sum_{p=1}^{L}(r_p - x_p) + \sum_{p=1}^{L}(r_p - x_p)\log\mathcal{F}_\theta[f(\mathbf{R})]_p &= \sum_{p=1}^{L}(r_p - x_p) + \sum_{p=1}^{L}(r_p - x_p)\sum_{p=1}^{L}\log\mathcal{F}_\theta[f(\mathbf{R})]_p \\ &= \sum_{p=1}^{L}(r_p - x_p)\left[1 + \sum_{p=1}^{L}\log\mathcal{F}_\theta[f(\mathbf{R})]_p\right].\end{aligned} \tag{26}$$

Because $E[r_p] = E[x_p]$ for a Poisson noise model, Eq. (26) is approximated as 0 when $L$ is large enough, also suggested by [11]. From this viewpoint, training with noisy images and their masked pairs is identical to training with noisy and clean image pairs, as long as the masked pixels are not replaced by themselves. To avoid learning identity in the training, only the loss of the masked pixels are considered, as Eq. (27) shows.

$$\mathcal{L}(\mathbf{R}, \mathcal{F}_\theta[f(\mathbf{R})]) = \sum_p \mathbf{M}_p \cdot \left[r_p \log\frac{r_p}{\mathcal{F}_\theta[f(\mathbf{R})]_p} - r_p + \mathcal{F}_\theta[f(\mathbf{R})]_p\right] \Big/ \sum_p \mathbf{M}_p, \tag{27}$$

where $\mathbf{M}_p$ is the pixel mask ($\mathbf{M}_p \in \{0, 1\}$). If $\mathbf{M}_p = 1$, then the pixel $p$ is masked; otherwise, it is not. To guarantee the output is non-negative, a softplus function $\log(1+\exp(x))$ is set as the activation of the network's output layer.

### 1.4.2 Hessian norm regularization as a booster for the denoising task

Even with the optimal estimator, the denoising performance is still sub-optimal: in the training process, the information of the masked pixels is always neglected; and only partial pixels are utilized. These limitations hinder the trained denoiser to further enhance its performance. To solve this issue, the characteristics of the IMC images should be utilized in the training process. As an imaging modality similar to traditional fluorescence microscopy, IMC is always used for detecting the phenotype of biological structures. Thus, the spatial continuity between biological structures can be used as a *priori*. In fact, the Hessian norm regularization is widely used to describe such spatial continuity in biological imaging data [2,12,13]. However, to the best of our knowledge, this advanced statistical prior has never been used in deep learning-based denoising task. To implement this prior in IMC images, let us first define the Hessian operator $\mathcal{R}_{\text{Hessian}}$ as

Eq.(28).

$$\mathcal{R}_{\text{Hessian}} = \begin{bmatrix} \partial_{xx} & \partial_{xy} \\ \partial_{yx} & \partial_{yy} \end{bmatrix}, \tag{28}$$

where $\partial_{xx} = \partial^2/\partial x^2$, $\partial_{xy} = \partial_{yx} = \partial^2/\partial x \partial y$, $\partial_{yy} = \partial^2/\partial y^2$, and $x$ and $y$ are the horizontal and vertical directions of an image, respectively. Then, for any estimated image $\mathcal{F}_\theta[f(\mathbf{R})]$, the corresponding Hessian norm regularization term is defined as Eq. (29).

$$||\mathcal{R}_{\text{Hessian}}(\mathcal{F}_\theta[f(\mathbf{R})])|| = |\partial_{xx}\mathcal{F}_\theta[f(\mathbf{R})]| + |\partial_{yy}\mathcal{F}_\theta[f(\mathbf{R})]| + \sqrt{2}|\partial_{xy}\mathcal{F}_\theta[f(\mathbf{R})]| \tag{29}$$

When involving the Hessian norm regularization as a prior, the optimization problem will convert from Eq. (23) to Eq. (30).

$$\hat{\theta}^* = \text{argmin}_\theta \sum_p \left[E[\mathcal{L}(H_0, H_1)|H_1] - \lambda_{\text{Hessian}} \log[\text{Pr}_{\text{Hessian}}(\mathcal{F}_\theta[f(\mathbf{R})]_p; \theta)]\right], \tag{30}$$

where $\text{Pr}_{\text{Hessian}}(\mathcal{F}_\theta[f(\mathbf{R})]_p; \theta) = \exp(-||\mathcal{R}_{\text{Hessian}}(\mathcal{F}_\theta[f(\mathbf{R})])||_p)$ and $\lambda_{\text{Hessian}}$ is the Hessian norm regularization parameter. Therefore, the loss function Eq. (27) will be

$$\begin{aligned} \mathcal{L}(\mathbf{R}, \mathcal{F}_\theta[f(\mathbf{R})]) = & \sum_p \mathbf{M}_p \cdot \left[r_p \log \frac{r_p}{\mathcal{F}_\theta[f(\mathbf{R})]_p} - r_p + \mathcal{F}_\theta[f(\mathbf{R})]_p\right] \Big/ \sum_p \mathbf{M}_p \\ & + \lambda_{\text{Hessian}} \sum_p ||\mathcal{R}_{\text{Hessian}}(\mathcal{F}_\theta[f(\mathbf{R})])||_p \Big/ \sum_p. \end{aligned} \tag{31}$$

Note that the first term works only for the selected masked pixels, while the second regularization term utilizes all the information of images. Thus, Eq. (31) overcomes the limitations of Eq. (27) and further enhances the performance of the denoiser.

### 1.4.3 Image normalization

For both training and prediction, it is important to normalize the input images to a common range. However, some cells or structures may still exhibit extremely bright signal even after hot pixel removal. Consequently, all input and output data are percentile-normalized between 0 and 1. IMC images are always

non-negative and consist of pixels with 0 value, this normalization is defined as Eq. (32).

$$\text{Normalization}(\mathbf{R};q) = \frac{\mathbf{R}}{1.1 \times \text{perc}(\mathbf{R},q)}, \tag{32}$$

where $\text{perc}(\mathbf{R};q)$ is the $q$-th percentile of all pixel values in the training set. We typically use values of $q \in [99.9, 99.999]$. Note that only in training phase any values which are larger than 1 are set as 1. After prediction, the denoised images are re-transformed to their original scales.

This normalization approach does not affect the selection of regularization parameter $\lambda_{\text{Hessian}}$. The Hessian operator (28) is a linear operator, so we have

$$|\mathcal{R}_{\text{Hessian}}(\alpha_{\text{scale}}\mathcal{F}_\theta[f(\mathbf{R})])| = \alpha_{\text{scale}}|\mathcal{R}_{\text{Hessian}}(\mathcal{F}_\theta[f(\mathbf{R})])|, \tag{33}$$

where $\alpha_{\text{scale}} = 1/(1.1 \times \text{perc}(\mathbf{R};q))$. From here, we are able to derive

$$\begin{aligned}
&\mathcal{L}(\alpha_{\text{scale}}\mathbf{R}, [\mathcal{F}_\theta[f(\mathbf{R})]]^{\text{scaled}}) \\
=&\sum_p \mathbf{M}_p \cdot \left[\alpha_{\text{scale}} r_p \log\frac{\alpha_{\text{scale}} r_p}{[\mathcal{F}_\theta[f(\mathbf{R})]]_p^{\text{scaled}}} - \alpha_{\text{scale}} r_p + [\mathcal{F}_\theta[f(\mathbf{R})]]_p^{\text{scaled}}\right] \Big/ \sum_p \mathbf{M}_p \\
&+ \lambda_{\text{Hessian}} \sum_p ||\mathcal{R}_{\text{Hessian}}(\mathcal{F}_\theta[f(\mathbf{R})])||_p \Big/ \sum_p \\
=&\alpha_{\text{scale}}\Bigg\{\sum_p \mathbf{M}_p \cdot \left[r_p \log\frac{r_p}{[\mathcal{F}_\theta[f(\mathbf{R})]]_p^{\text{scaled}}/\alpha_{\text{scale}}} - r_p + [\mathcal{F}_\theta[f(\mathbf{R})]]_p^{\text{scaled}}/\alpha_{\text{scale}}\right] \Big/ \sum_p \mathbf{M}_p \\
&+ \lambda_{\text{Hessian}} \sum_p ||\mathcal{R}_{\text{Hessian}}(\mathcal{F}_\theta[f(\mathbf{R})]/\alpha_{\text{scale}})||_p \Big/ \sum_p \Bigg\}.
\end{aligned} \tag{34}$$

Therefore, $[\mathcal{F}_\theta[f(\mathbf{R})]]_p^{\text{scaled}} = \alpha_{\text{scale}}\mathcal{F}_\theta[f(\mathbf{R})]_p$ and the normalization does not affect the strength of regularization.

The DeepSNiF algorithm is summarized as Supplementary Algorithm 2.

**Supplementary Algorithm 2** DeepSNiF algorithm

**Input:** Hot pixel removed images $\mathbf{R}$;

**Output:** Noise filtered images;

1: Generate a training set for a specific hot pixel removed marker channel, in which all the images are percentile normalized between 0 and 1 with Eq. (32);

2: Train a denoising network for the marker channel with Eq. (31) as the loss function;

3: Normalize the hot pixel removed images with the pre-calculated maximum of the training set;

4: Filter shot noise for the normalized images with the trained network;

5: De-normalize the predicted images to their original scales.

# Supplementary Note 2: Reference methods

## 2.1 Hot pixel removal methods

Currently, two thresholding methods are mostly applied to remove hot pixels, which are neighbour-based threshold hot pixel removal method [14–16] (NTHM) and median-based threshold hot pixel removal method [17] (MTHM). NTHM is simple and straightforward. It presets a threshold $\sigma_{\text{thresh}}$. Then single pixels with intensity greater than this threshold of the maximum value in its $3\times3$ neighbourhood are detected as outliers. This method is very similar to our DIMR algorithm with hyperparameter $l = 1$, which will always overlook consecutive hot pixels. In addition, $\sigma_{\text{thresh}}$ needs to be manually set. However, the hot pixels in different tissues and channels have different scales. That is, one fixed threshold does not work for different images. These limitations downgrades its performance in real applications. The NTHM algorithm is summarized as Supplementary Algorithm 3, and the default value of $\sigma_{\text{thresh}}$ is set 50.

**Supplementary Algorithm 3** NTHM for hot pixel removal

**Input:** Raw image **R**;
Hyperparameter $\sigma_{\text{thresh}}$;
**Output:** Hot pixel removed image;
1: In a sliding $3\times3$ window, select the maximum value after excluding the center pixel;
2: **if** The difference between the center pixel and the maximum value is larger than $\sigma_{\text{thresh}}$ **then**
3: Substitute the center pixel's value with the maximum value.
4: **end if**

MTHM is an automated approach and can remove consecutive hot pixels, of which two thresholds $\sigma_{\text{thresh1}}$ and $\sigma_{\text{thresh2}}$ are needed to be manually set. This method first searches the top $\sigma_{\text{thresh1}}$ pixels and regards any pixels as outliers if they are $\sigma_{\text{thresh2}}$ times higher than the median in a sliding $5\times5$ window. However, this method may falsely remove normal pixels located at the border between tissues and background. Additionally, with a larger $\sigma_{\text{thresh1}}$ or smaller $\sigma_{\text{thresh2}}$, false negatives may also be generated. Normally, $\sigma_{\text{thresh1}}$ and $\sigma_{\text{thresh2}}$ are set as 2% and 4 separately [17]. This algorithm is summarized as Supplementary Algorithm 4. The default values of $\sigma_{\text{thresh1}}$ and $\sigma_{\text{thresh2}}$ are set as 2% and 4, respectively.

**Supplementary Algorithm 4** MTHM for hot pixel removal

**Input:** Raw image $\mathbf{R}$;

Hyperparameters $\sigma_{\text{thresh1}}$ and $\sigma_{\text{thresh2}}$;

**Output:** Hot pixel removed image;

1: Search all the pixels with top $\sigma_{\text{thresh1}}$ values in an image;

2: **if** The pixels are larger than $\sigma_{\text{thresh2}}$ times of the medians in their 5×5 window **then**

3: Substitute the pixels' values with the medians.

4: **end if**

## 2.2 Deep learning-based shot noise filtering methods

### 2.2.1 Noise2Void

Noise2Void uses noisy images as the input and output to train a denoising network. It randomly masks several pixels and replaces them with their neighbours. As Eq. (35) shows, minimizing the mean squared error (MSE) between the noisy image pairs is equal to minimizing that of the noisy and clean image pairs plus the variance of the noise. This equality holds as long as the function $\mathcal{F}$ is "$\mathcal{J}$-invariant" [11], which means $\mathcal{F}[f(\mathbf{R})]_p - x_p$ and $r_p - x_p$ are independent for any pixel $p$. Notably, the current masking strategies always fulfill this requirement if the noise is pixel-independent and the pixels are not replaced by themselves.

$$\mathcal{L}_{\text{MSE}}(\mathbf{R}, \mathcal{F}_\theta[\mathbf{R}_{\text{masked}}]) = E(\mathcal{F}_\theta[f(\mathbf{R})]_p - r_p)^2 \tag{35}$$

$$= E(\mathcal{F}_\theta[f(\mathbf{R})]_p - x_p)^2 + E(r_p - x_p)^2 \tag{36}$$

In Eq. (36), the second term corresponds to the noise variance at pixel $p$, which is a constant for a Gaussian noise model. In this condition, it can be neglected in the training process, and hence the self-supervised learning loss is equal to a supervised one.

However, this statement is not true for a Poisson noise model. The variance of Poisson noise is equal to its true signal intensity, i.e., $E(r_p - x_p)^2 = E(x_p)$. Therefore, the pixels with higher signal will gain more attention in Eq. (36), resulting in sub-optimal denoising for signal-limited area. In fact, even in a supervised learning task, MSE is still not an unbiased estimator for Poisson noise. When true signals are available, the optimizer can be derived by the MLE framework:

$$\hat{\theta}_{\text{MLE}} = \text{argmin}_\theta E\big[-\log\big(\Pr(\mathcal{F}_\theta(\mathbf{R})_p | x_p; \theta)\big)\big]. \tag{37}$$

Approximating a Poisson process as Gaussian distribution, Eq. (37) is converted as

$$\hat{\theta}_{\mathrm{MLE}} = \mathrm{argmin}_{\theta} E\big[\frac{1}{2}(\mathcal{F}_{\theta}(\mathbf{R})_p - x_p)^2/x_p + \frac{1}{2}\log(2\pi x_p)\big]. \tag{38}$$

Obviously, minimizing this loss in Eq. (38) is not equivalent to minimizing the MSE between $\mathcal{F}_{\theta}(\mathbf{R})_p$ and $x_p$, because $x_p$ varies with different pixels $p$. As a result, MSE loss will generate bias in Poisson denoising. Beside this issue, in the original version of Noise2Void, a linear activation is used as the output layer's activation function of the network, which violates the non-negativity of IMC images.

#### 2.2.2 Modified Noise2Void with the Anscombe transformation and rectified linear unit (ReLU) activation

We propose a modified Noise2Void algorithm to correct the bias of Noise2Void. The Anscombe transformation is first applied to the IMC data, so that the Poisson noise is approximated as a Gaussian-distributed noise model. In this sense, the noise variance term in Eq. (35) can be regarded as a constant and the bias from MSE loss will be mitigated. By substituting linear activation with ReLU [18], the non-negativity of IMC images is satistied. Note that the denoised images should be re-transformed to their original scale by the exact unbiased inverse Anscombe transformation [8]. The exact unbiased inverse Anscombe transformation software package (v1.0.0) was downloaded from https://webpages.tuni.fi/foi/invansc/.

However, the Anscombe transformation is not accurate for very low values [4]. Unfortunately, there are usually a portion of pixels in IMC images suffering from extremely low counts. The variances of these pixels are still positively correlated with the counts. Thus, even this approach reduces the bias of Noise2Void, some errors are still inevitable.

Compared to the MSE loss used by Noise2Void and Noise2Self, our derived loss function is the optimal estimator for Poisson denoising and the corresponding outputs are inherently non-negative with a softplus function. Consequently, it does not generate any biases for IMC denoising.

#### 2.2.3 Noise2True

In Noise2True [19], clean images are available as ground truths, so that supervised learning is possible. To achieve a learning with the optimal estimator, we use our derived I-divergence Eq. (23) as the loss

function in Noise2True. In simulation, we compare all the above denoising methods with Noise2True. We are especially curious as to how our Hessian norm regularization could help boost self-supervised learning to approach the performance of Noise2True. The ground truths of IMC images can only be acquirde in simulation. Thus, Noise2True is in fact not possible in real IMC denoising.

## 2.3 Traditional statistics-based shot noise filtering methods

### 2.3.1 Gaussian filter

Gaussian filter might be the most widely used noise filter. For all of our IMC images, we apply a Gaussian filter with kernel size of $5\times5$ and standard deviation of 0.8. Gaussian filter can only remove the high-frequency noise with the risk of filtering lots of true signal. As a result, its performance is sub-optimal than other smarter denoising algorithms.

### 2.3.2 Non-local means (NLM) algorithm

NLM algorithm [20] takes a mean of all pixels in an image, weighted by how similar these pixels are to the target pixel. In our paper, this algorithm is implemented using the Matlab built-in function "imnlmfilt". All the parameters are set as default.

### 2.3.3 Batch-matching and 3D filtering (BM3D) algorithm with Anscombe transformation

BM3D [21] is modified from NLM algorithm, which is usually regarded as a state-of-the-art denoising algorithm due to its superior performance in multiple applications. Instead of simply averaging the pixel values, it collects similar patches of an image, and then applies hard thresholding and Wiener filtering in two stages. BM3D is built on a white Gaussian noise model. Therefore, the Anscombe transformation is applied to approximate the Poisson noise in IMC images to Gaussian noise. The same applies for the modified Noise2Void algorithm, the exact unbiased inverse Anscombe transformation is used to rescale the denoised images.

The BM3D algorithm software package (v3.0.9) was downloaded from `https://webpages.tuni.fi/foi/GCF-BM3D/index.html#ref_software`. When implementing BM3D, we set $\sigma_{\text{noise}} = 1$, $N_2 = 8$, $N_s = 17$, $\tau_{\text{match}} = 2500$, $\lambda_{\text{thr3D}} = 1$, $Ns_{\text{wiener}} = 25$ and $\tau_{\text{match-weiner}} = 600$. All the other parameters were set as default.

# Supplementary Note 3: Simulation

Due to the difficulty to acquire ground truths, it is infeasible to quantitatively evaluate the accuracy of hot pixel removal methods and the shot noise filtering algorithms using real IMC images. As a consequence, we propose to conduct a comprehensive and accurate quantitative evaluation with simulated data.

## 3.1 Simulated data generation

We modify Eq. (2) as Eq. (39) to generate simulated data.

$$\mathbf{R} = \mathcal{P}[\mathbf{X}_{\text{origin}}/\gamma] + \mathbf{Q}, \tag{39}$$

where $\mathbf{X}_{\text{origin}}$ represent the original clean images used for simulation and $\gamma$ is the scale factor to control the overall ion counts level. A larger $\alpha$ indicates the overall ion counts of the ground truth image $\mathbf{X}_{\text{GT}} = \mathbf{X}_{\text{origin}}/\gamma$ is lower, and hence the shot noise level is higher. On the other hand, we assume hot pixels $\mathbf{Q}$ obey a negative binomial distribution $\text{NB}(\tau, \eta)$. Additionally, another parameter $\omega$ is used to indicate the density of hot pixels.

Here we select the original clean images $\mathbf{X}_{\text{origin}}$ from the *t*-CyCIF dataset [22] because of their very high SNR and similar resolution (1.06 $\mu$m) with IMC images. In particular, we choose a cell marker CD14 and structural marker Keratin from a lung tissue to evaluate the shot noise filtering algorithms. These two channels along with a DNA channel are used to evaluate the hot pixel removal methods.

We have four parameters $\gamma$, $\tau$, $\eta$ and $\omega$ to determine the noise conditions of the simulated images. For each channel, we set 4 different $\gamma$ which correspond to 4 different SNR levels including high, medium, low and very low. With different noise levels we set different $\tau$, $\eta$ and $\omega$ to represent different conditions of hot pixels. All the parameters are listed in Supplementary Table 1.

**Supplementary Table 1.** Simulation parameters

| Noise settings | | 1 | | | 2 | | | 3 | | | 4 | | |
|---|---|---|---|---|---|---|---|---|---|---|---|---|---|
| SNR levels | | high | | | medium | | | low | | | very low | | |
| Parameters | $\tau$ | $\gamma$ | $\eta$ | $\omega$ | $\gamma$ | $\eta$ | $\omega$ | $\gamma$ | $\eta$ | $\omega$ | $\gamma$ | $\eta$ | $\omega$ |
| CD14 & Keratin | 3 | 2000 | 0.05 | 1% | 5000 | 0.1 | 0.1% | 10000 | 0.2 | 0.01% | 16000 | 0.3 | 0.01% |
| DNA | 5 | 500 | 0.05 | 3% | 1000 | 0.1 | 1% | 2000 | 0.2 | 0.1% | 5000 | 0.25 | 0.01% |

## 3.2 Accuracy metrics and statistical analysis in simulation

In simulation, the root mean squared error (RMSE) is used to evaluate the performance of the hot pixel removal methods, as Eq. (40) shows, in which $p$ is the pixel index, $\mathbf{Y}^{\text{clean}}$ and $\mathbf{Y}^{\text{HM}}$ are the simulated images without hot pixels and hot pixel removed images, separately.

$$\text{RMSE}(\mathbf{Y}^{\text{HM}}, \mathbf{Y}^{\text{clean}}) = \sqrt{\frac{1}{L}\sum_{p=1}^{L}(\mathbf{Y}_p^{\text{HM}} - \mathbf{Y}_p^{\text{clean}})^2} \tag{40}$$

The peak SNR (PSNR) and structural similarity (SSIM) [23] are used to evaluate the performance of the shot noise filtering algorithms. PSNR indicates the ratio between the maximum possible power of a signal and the power of corrupting noise that affects the fidelity of its representation. It is defined as Eq. (41), where $\mathbf{Y}^{\text{est}}$ is the estimated image from denoising algorithms and $\mathbf{Y}^{\text{true}}$ is the ground truth.

$$\text{PSNR}(\mathbf{Y}^{\text{est}}, \mathbf{Y}^{\text{true}}) = 20\log\frac{\max(\mathbf{Y}^{\text{true}})}{\sqrt{\frac{1}{L}\sum_{p=1}^{L}(\mathbf{Y}_p^{\text{est}} - \mathbf{Y}_p^{\text{true}})^2}} \tag{41}$$

The structure similarity [23] (SSIM) is a perception-based model that considers image degradation as perceived change in structural information, while also incorporating important perceptual phenomena, including both luminance masking and contrast masking terms. Compared to PSNR, it is supposed to give more information about image distortion by the computation of local image structure, luminance and contrast into a single local quality score. In this paper, the luminance and contrast are normalized and SSIM is defined as Eq. (42),

$$\text{SSIM}(\mathbf{Y}^{\text{est}}, \mathbf{Y}^{\text{true}}) = \frac{2\mu_{\mathbf{Y}^{\text{est}}}\mu_{\mathbf{Y}^{\text{true}}} + C_1}{\mu^2_{\mathbf{Y}^{\text{est}}} + \mu^2_{\mathbf{Y}^{\text{true}}} + C_1} \cdot \frac{2\sigma_{\mathbf{Y}^{\text{est}}\mathbf{Y}^{\text{true}}} + C_2}{\sigma^2_{\mathbf{Y}^{\text{est}}} + \sigma^2_{\mathbf{Y}^{\text{true}}} + C_2} \tag{42}$$

where $\mathbf{Y}^{\text{est}}$ is the estimated image from denoising algorithms, $\mathbf{Y}^{\text{true}}$ is the ground truth, $\mu_{\mathbf{Y}^{\text{est}}}$, $\mu_{\mathbf{Y}^{\text{true}}}$, $\sigma_{\mathbf{Y}^{\text{est}}}$, $\sigma_{\mathbf{Y}^{\text{true}}}$ and $\sigma_{\mathbf{Y}^{\text{est}}\mathbf{Y}^{\text{true}}}$ are the local means, standard deviations and cross-covariance for images $\mathbf{Y}^{\text{est}}$ and $\mathbf{Y}^{\text{true}}$, $C_1$ and $C_2$ are the regularization constants to avoid instability for image regions where the local mean or standard deviation is close to zero.

In simulation, all the RMSE, PSNR and SSIM data are presented as box-and-whisker plots (center line, median; limits, 75% and 25% whiskers, maximum and minimum) along with all the data points. We use the paired one-way analysis of variation to do the multiple comparisons of these accuracy metrics. All the statistical tests are implemented with Prism 9 (GraphPad Software Inc.). Statistical significance at $P$<0.05,

0.01, 0.001 and 0.0001 are denoted by *, **, *** and ****, respectively. "ns" means "no significance".

## 3.3 Hot pixel removal methods evaluation

### 3.3.1 Optimal iteration number selection and running time evaluation

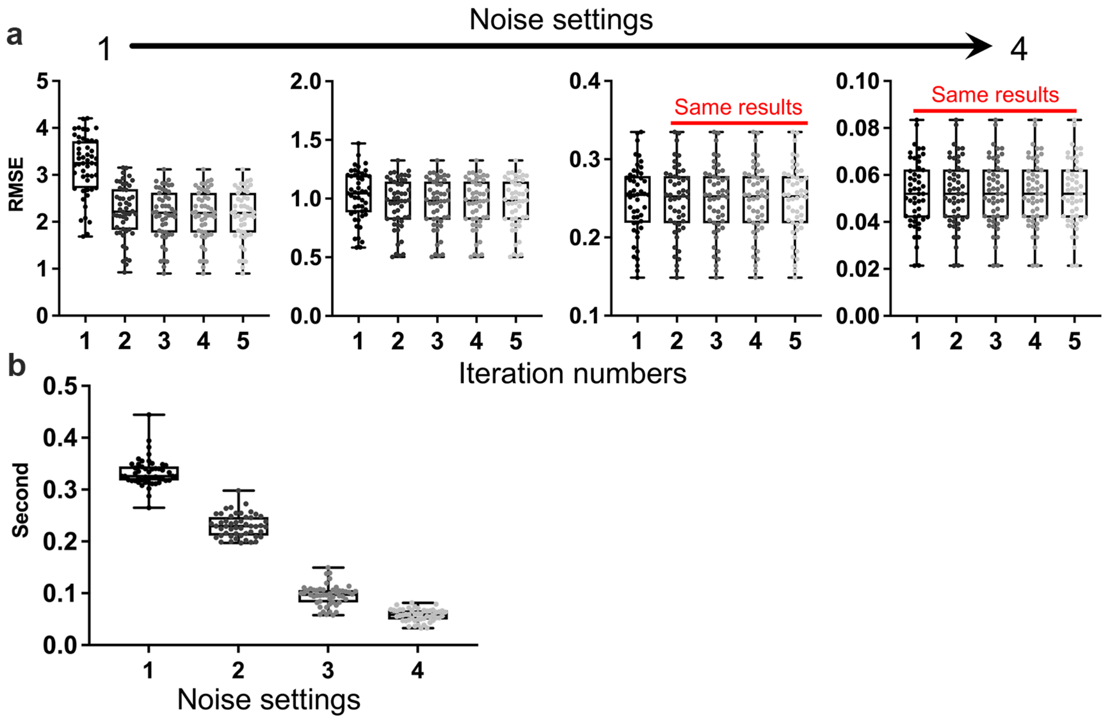


**Supplementary Figure 2.** DIMR algorithm evaluations on the simulated DNA dataset. (a) Evaluation of DIMR with $N_{\text{iter}}$ from 1 to 5 ($n = 50$ independent images per noise setting). "Same results" indicate the iteration stops beforehand. For instance, in noise setting 3, DIMR stops after the second iteration even though $N_{\text{iter}}$ is larger than 2. (b) DIMR running time evaluations under different noise settings with $N_{\text{iter}} = 3$ ($n = 50$ independent images per noise setting). In both (a) and (b), box center indicates median, box edges 25th and 75th percentile, and whiskers minimum and maximum percentile.

First, we evaluated the impact of $N_{\text{iter}}$ to the hot pixel removal results in DIMR. To achieve this, we utilized the simulated DNA dataset with 4 hot pixel conditions, in which each condition contains 50 images (Supplementary Table 1). $N_{\text{iter}}$ was set from 1 to 5. Besides, $l$ was empirically set as 4 as we consider half adjacent pixels should be close to a center pixel in a $3\times3$ window [24,25]. RMSE was utilized to evaluate the results, which is listed in Supplementary Fig. 2a. From the results, the accuracy almost does not improve after 3 iterations in all the noise settings. In particular, the DIMR algorithm stops the iteration before it reaches to the preset iteration times in low hot pixel density conditions (noise settings 3 and 4). Therefore, we recommend $N_{\text{iter}}$ as 3 to adequately remove hot pixels. We also evaluate the running time of DIMR under different hot pixels conditions with $N_{\text{iter}} = 3$ (Supplementary Fig. 2b). The results indicate

that it takes from approximately 0.05s to 0.4s for DIMR to remove hot pixels, depending on the hot pixel densities.

### 3.3.2 Benchmark DIMR with other hot pixel removal methods

Subsequently, we benchmarked our DIMR algorithm with NTHM and MTHM on the simulated CD14, Kerain and DNA datasets. Each marker contains 4 hot pixel conditions and each condition contains 50 images. We set the hyperparameters of DIMR, $l$ and $N_{\text{iter}}$, as 4 and 3 for all the datasets. The hyperparameters of NTHM and MTHM were optimized for each noise setting to guarantee their best performance.

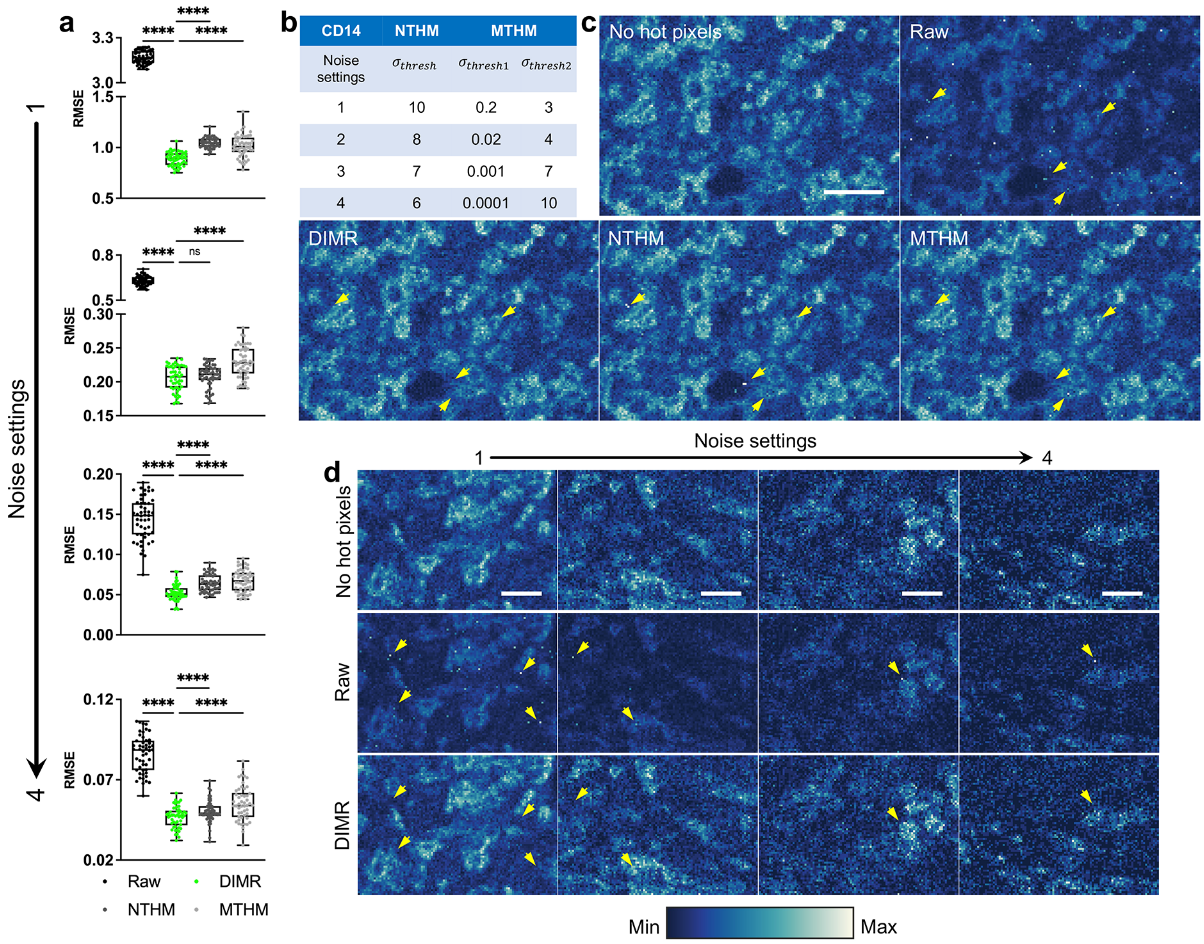


**Supplementary Figure 3.** Evaluation of the hot pixel removal methods on the simulated CD14 dataset. (a) RMSE comparison under different noise settings ($n = 50$ independent images per noise setting). Box center indicates median, box edges 25th and 75th percentile, and whiskers minimum and maximum percentile. $P$ values were calculated through two-sided Wilcoxon matched-paired test (****$P$<0.0001 and ns: no significance). (b) The optimal hyperparameters of NTHM and MTHM under different noise settings. (c) Visual inspection of the three hot pixel removal methods on a CD14 image with the highest hot pixel density under noise setting 1. (d) Visual inspection of the DIMR algorithm under different noise settings. Scale bar: (c) 20 $\mu$m, (d) 20 $\mu$m.

The evaluation results for these three markers are listed in Supplementary Figs. 3, 4 and 5, respectively.

As these figures suggest, DIMR always removes hot pixels effectively and outperforms NTHM and MTHM on RMSE under different hot pixel conditions. Besides, the optimal hyperparameters of NTHM and MTHM vary in a wide range with different settings of hot pixels (Supplementary Figs. 3b, 4b and 5b), while those of DIMR remain the same ($l = 4$ and $N_{\text{iter}} = 3$). In fact, the hot pixel conditions vary under different images and markers. Therefore, it is labor-intensive to tune the hyperparameters for every image. In comparison, the outlier detection of DIMR is based on the overall statistical features of the images, so that it is not essential to tune its hyperparameters. To summarize, the DIMR algorithm is more accurate and flexible for removing hot pixels in IMC images.

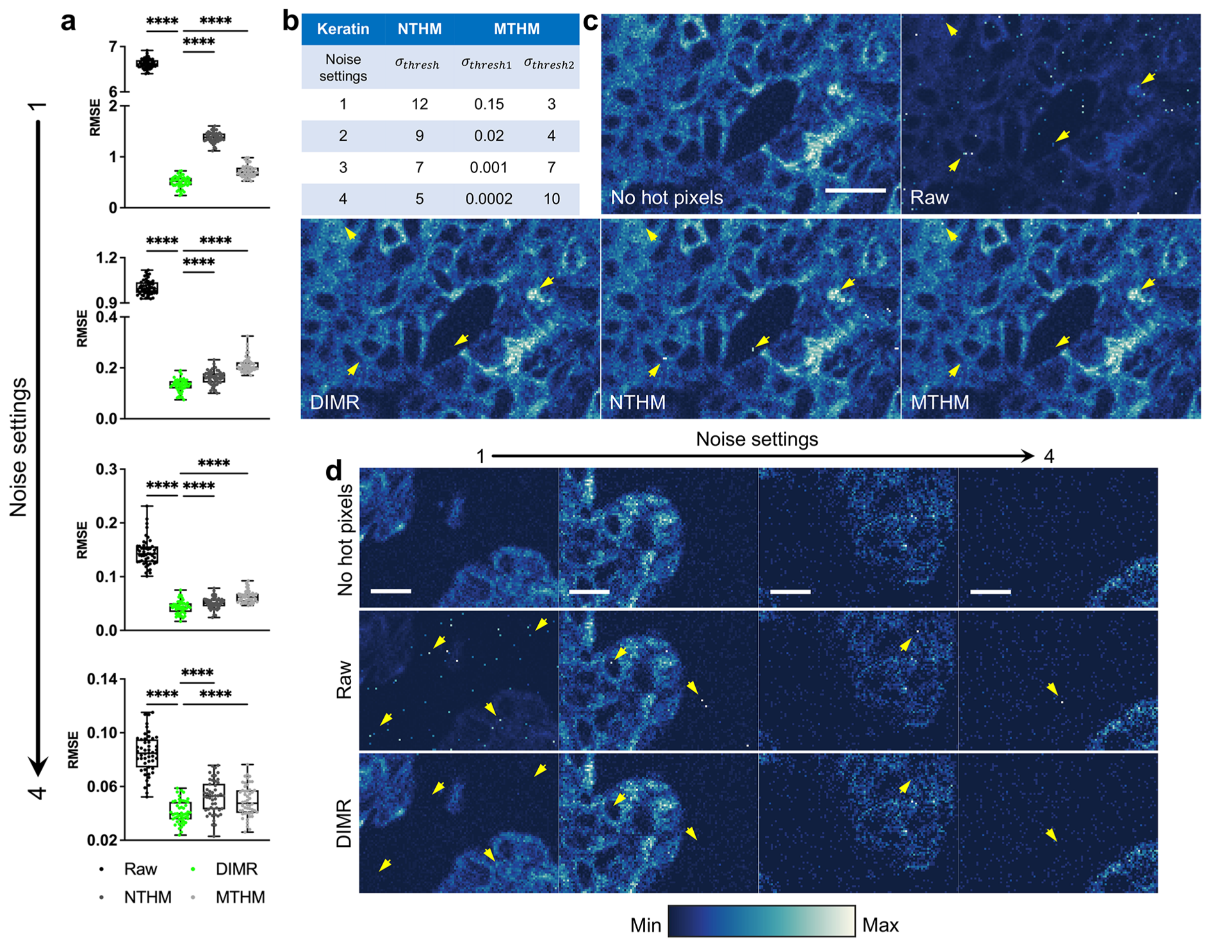


**Supplementary Figure 4.** Evaluation of the hot pixel removal methods on the simulated Keratin dataset. (a) RMSE comparison under different noise settings ($n = 50$ independent images per noise setting). Box center indicates median, box edges 25th and 75th percentile, and whiskers minimum and maximum percentile. $P$ values were calculated through two-sided Wilcoxon matched-paired test (****$P$<0.0001). (b) The optimal hyperparameters of NTHM and MTHM under different noise settings. (c) Visual inspection of the three hot pixel removal methods on a Keratin image with the highest hot pixel density under noise setting 1. (d) Visual inspection of the DIMR algorithm under different noise settings. Scale bar: (c) 20 $\mu$m, (d) 20 $\mu$m.

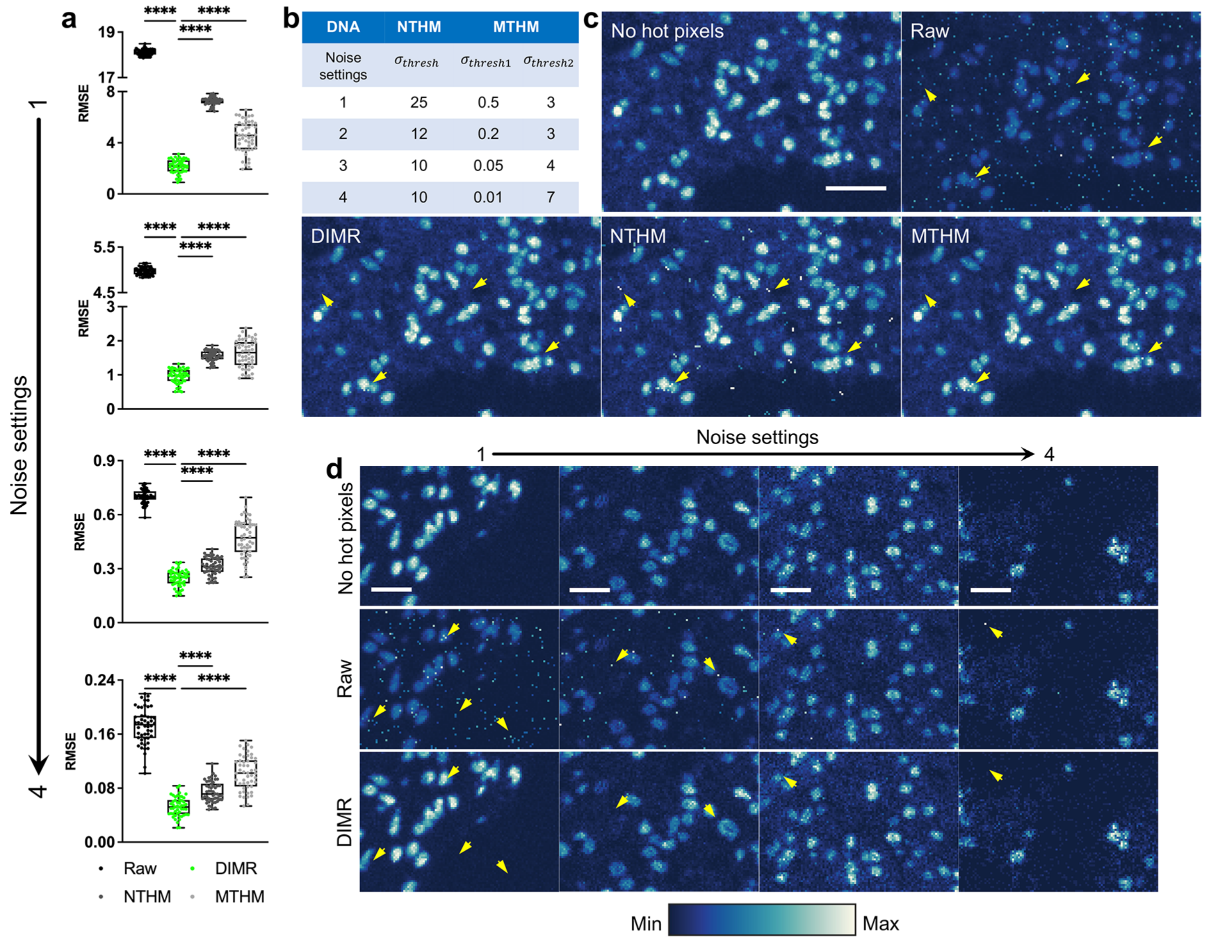


**Supplementary Figure 5.** Evaluation of the hot pixel removal methods on the simulated DNA dataset. (a) RMSE comparison under different noise settings ($n = 50$ independent images per noise setting). Box center indicates median, box edges 25th and 75th percentile, and whiskers minimum and maximum percentile. *P* values were calculated through two-sided Wilcoxon matched-paired test (****$P$<0.0001). (b) The optimal hyperparameters of NTHM and MTHM under different noise settings. (c) Visual inspection of the three hot pixel removal methods on a DNA image with the highest hot pixel density under noise setting 1. (d) Visual inspection of the DIMR algorithm under different noise settings. Scale bar: (c) 20 $\mu$m, (d) 20 $\mu$m.

## 3.4 Shot noise filtering methods evaluation

### 3.4.1 Compare DeepSNiF with Noise2Void and modified Noise2Void

We have also compared our DeepSNiF algorithm to Noise2Void (N2V), modified Noise2Void (MN2V) and Noise2True (N2T) on the simulated CD14 and Keratin images under 4 different noise levels. For each condition, we generated training sets and trained separate networks with 25 images for DeepSNiF, N2V, MN2V and N2T (Supplementary Table 6), and restored the other 25 images with the trained denoisers. Note that here DeepSNiF with no regularization (DeepSNiF-NR) was applied because we aimed to compare the performances of MSE and Eq. (23) as loss functions in the IMC denoising task. The denoised images were evaluated both visually and quantitatively, shown in Supplementary Figs. 6 and 7.

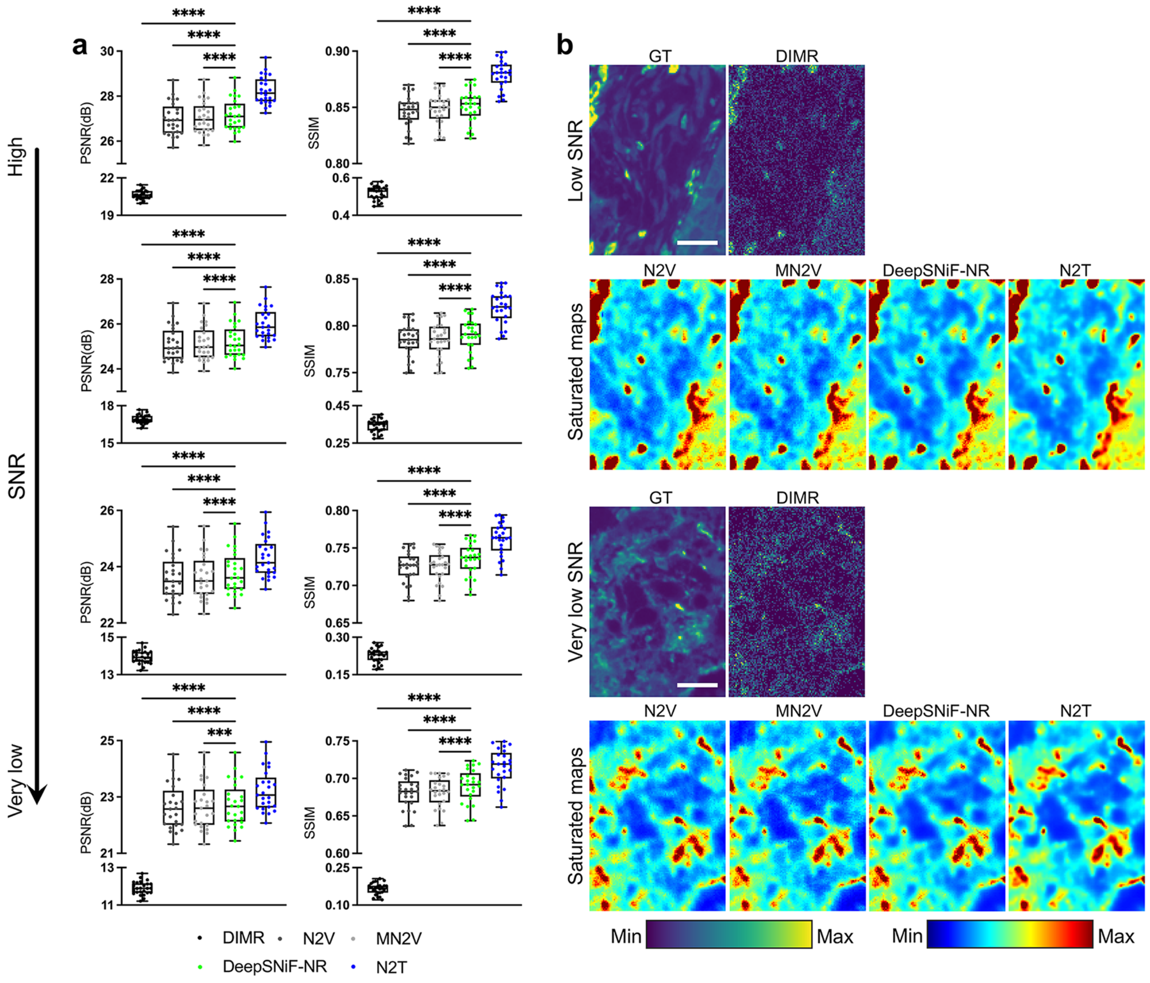


**Supplementary Figure 6.** Comparison of DeepSNiF-NR with N2V and MN2V on the simulated CD14 dataset with 4 noise levels. (a) PSNR and SSIM evaluation on the three algorithms ($n$ = 25 independent images per noise level). Box center indicates median, box edges 25th and 75th percentile, and whiskers minimum and maximum percentile. $P$ values were calculated through two-sided Wilcoxon matched-paired test (***$P$<0.001 and ****$P$<0.0001). (b) Visual inspection of the three algorithms and N2T on denoising a low SNR image. (c) Visual inspection of the three algorithms and N2T on denoising a very low SNR image. Scale bar: (b) 20 $\mu$m, (c) 20 $\mu$m.

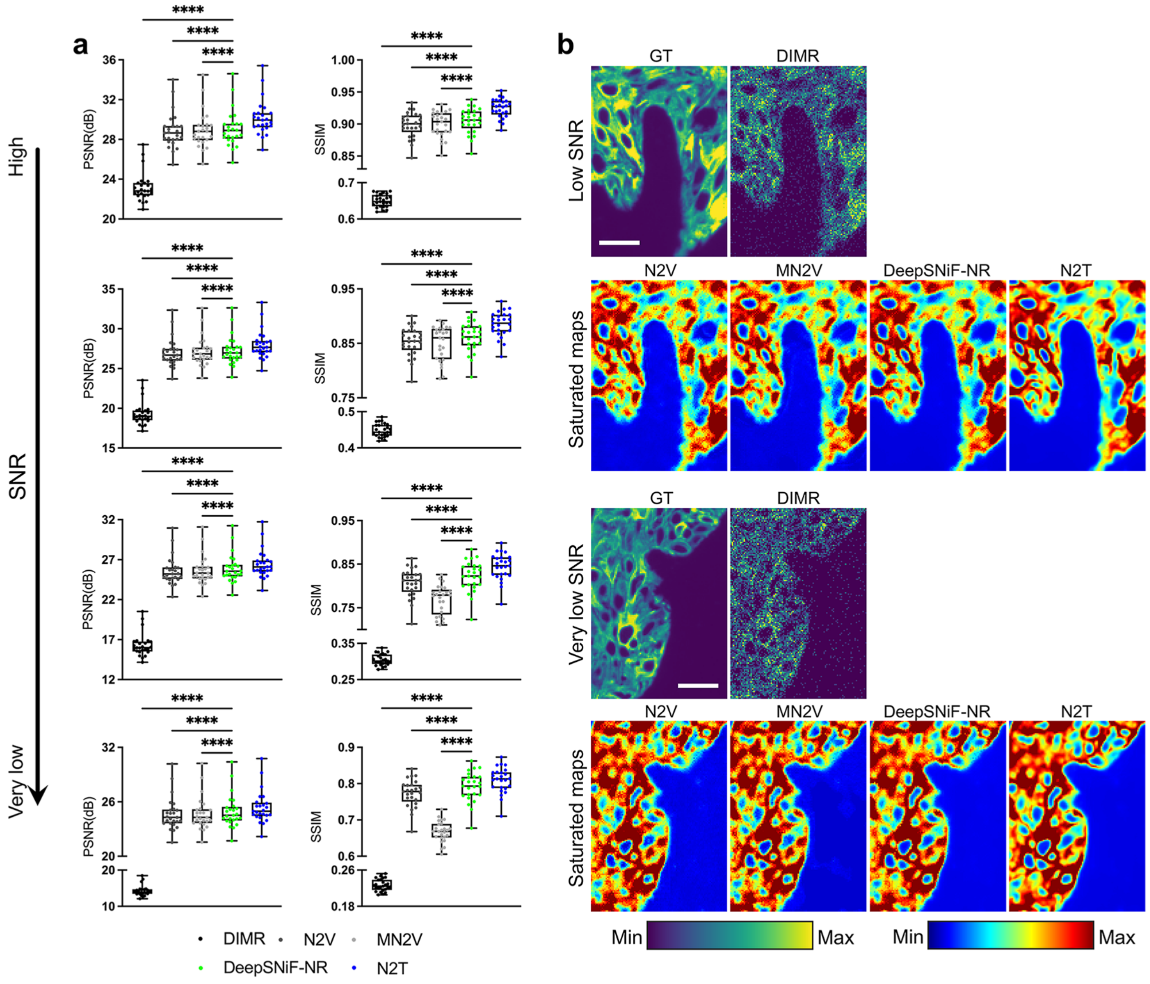


**Supplementary Figure 7.** Comparison of DeepSNiF-NR with N2V and MN2V on the simulated Keratin dataset with 4 noise levels. (a) PSNR and SSIM evaluation on the three algorithms ($n$ = 25 independent images per noise level). Box center indicates median, box edges 25th and 75th percentile, and whiskers minimum and maximum percentile. $P$ values were calculated through two-sided Wilcoxon matched-paired test (****$P$<0.0001). (b) Visual inspection of the three algorithms and N2T on denoising a low SNR image. (c) Visual inspection of the three algorithms and N2T on denoising a very low SNR image. Scale bar: (b) 20 $\mu$m, (c) 20 $\mu$m.

In both Supplementary Figs. 6a and 7a, N2T is always the best performer because of the availability of ground truths in training. Nevertheless, in self-supervised learning algorithms, DeepSNiF-NR wins over N2V and MN2V on both PSNR and SSIM. The restored image qualities are also reflected in Figs. 6b, c and 7b, c, in which saturated maps are applied to better visualize the differences between the algorithms. The restored images of N2V and MN2V are noiser than the DeepSNiF ones, which is more noticeable on low intensity regions . As we analyzed, this is because MSE is a biased estimator for Poisson noise, even with the Anscombe transformation. Interestingly, we observe the SSIM of MN2V is lower than N2V in several Keratin datasets (medium, low and very low SNRs). We infer this results from the ReLU activation of the output layer, which may suffer from the dead neuron problem. In this case, some pixels with very low

intensities are always 0 and do not response the back propagation. The 0 value pixels destroy the overall structure and hence lower the SSIM. To conclude, we have verified that our derived I-divergence is a better estimator than MSE on Poisson denoising task, and our derived DeepSNiF framework is more capable to restore IMC images than N2V and MN2V.

### 3.4.2 The effect of Hessian norm regularization on DeepSNiF

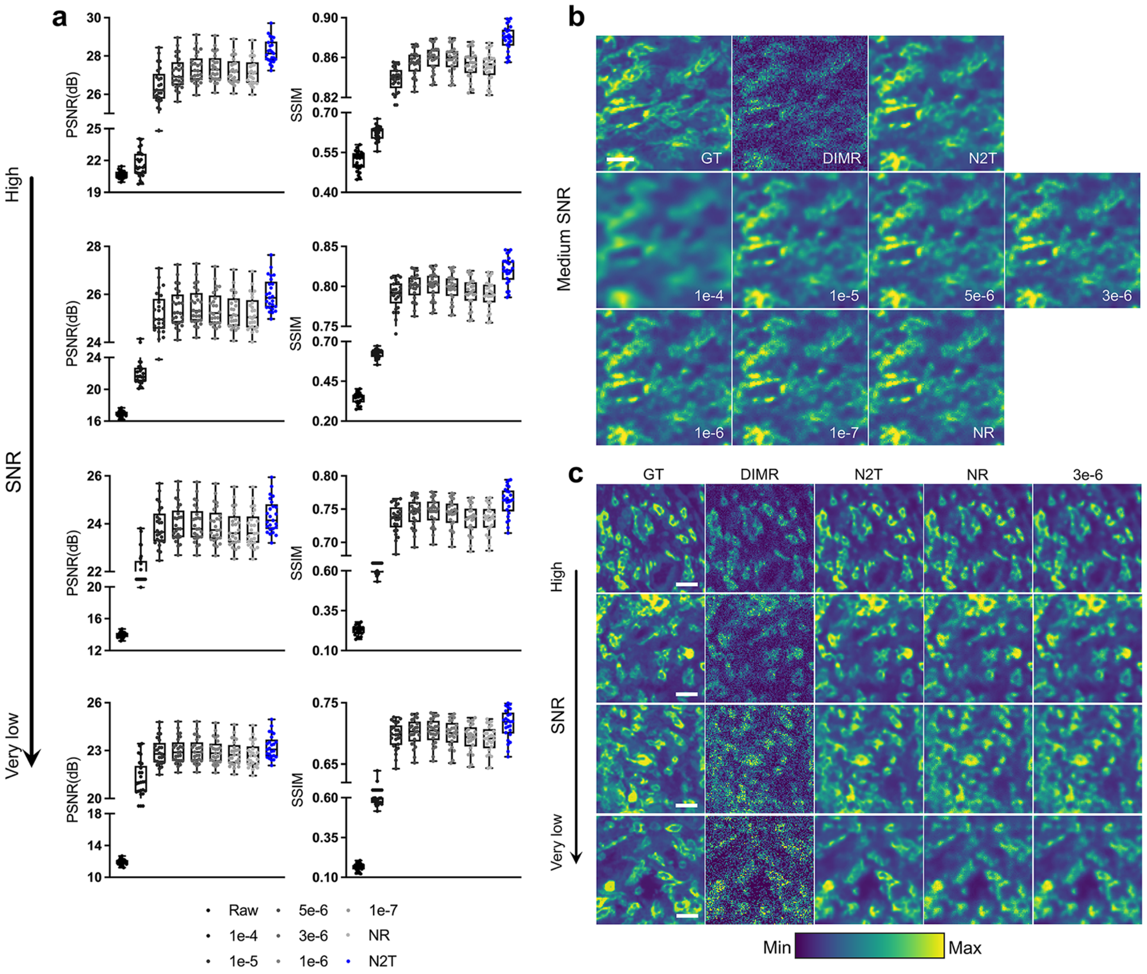


**Supplementary Figure 8.** The effect of Hessian norm regularization on the simulated CD14 image denoising. (a) PSNR and SSIM evaluation for DeepSNiF with different Hessian norm regularization parameters $\lambda_{\text{Hessian}}$ ($n = 25$ independent images per noise level). Box center indicates median, box edges 25th and 75th percentile, and whiskers minimum and maximum percentile. (b) Visual inspection of DeepSNiF with different Hessian norm regularization parameters $\lambda_{\text{Hessian}}$ on a simulated CD14 image with medium SNR. (c) Visual inspection of DeepSNiF when $\lambda_{\text{Hessian}} = 0$ (NR) and $\lambda_{\text{Hessian}} = 3\text{e-}6$ on simulated CD14 images with different noise levels. Scale bar: (b) 20 $\mu$m, (c) 20 $\mu$m.

Even though DeepSNiF-NR is superior to N2V and MN2V for the task of IMC denoising, it still suffers from discontinuous artifacts and there remains a big gap between the performance of DeepSNiF and N2T. To further enhance DeepSNiF, the Hessian norm regularization is applied in the loss function (31). In par-

ticular, $\lambda_{\text{Hessian}}$ determines the strength of regularization and thus further affects the denoising performance. To choose a proper $\lambda_{\text{Hessian}}$, we trained a series of networks with a range of $\lambda_{\text{Hessian}}$ from 1e-4 to 1e-7 and no regularization (NR) on the simulated CD14 and Keratin images. Here, 25 images were used for training and the other 25 for testing (in each dataset).

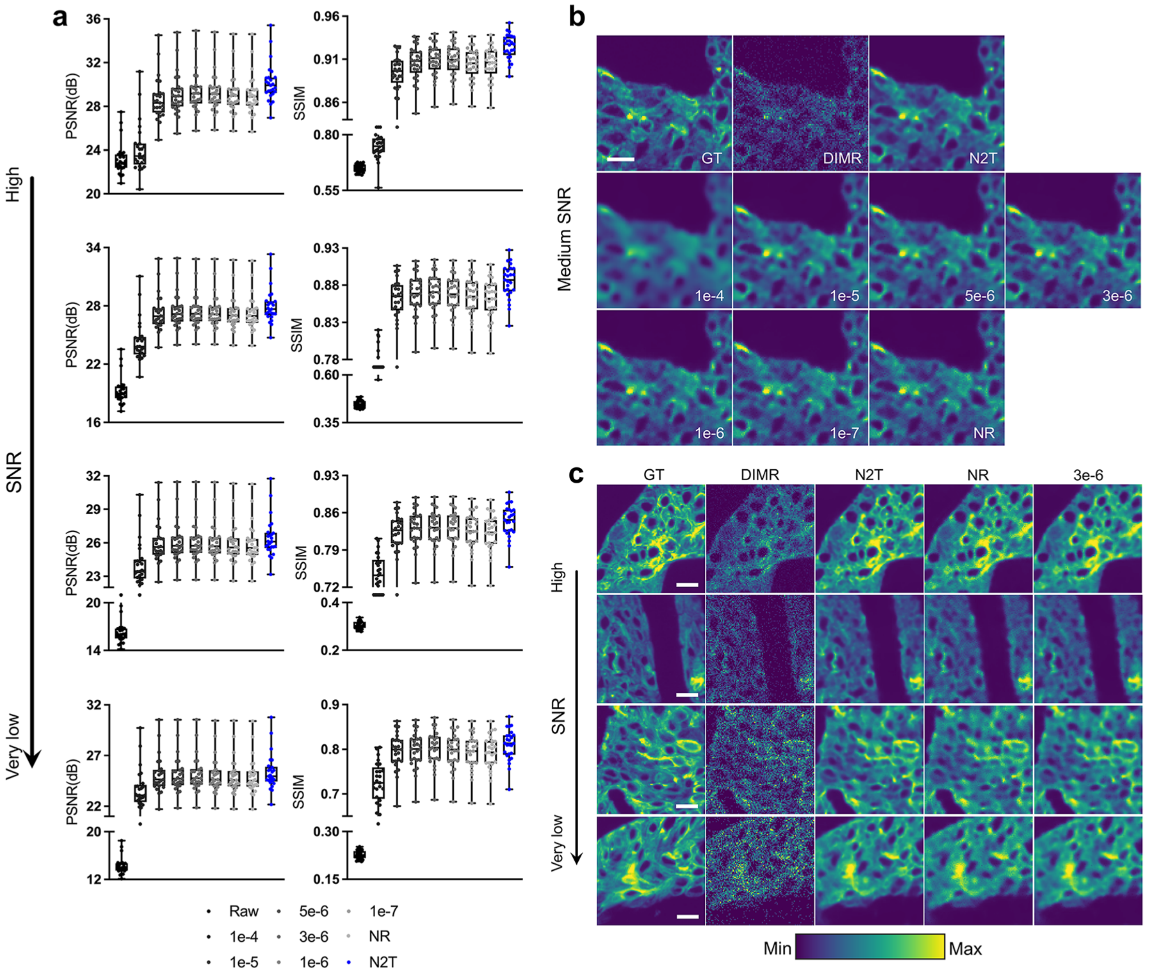


**Supplementary Figure 9.** The effect of Hessian norm regularization on the simulated Keratin image denoising. (a) PSNR and SSIM evaluation for DeepSNiF with different Hessian norm regularization parameters $\lambda_{\text{Hessian}}$ ($n = 25$ independent images per noise level). Box center indicates median, box edges 25th and 75th percentile, and whiskers minimum and maximum percentile. (b) Visual inspection of DeepSNiF with different Hessian norm regularization parameters $\lambda_{\text{Hessian}}$ on a simulated Keratin image with medium SNR. (c) Visual inspection of DeepSNiF when $\lambda_{\text{Hessian}} = 0$ (NR) and $\lambda_{\text{Hessian}}$=3e-6 on simulated Keratin images with different noise levels. Scale bar: (b) 20 $\mu$m, (c) 20 $\mu$m.

The quantitative and qualitative results are presented in Supplementary Figs. 8 and 9. The PSNR and SSIM from both groups (Supplementary Figs. 8a and 9a) indicate Hessian norm regularization could enhance the performance of DeepSNiF with a proper $\lambda_{\text{Hessian}}$. Specifically, we found DeepSNiF achieves its optimal performance when $\lambda_{\text{Hessian}}$ is approximately 3e-6. We then investigated the qualities of the restored images from the medium SNR datasets (Supplementary Figs. 8b and 9b). The larger $\lambda_{\text{Hessian}}$, the stronger

regularization will be. Therefore, the images look more blurry when $\lambda_{\text{Hessian}}$ = 1e-4 and noisier with no regularization. With $\lambda_{\text{Hessian}}$ = 3e-6, the restored images achieve their best qualities. Subsequently, by observing the denoised images from different noise levels (Supplementary Figs. 8c and 9c), the Hessian norm regularization effectively modifies the discontinuous artifacts in the images without regularization. We conclude that the Hessian norm regularization effectively improves the performance of DeepSNiF, both quantitatively and qualitatively, even boost the performance of DeepSNiF close to that of N2T. As a consequence, we set DeepSNiF with $\lambda_{\text{Hessian}}$ = 3e-6 for the subsequent experiments due to its optimal accuracy.

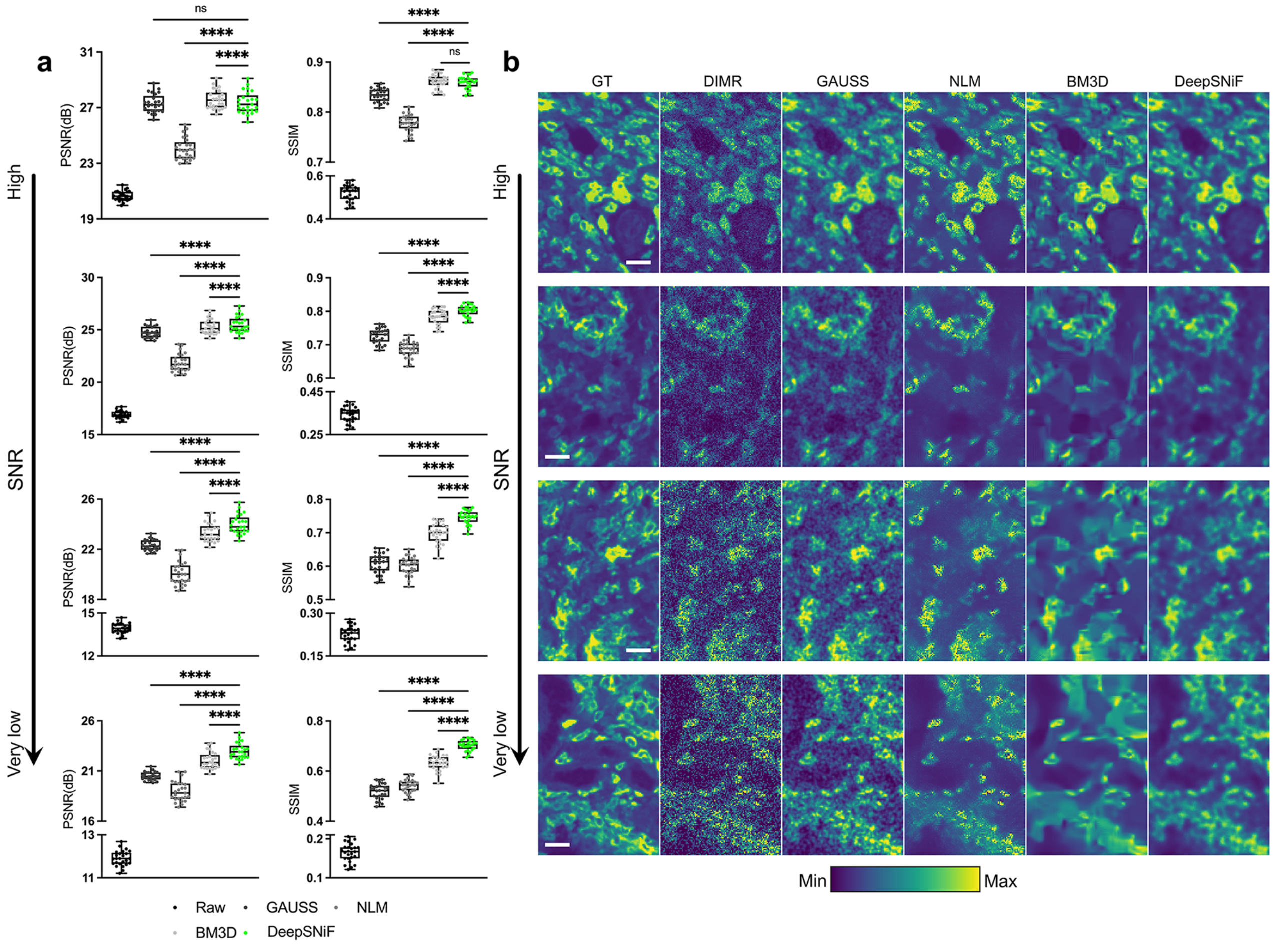


**Supplementary Figure 10.** Comparison of DeepSNiF with GAUSS, NLM and BM3D algorithms on the simulated CD14 dataset with 4 noise levels. (a) PSNR and SSIM evaluation on the algorithms ($n$ = 25 independent images per noise level). Box center indicates median, box edges 25th and 75th percentile, and whiskers minimum and maximum percentile. $P$ values were calculated through two-sided Wilcoxon matched-paired test (****$P$<0.0001 and ns: no significance). (b) Visual inspection of the algorithms on denoising the images with different noise levels. Scale bar: 20 $\mu$m.

### 3.4.3 Compare DeepSNiF with traditonal denoising methods

We are also interested in comparing DeepSNiF with traditional statistical denoising methods, including Gaussian filter (GAUSS), NLM and BM3D algorithms. These three algorithms along with DeepSNiF were benchmarked on the simulated CD14 and Keratin datasets. Notably, they were only applied on the 25 test images of DeepSNiF. The results are presented in Supplementary Figs. 10 and 11.

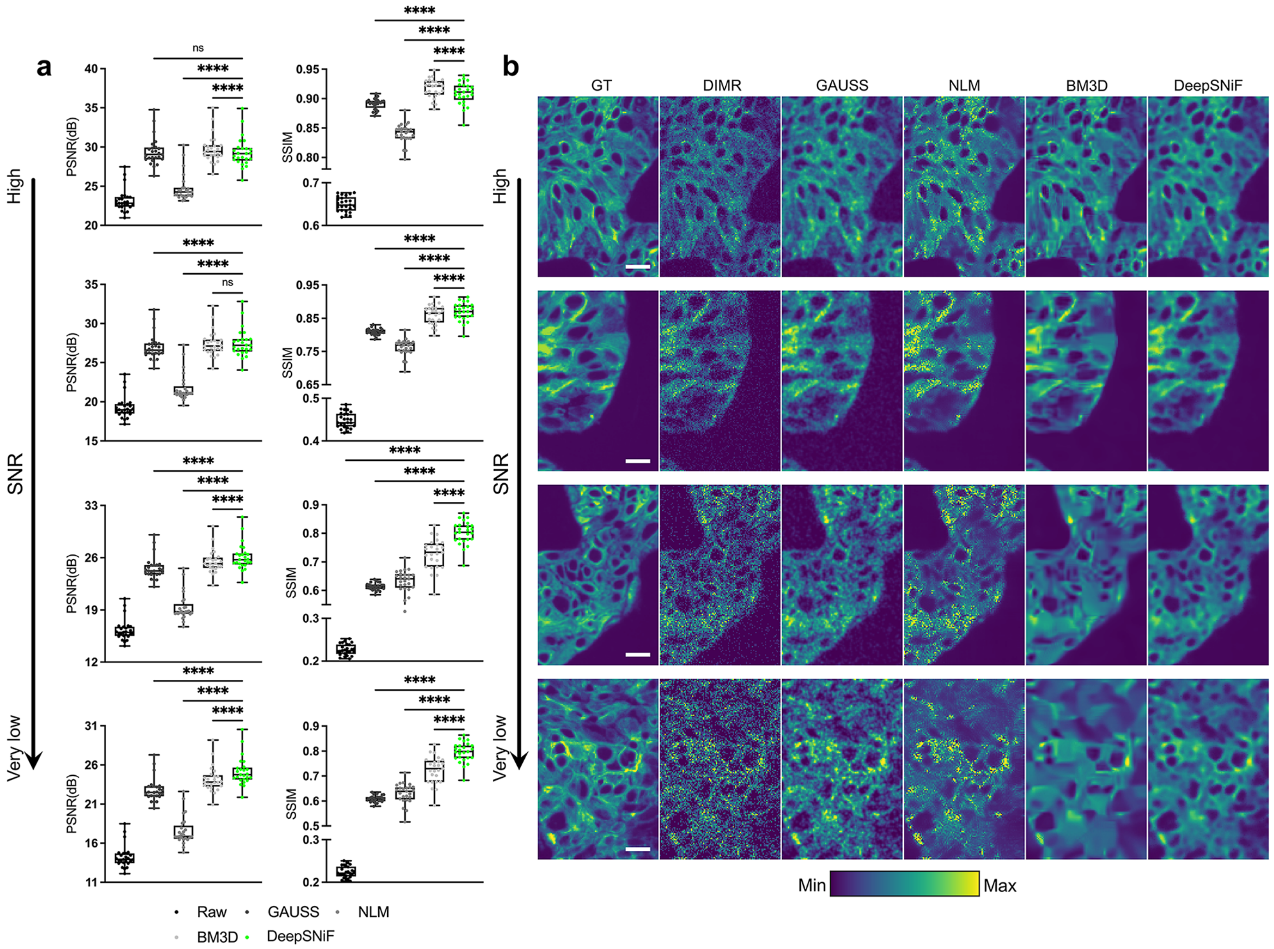


**Supplementary Figure 11.** Comparison of DeepSNiF with GAUSS, NLM and BM3D algorithms on the simulated Keratin dataset with 4 noise levels. (a) PSNR and SSIM evaluation on the algorithms ($n = 25$ independent images per noise level). Box center indicates median, box edges 25th and 75th percentile, and whiskers minimum and maximum percentile. $P$ values were calculated through two-sided Wilcoxon matched-paired test (****$P$<0.0001 and ns: no significance). (b) Visual inspection of the algorithms on denoising the images with different noise levels. Scale bar: 20 $\mu$m.

From these quantitative evaluations (Supplementary Figs. 10a and 11a), we can observe that DeepSNiF is frequently better than the other algorithms on PSNR and SSIM, except for high SNR images. This is also in accordance with the visual inspection (Supplementary Figs. 10b and 11b). For high SNR images, BM3D performs slightly better than DeepSNiF. Nevertheless, as SNR becomes lower, BM3D tends to over-smooth the restored images and details are lost. In fact, the SNR of IMC images are always low because of

the limited ion counts. As a result, BM3D is not fit for IMC denoising, even though its PSNR and SSIM are the closest to those of DeepSNiF. GAUSS and NLM are outperformed by BM3D, and therefore not of interest for IMC data processing. In particular, the GAUSS algorithm can be regarded as a low-pass filter and only the high frequency noises are filtered. Similar to BM3D, NLM algorithm also blurs the restored images. However, it still leaves noisy regions and its denoising ability is even weaker than GAUSS. To conclude, traditional statistical denoising algorithms are not suited to IMC denoising, and deep learning-based algorithms such as DeepSNiF may be preferred.

## Supplementary Note 4: Tutorial of IMC-Denoise software package

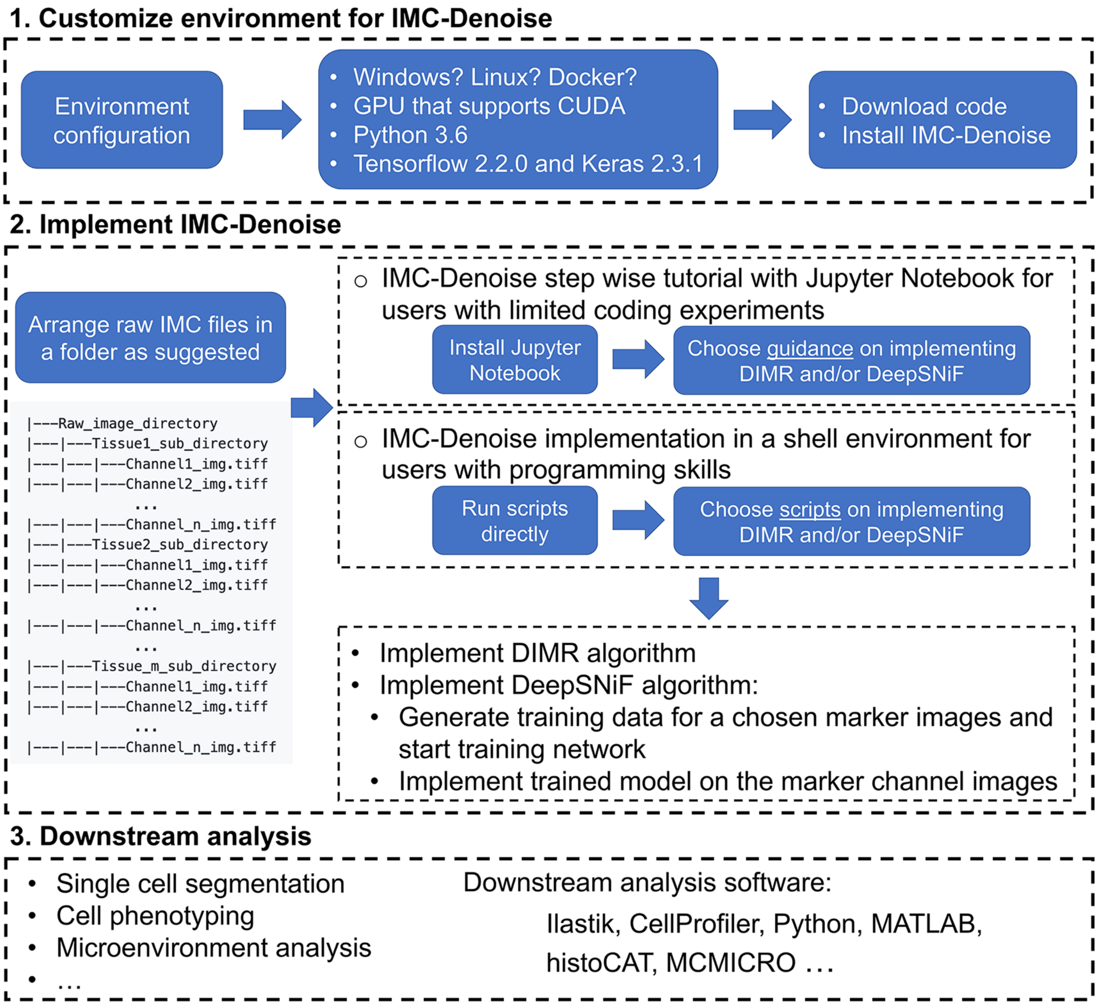


**Supplementary Figure 12.** A schematic diagram to implement IMC-Denoise.

To ameliorate the difficulty of using our IMC-Denoise software package, we have built a tutorial in our GitHub page `https://github.com/PENGLU-WashU/IMC_Denoise` to implement IMC-Denoise from installation to denoise their own data. Besides, Jupyter Notebook files are also included in this package

to explain the algorithm step by step. This package is easy to install and convenient to use. Researchers without expertise in computer science and machine learning can learn to use it in a very short time with our tutorial. We provide a short introduction to the tutorial as Supplementary Fig. 12.

Before implementing IMC-Denoise, the corresponding programming environment should be built. Our package can be run both on Windows and Linux system. A docker environment is also available. A GPU that supports CUDA is essential to train a DeepSNiF model. Besides, we implement the algorithm with Python 3.6, Tensorflow 2.2.0 and Keras 2.3.1. Once the environment is built, the IMC-Denoise software package can be downloaded and installed as instructed in our GitHub page.

The raw IMC image files should be stored in a folder as our suggestion (Supplementary Fig. 12) so that our code can read the files correctly. For the potential users with limited coding experiments, we provide step wise tutorials with Jupyter Notebook to implement DIMR and DeepSNiF. While for those users with programming experiences, running the algorithms with script commends is more flexible. Normally, we suggest the users run the algorithms with default hyperparameters – only the input and output folders should be changed. After denoising, the restored images could be fed into other softwares or customized scripts for further downstream analysis.

# Other Supplementary Figures

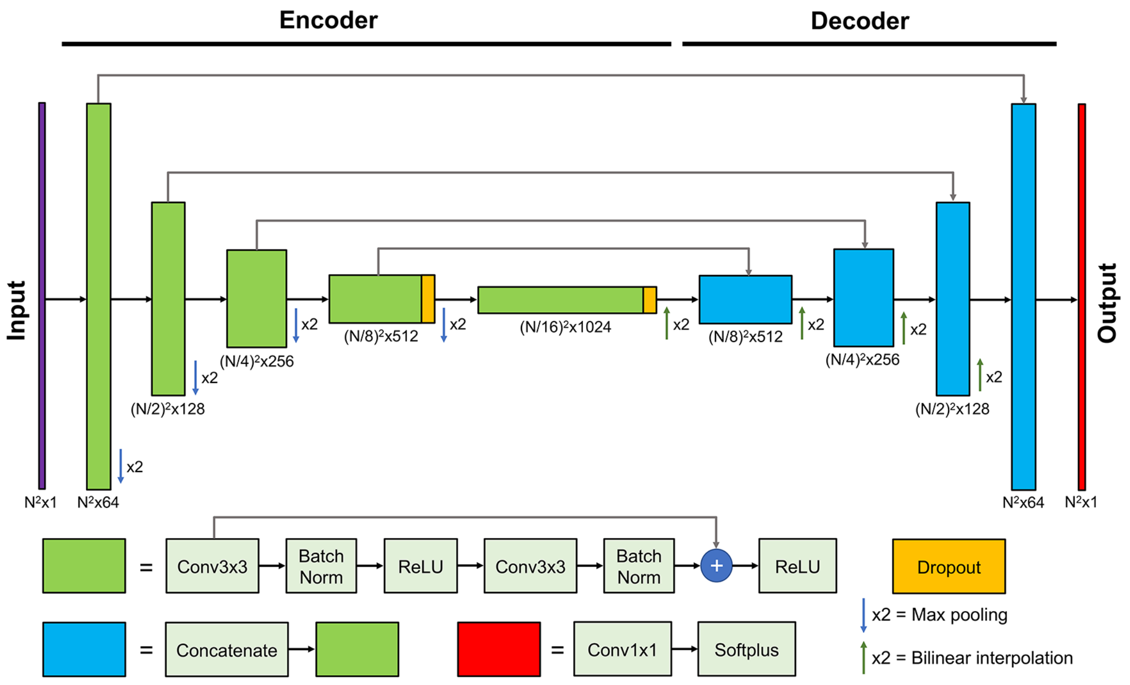


**Supplementary Figure 13.** The proposed convolutional neural network structure. The overall structure of the network follows the U-Net architecture modified with Res-block modules. The input and the output images share the same size. Starting with the noisy image, the encoder path gradually condenses the spatial information into high-level feature maps with growing depths (size marked by the number in the bottom of each block); the decoder path reverses the process by recombining the information into feature maps with gradually increased lateral details. The information in adjacent feature maps transfers by convolving with $3\times3$ convolutional filters. The down-sampling is done by $2\times2$ max-pooling operation and the up-sampling is done by $2\times2$ up-sampling operation. Res-blocks are applied to facilitate efficient training. Each Res-block contains a convolution layer (Conv), batch normalization (Batch Norm) and the rectified linear unit (ReLU) nonlinear activation. We also added dropout layers with 0.5 dropout rate after the central two res-blocks to mitigate overfitting. Skip connections are added to tunnel the high-frequency information from shallower layers to deeper layers with the same spatial scales. We use the softplus activation function $\log(1{+}\exp(x))$ in the final layer to restrict the final prediction to be non-negative.

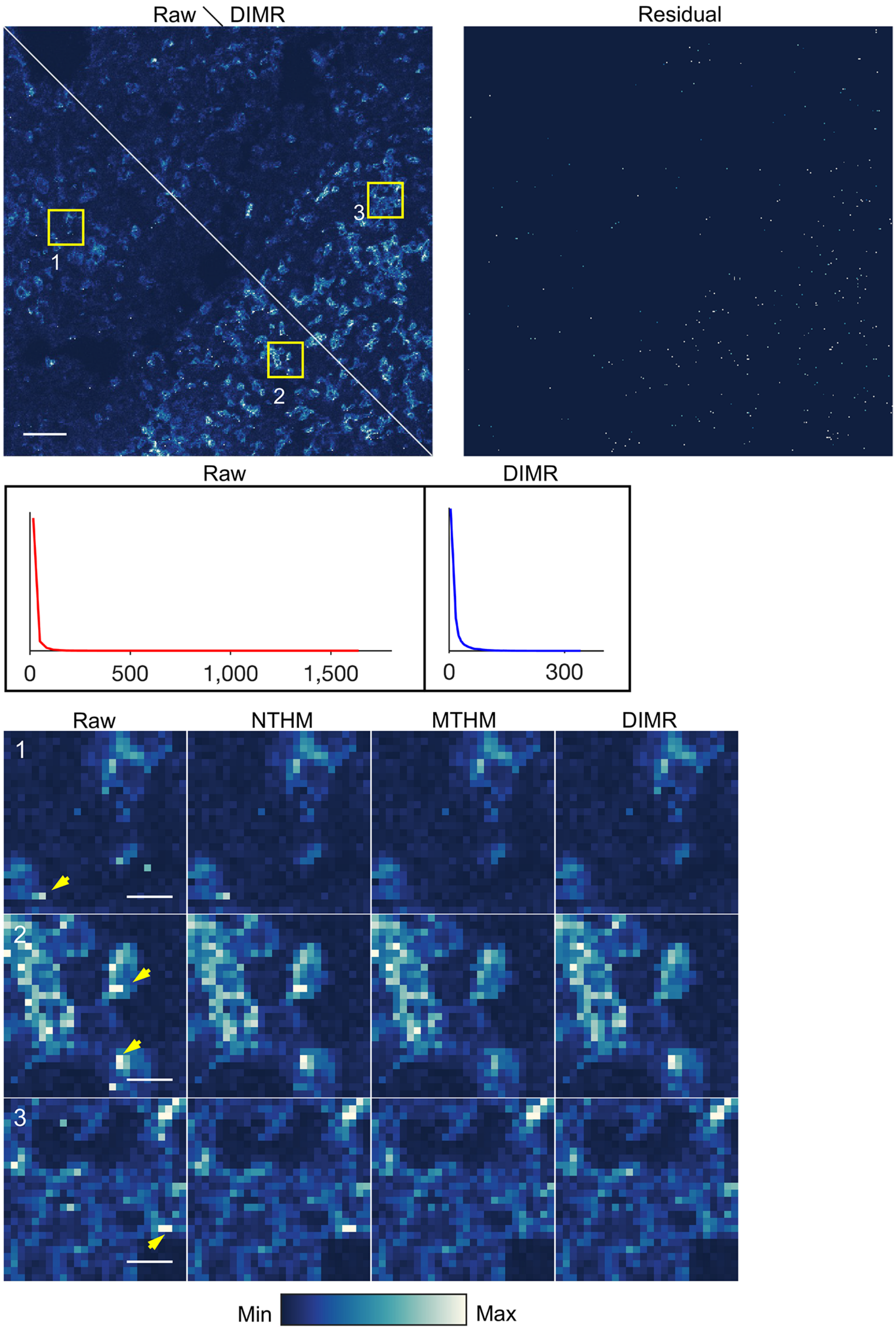


**Supplementary Figure 14.** DIMR algorithm enables adaptive hot pixel removal on CD235a stained channel in human bone marrow IMC. (a) Comparison of the raw and DIMR-processed images, where the lower left and upper rights parts correspond to the raw and DIMR-processed images, respectively. (b) Difference between the raw and DIMR-processed images, in which the residual pixels correspond to the detected hot pixels. (c) The corresponding histograms of raw and DIMR-processed images in (a). (d) Comparisons between the raw, NTHM, MTHM and DIMR-processed images. (d1)–(d3) correspond to the sub-regions labeled from 1 to 3 in (a). Scale bar: (a) 40 $\mu$m, (d) 6 $\mu$m.

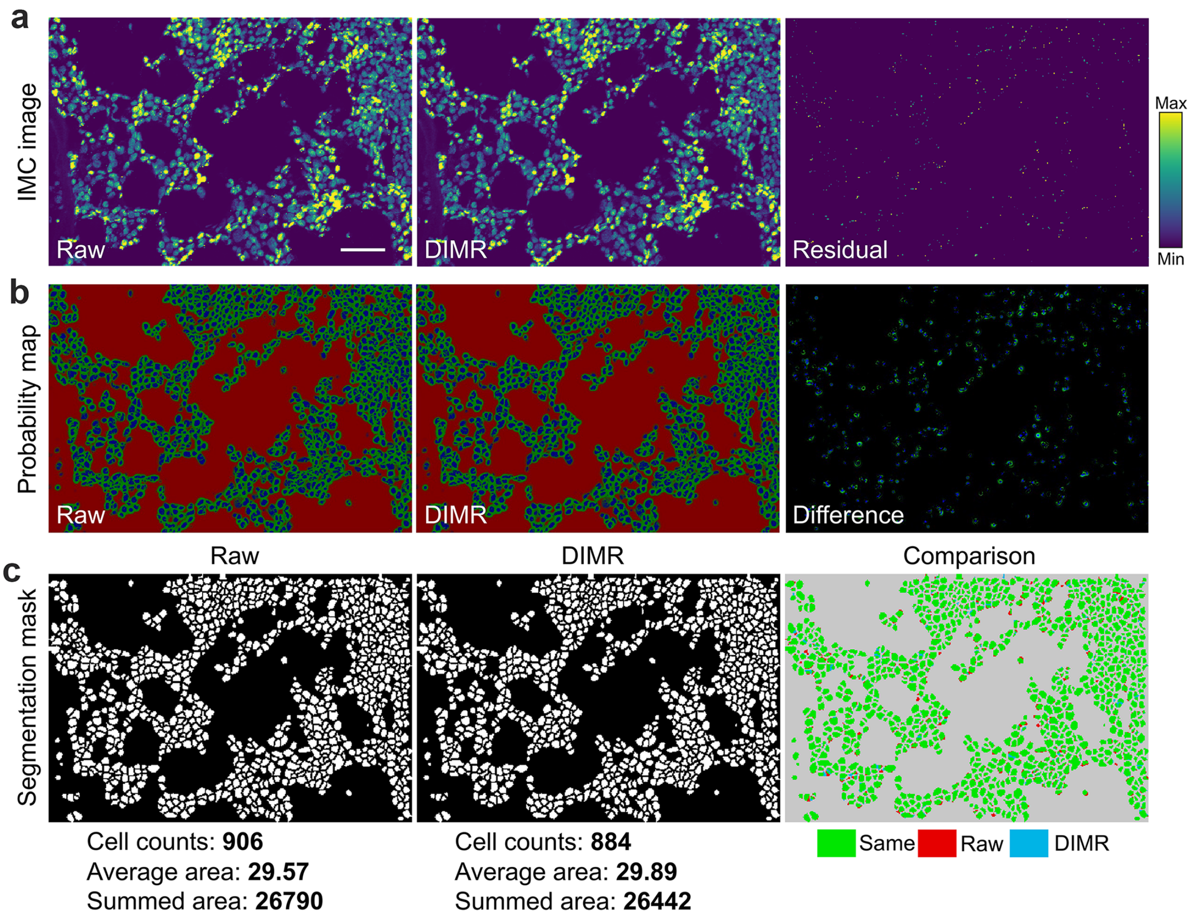


**Supplementary Figure 15.** DIMR slightly enhances single cell segmentation. (a) Comparison between the raw and DIMR-processed DNA images. (b) The probability maps of the raw and DIMR-processed images generated by Ilastik software, and their difference map (blue: nuclei, green: cytoplasm, and red:background). (c) The segmentation masks of the raw and DIMR-processed images generated by Cell Profiler software, and their overlaid comparison map. To account for the impact of hot pixels in the raw image, the segmented masks with areas smaller than 5 have been removed in both segmentation masks. By comparing the figures in the third column, the different segmented masks between raw and DIMR images are frequently caused by the presence of hot pixels. Even with a cell size threshold, the hot pixels can still split cells, and falsely expand normal cell borders. Correspondingly, the raw image segmented a little more cells than that of DIMR (906 to 994). At the same time, the average area of cells from the raw image is a little smaller (29.57 to 29.89), but the summed area is slightly larger (26790 to 26442). Scale bar: 47 $\mu$m.

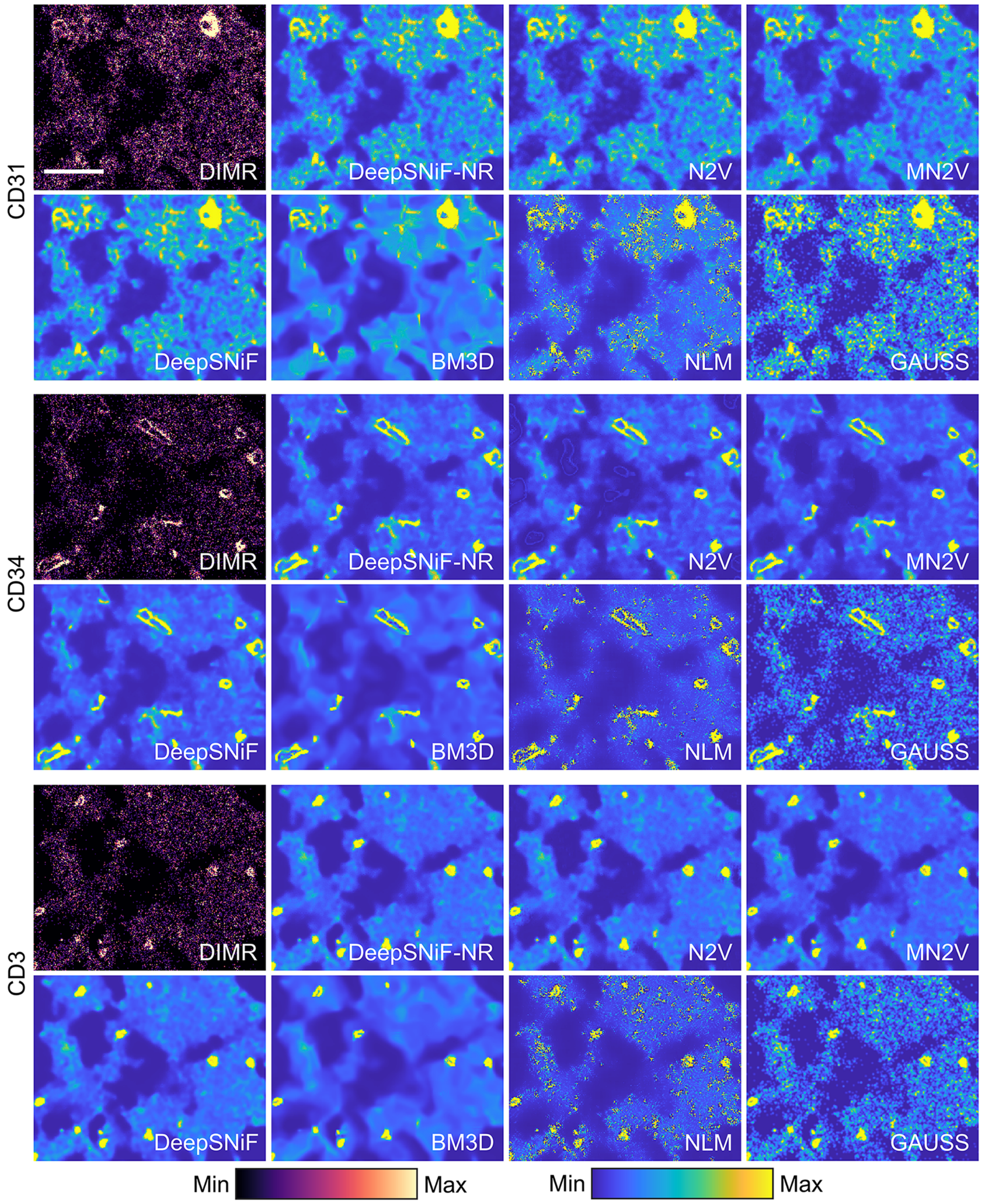


**Supplementary Figure 16.** Visual inspection of DeepSNiF and other statistics-based denoising algorithms on denoising (a) CD31, (b) CD34, (c) CD3-labeled IMC images. Scale bar: 60 $\mu$m.

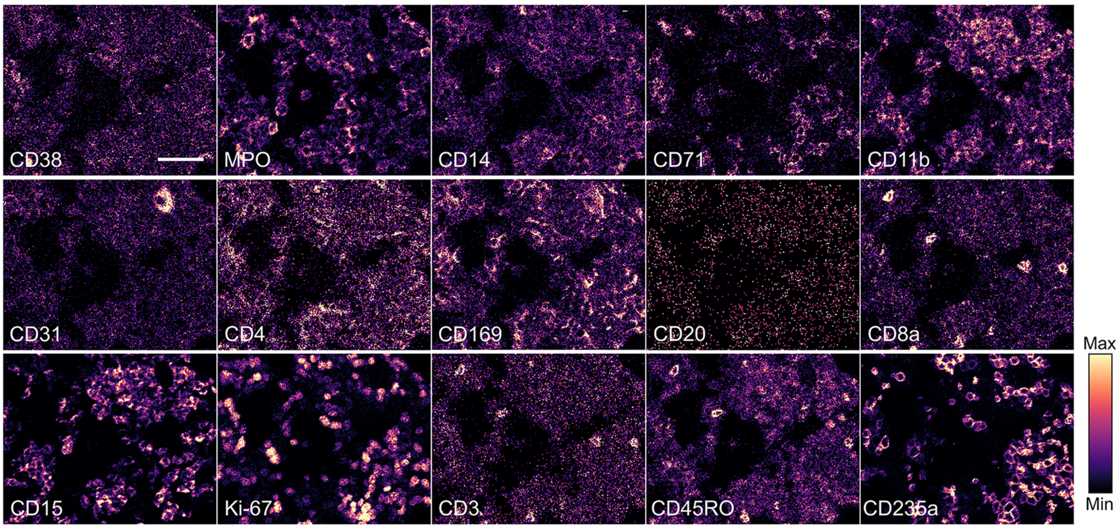


**Supplementary Figure 17.** The raw IMC images corresponding to Fig. 1f. Scale bar: 48 $\mu$m.

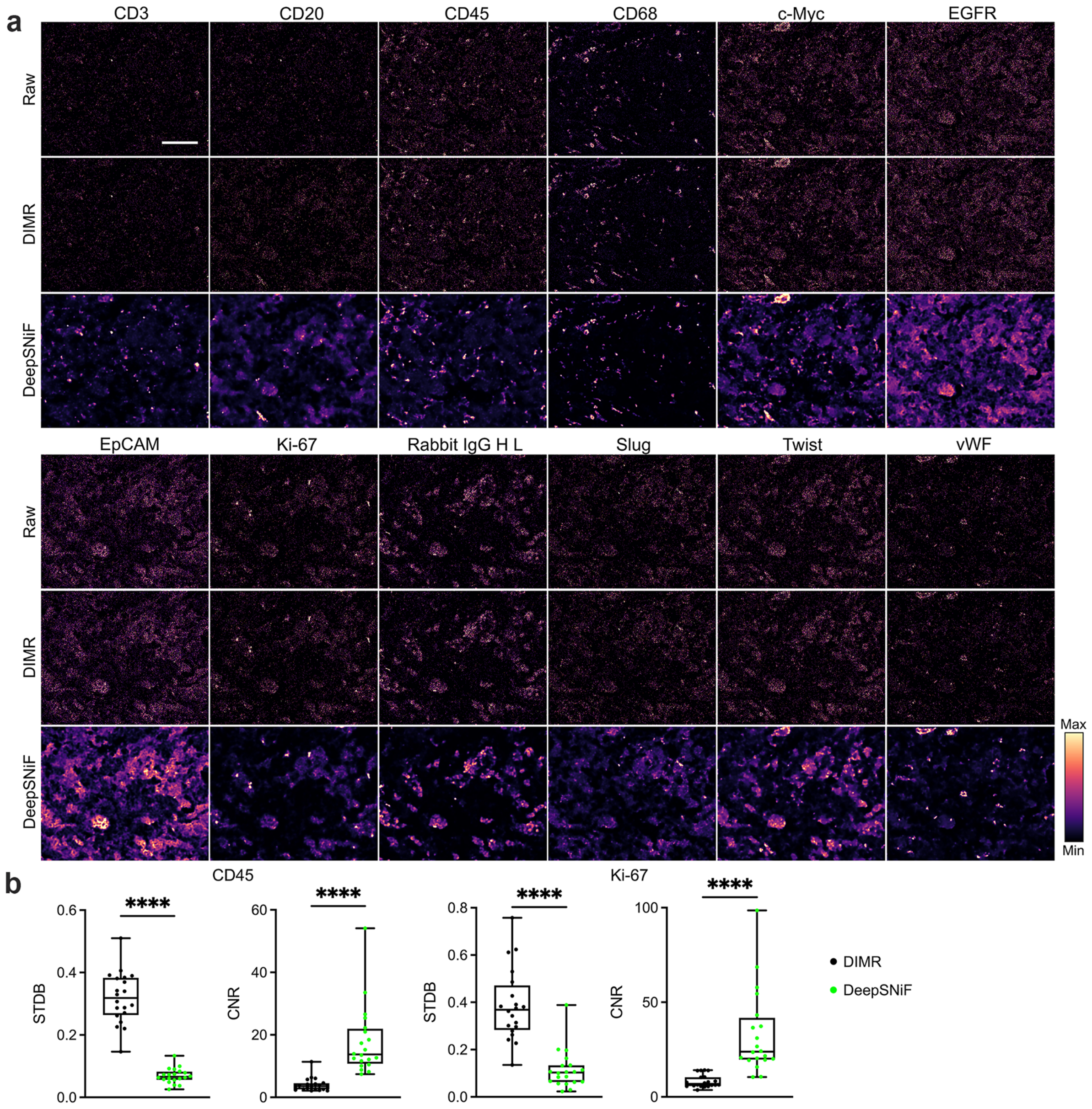


**Supplementary Figure 18.** IMC-Denoise enhances the human breast cancer IMC dataset. (a) Visual inspection of DIMR and DeepSNiF denoised IMC images labeled with different antibodies. (b) DeepSNiF significantly improves image qualities on denoising CD45 and Ki-67-labeled IMC images in terms of STDB and CNR ($n = 20$ independent images for each marker). Box center indicates median, box edges 25th and 75th percentile, and whiskers minimum and maximum percentile. $P$ values were calculated through two-sided Wilcoxon matched-paired test (****$P$<0.0001). Scale bar: 100 $\mu$m.

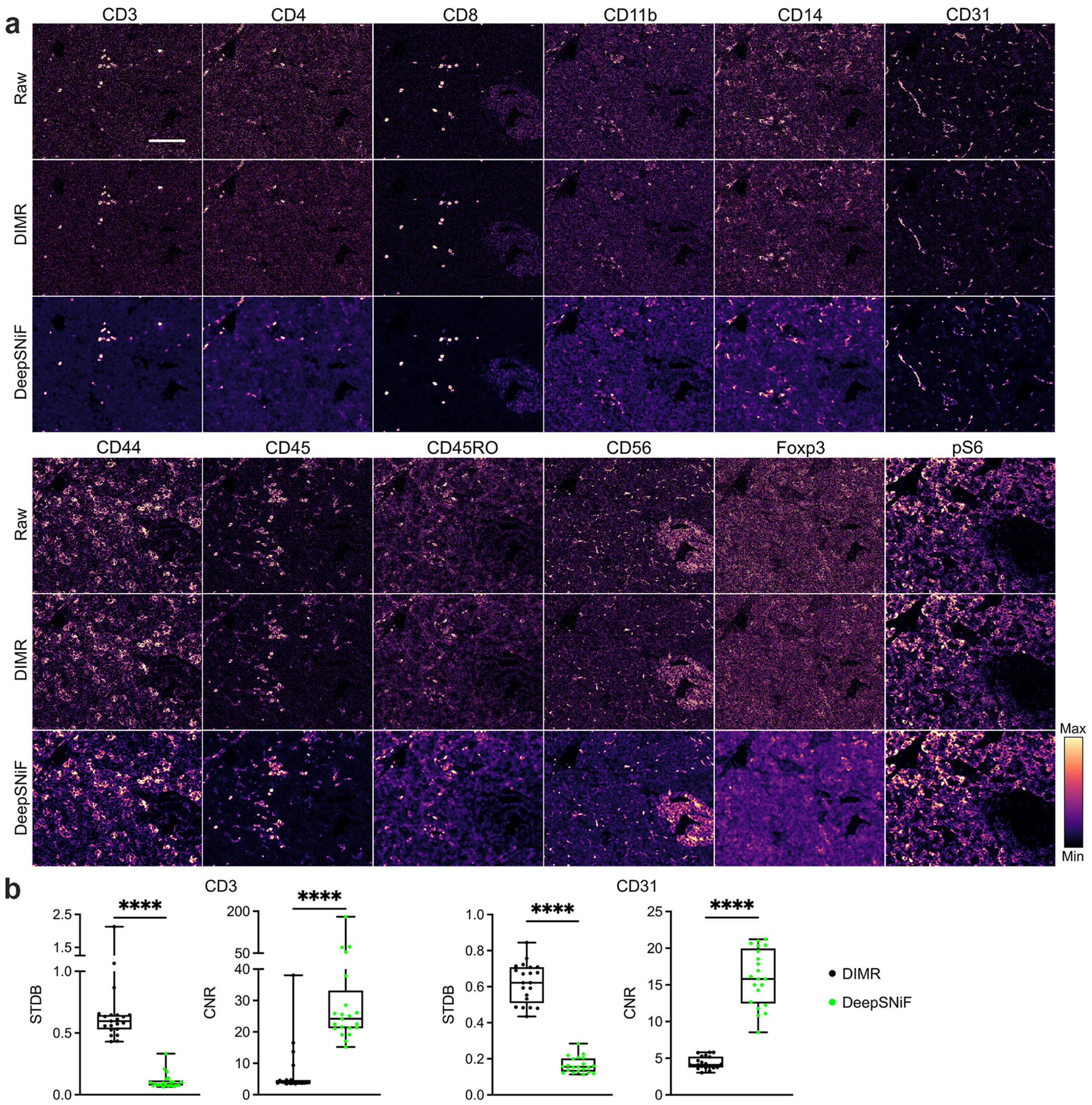


**Supplementary Figure 19.** IMC-Denoise enhances the human pancreatic cancer IMC dataset. (a) Visual inspection of DIMR and DeepSNiF denoised IMC images labeled with different antibodies. (b) DeepSNiF significantly improves image qualities on denoising CD3 and CD31-labeled IMC images in terms of STDB and CNR ($n = 21$ independent images for each marker). Box center indicates median, box edges 25th and 75th percentile, and whiskers minimum and maximum percentile. $P$ values were calculated through two-sided Wilcoxon matched-paired test (****$P$<0.0001). Scale bar: 100 $\mu$m.

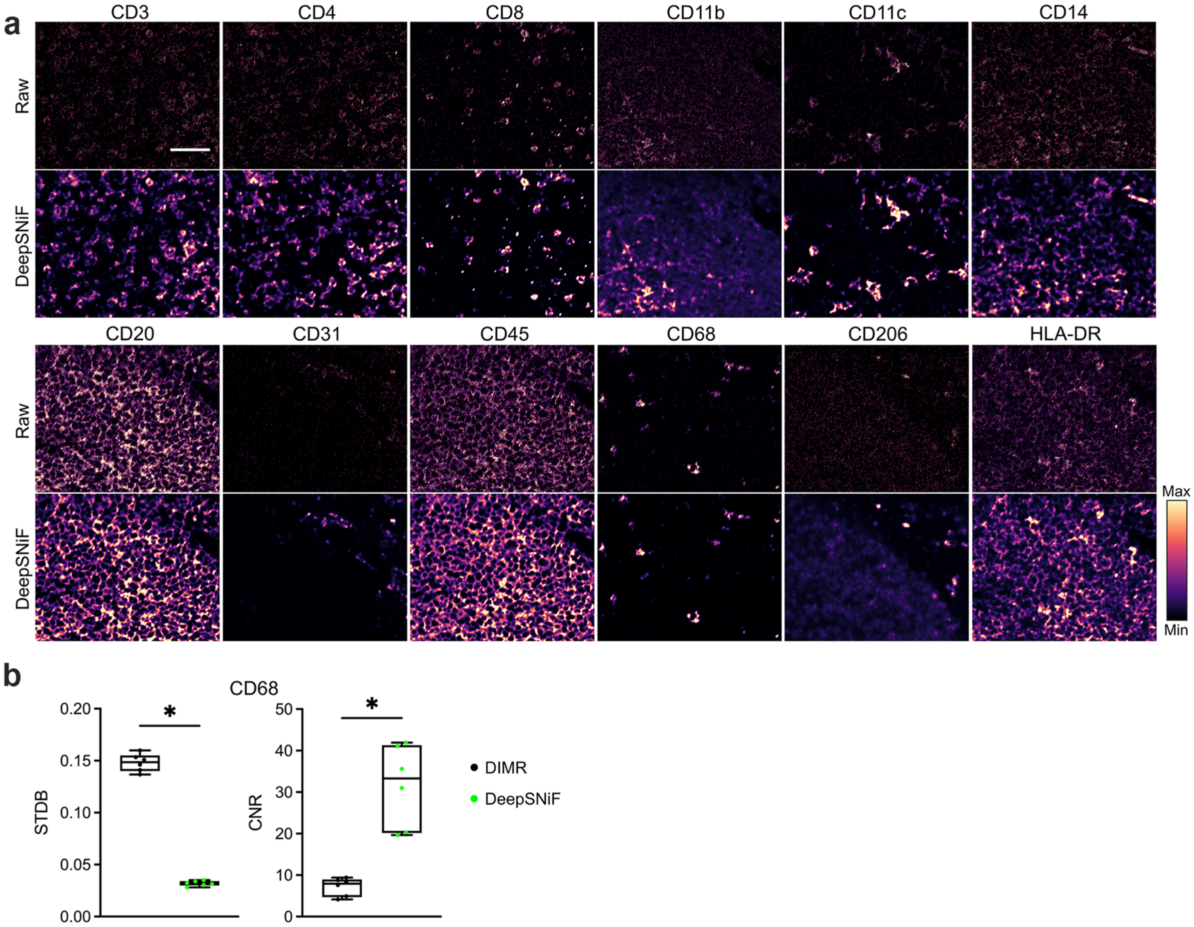


**Supplementary Figure 20.** IMC-Denoise enhances the MIBI dataset. Here, only DeepSNiF was applied to the MIBI images because no single hot pixels were observed. (a) Visual inspection of DIMR and DeepSNiF denoised IMC images labeled with different antibodies. (b) DeepSNiF significantly improves image qualities on denoising CD68-labeled IMC images in terms of STDB and CNR ($n = 6$ independent images). Box center indicates median, box edges 25th and 75th percentile, and whiskers minimum and maximum percentile. $P$ values were calculated through two-sided Wilcoxon matched-paired test (*$P$<0.05). Scale bar: 25 $\mu$m.

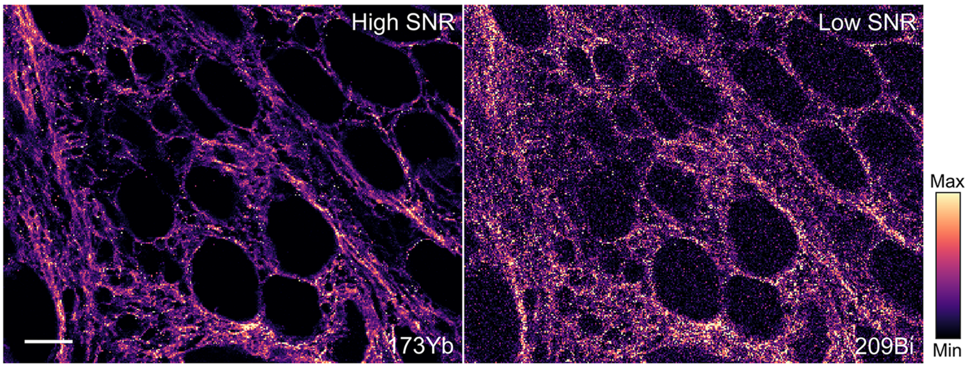


**Supplementary Figure 21.** The raw IMC images corresponding to Fig. 1g. Scale bar: 37 $\mu$m.

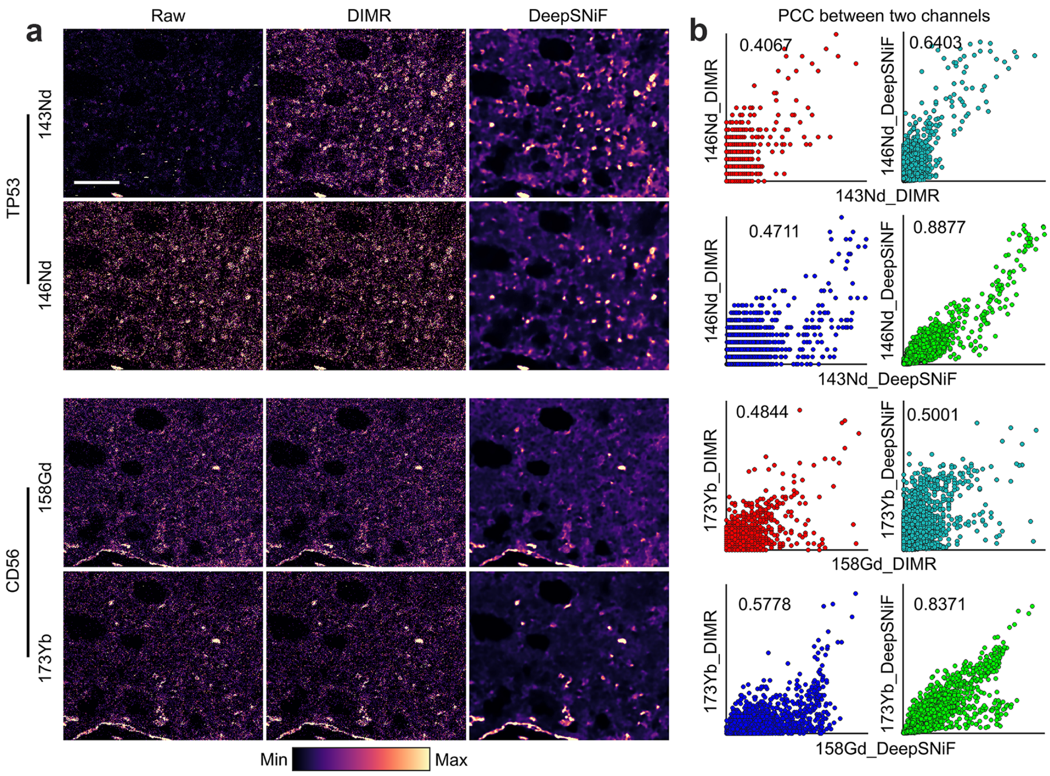


**Supplementary Figure 22.** IMC-Denoise enhances both IMC image quality and the Pearson correlations of IMC images labeled with the same markers. (a) A tissue was stained by 143Nd, 146Nd conjugated TP53, and 158Gd, 173Yb conjugated CD56, respectively, with different SNRs. The IMC images were firstly processed by DIMR to remove hot pixels. Then DeepSNiF was employed to improve the image quality of all the images, because the qualities of the higher SNR images are still sub-optimal. (b) After DeepSNiF processing, the Pearson correlation coefficients (PCC) improved, in which those of the double DeepSNiF-processed images are the highest. Notably, the DeepSNiF trained by the CD3 images from the human bone marrow dataset (Supplementary Tables 5 and 8) was used to denoise the DIMR-processed TP53 and CD56 images, due to their highly similar features and the lacking of sufficient TP53 and CD56 training sets. Scale bar: 75 $\mu$m.

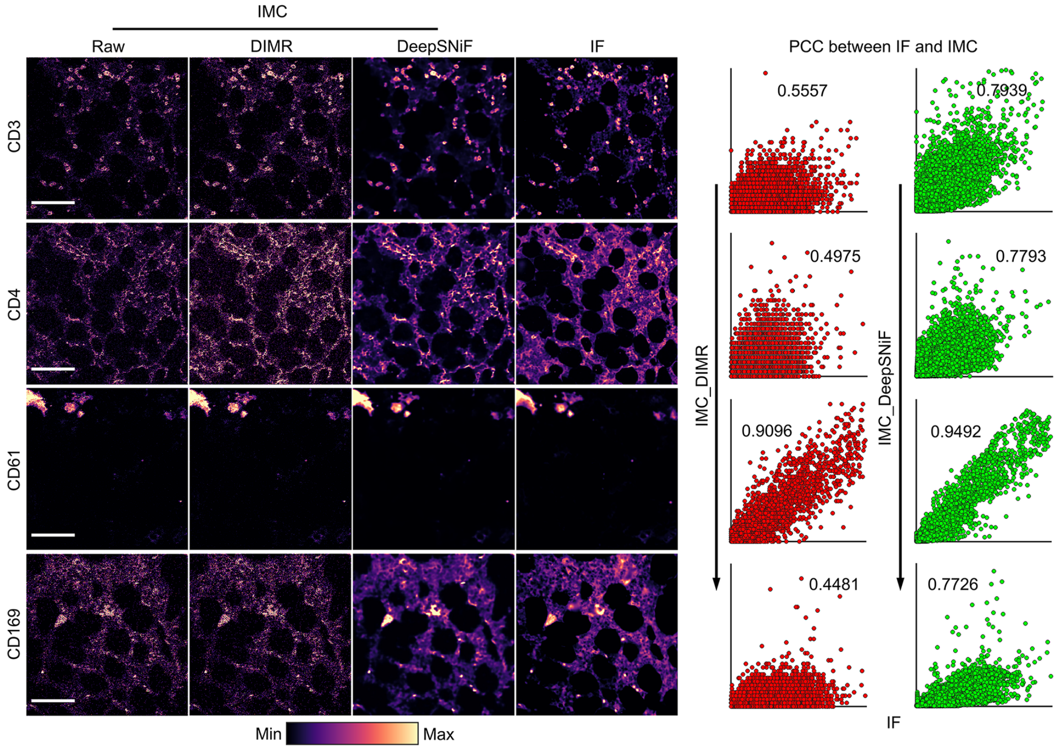


**Supplementary Figure 23.** IMC-Denoise enhances both IMC image quality and the Pearson correlations between IMC and IF images. (a) The same tissues were stained with CD3, CD4, CD61 and CD169 by IMC and IF, respectively. The low SNR IMC images were processed by DIMR to remove hot pixels and then by DeepSNiF to improve image quality. (b) After DeepSNiF processing, the PCC between IMC and IF improved, indicating DeepSNiF is able to improve the IMC image quality. Scale bar: CD3: 98 $\mu$m. CD4: 110 $\mu$m. CD61: 69 $\mu$m. CD169: 87 $\mu$m.

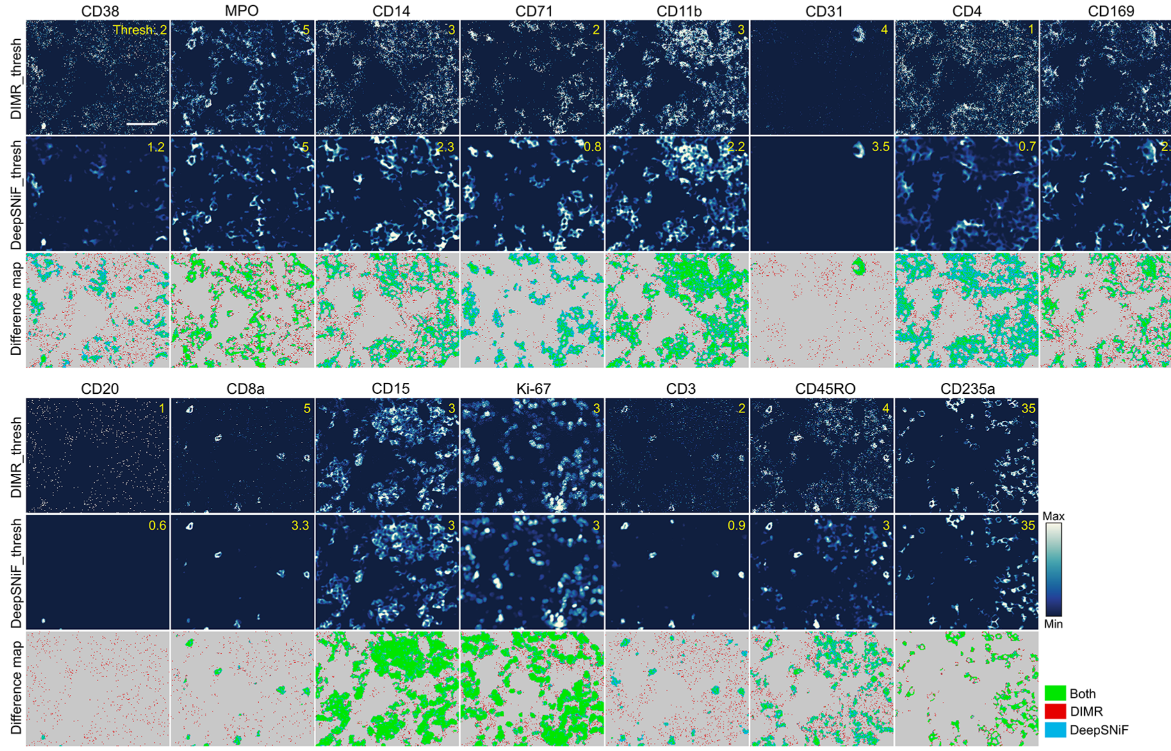


**Supplementary Figure 24.** DeepSNiF eliminates background noise of the IMC images from human bone marrow dataset. The raw IMC images were processed by DIMR to remove hot pixels and then by DeepSNiF to account for shot noise (Fig. 1f). Thresholds (upper right corner in every image) were selected to remove the background noise of the DIMR and DeepSNiF-processed images. The signal masks of DIMR and DeepSNiF-processed images were overlaid to compare the difference of background removal. Scale bar: 48 $\mu$m.

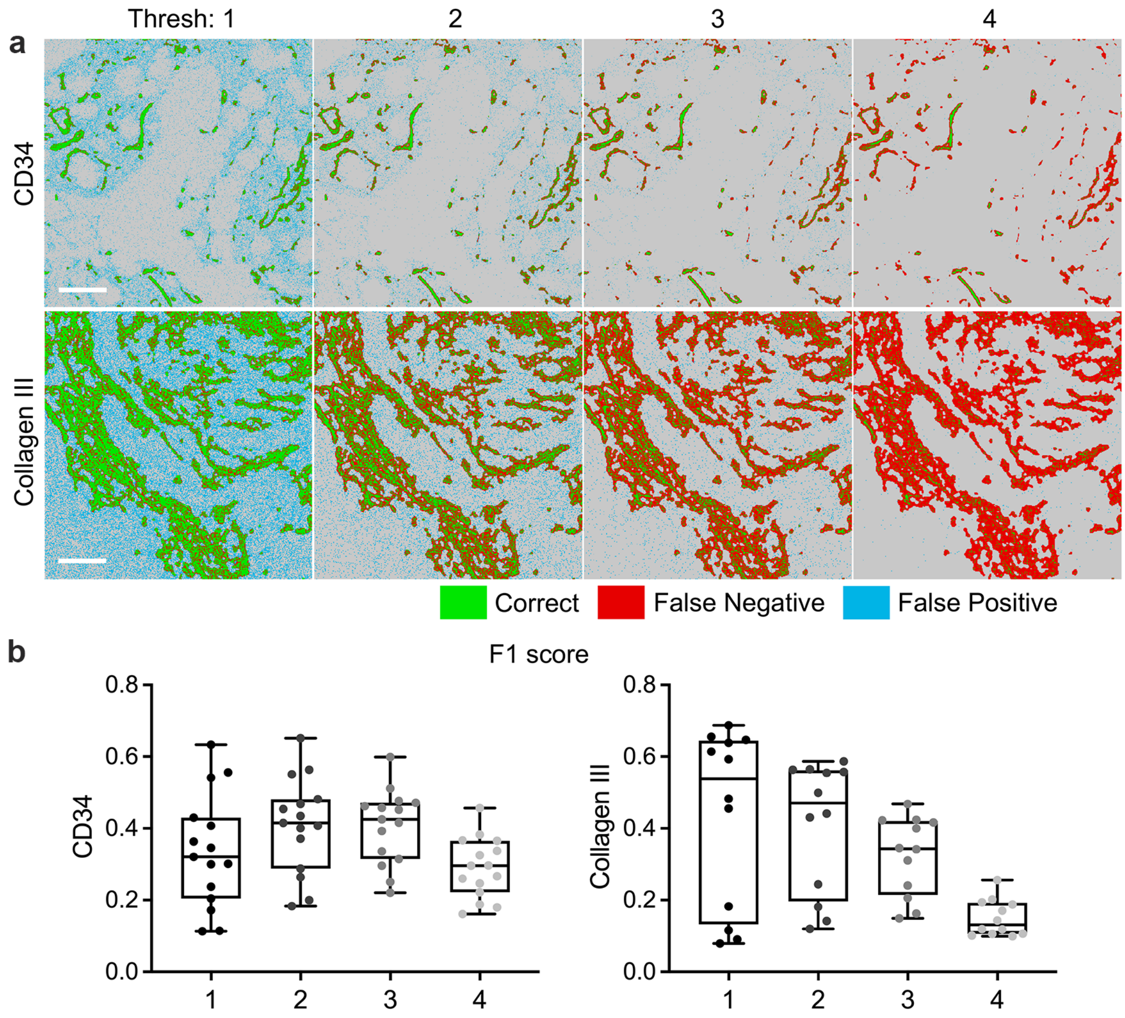


**Supplementary Figure 25.** Optimal threshold selection for DIMR_thresh method on background removal. Because the pixel values are non-negative integers in DIMR-processed images, threshold values from 1 to 4 are selected to remove background noise of DIMR-processed CD34 and Collagen III images ($n = 15$ independent images for CD34 and $n = 12$ independent images for Collagen III), and sub panel (a) and (b) represent the visual comparison and quantitative evaluations results, respectively. The optimal thresholds are then chosen for DIMR_thresh (CD34: 2; Collagen III: 1) to compare with other methods in Fig. 2b–c. In (b), box center indicates median, box edges 25th and 75th percentile, and whiskers minimum and maximum percentile. Scale bar: top: 50 $\mu$m, bottom: 75 $\mu$m.

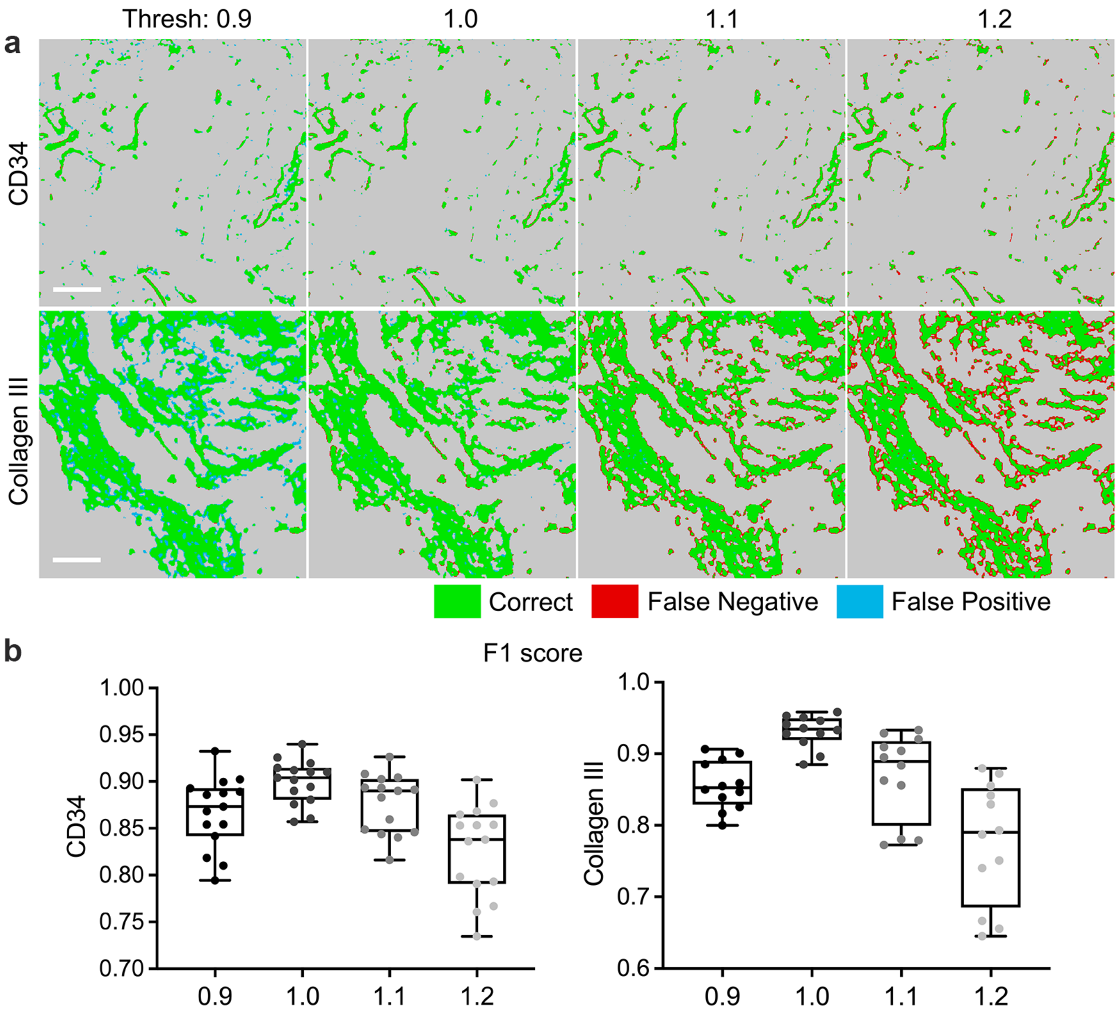


**Supplementary Figure 26.** Optimal threshold selection for DeepSNiF_thresh method on background removal. Threshold values from 0.9 to 1.2 are selected to remove background noise of DeepSNiF-processed CD34 and Collagen III images ($n = 15$ independent images for CD34 and $n = 12$ independent images for Collagen III), and sub panel (a) and (b) represent the visual comparison and quantitative evaluations results, respectively. The optimal thresholds are then chosen for DeepSNiF_thresh (CD34: 1; Collagen III: 1) to compare with other methods in Fig. 2b–c. In (b), box center indicates median, box edges 25th and 75th percentile, and whiskers minimum and maximum percentile. Scale bar: top: 50 $\mu$m, bottom: 75 $\mu$m.

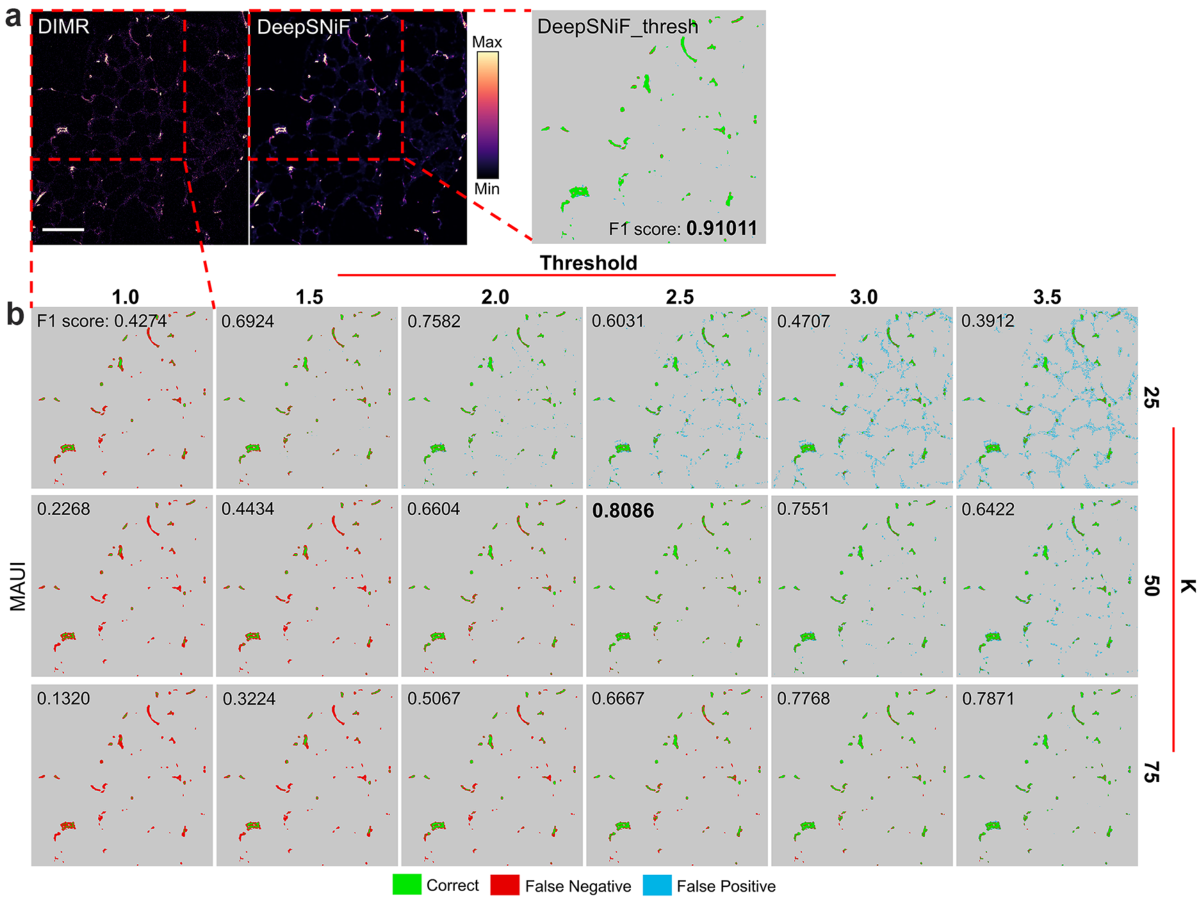


**Supplementary Figure 27.** DeepSNiF performs better than MAUI on DIMR data to filter background noise. (a) A DIMR and DeepSNiF-processed IMC image labeled with CD34. The DeepSNiF-processed image was binarized by the threshold value 1 and then overlaid with manual annotated ground truth. (b) The DIMR-processed image was processed by the MAUI software package with a wide range of parameters to select the best background noise removal result and also overlaid with the manual annotated ground truth. The F1 score of the DeepSNiF_thresh (lower right corner) result is always higher than that of the MAUI results (upper left corner in every image), indicating DeepSNiF is better than MAUI on DIMR data in terms of background noise removal. Scale bar: 96 $\mu$m.

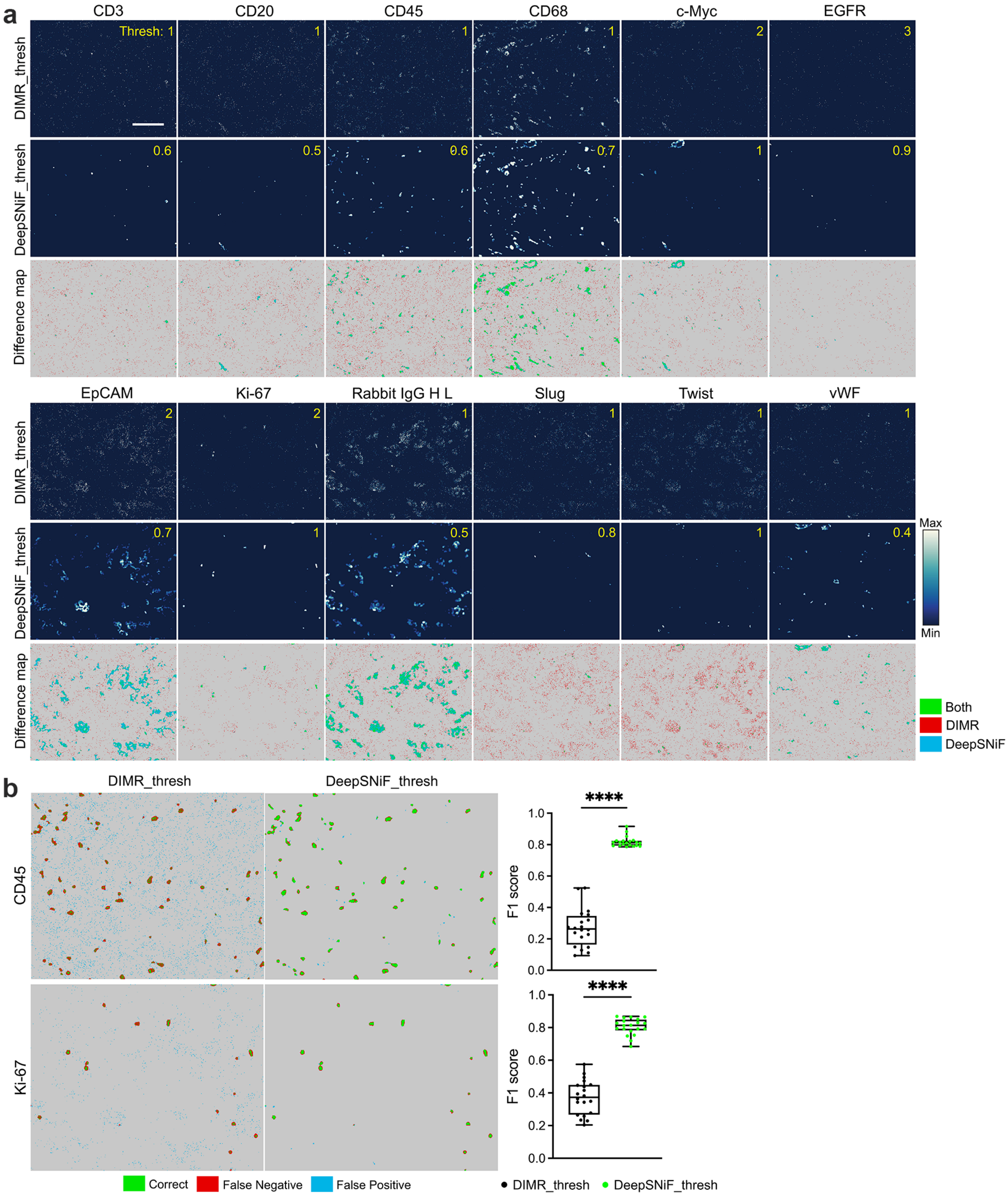


**Supplementary Figure 28.** DeepSNiF eliminates background noise of the IMC images from human breast cancer dataset (Supplementary Fig. 18). (a) Visual inspection of background removal results of DIMR and DeepSNiF-processed images. Each marker images are binarized with the thresholds on the upper right corner in every image, respectively. The signal masks of DIMR and DeepSNiF-processed images were overlaid to compare the difference of background removal. (b) Manual annotated images are served as ground truths. After DeepSNiF denoising, the background removal accuracy improves significantly in terms of F1 score, for both CD45 and Ki-67-labeled images ($n$ = 20 independent images for each marker). Box center indicates median, box edges 25th and 75th percentile, and whiskers minimum and maximum percentile. $P$ values were calculated through two-sided Wilcoxon matched-paired test (****$P$<0.0001). Scale bar: 100 $\mu$m.

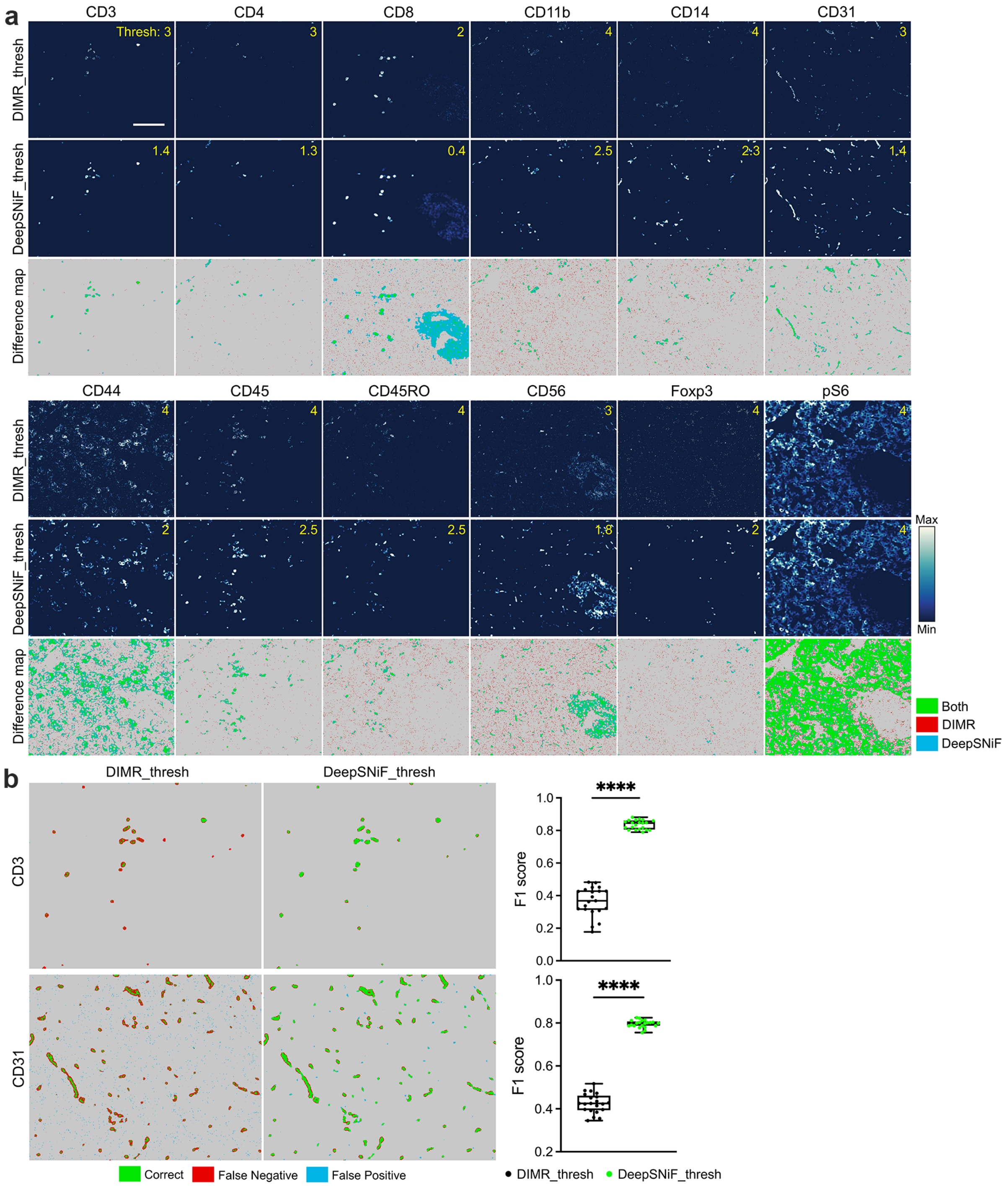


**Supplementary Figure 29.** DeepSNiF eliminates background noise of the IMC images from human pancreas cancer dataset (Supplementary Fig. 19). (a) Visual inspection of background removal results of DIMR and DeepSNiF-processed images. Each marker images are binarized with the thresholds on the upper right corner in every image, respectively. The signal masks of DIMR and DeepSNiF-processed images were overlaid to compare the difference of background removal. (b) Manual annotated images are served as ground truths. After DeepSNiF denoising, the background removal accuracy improves significantly in terms of F1 score, for both CD3 and CD31-labeled images ($n$ = 21 independent images for each marker). Box center indicates median, box edges 25th and 75th percentile, and whiskers minimum and maximum percentile. $P$ values were calculated through two-sided Wilcoxon matched-paired test (****$P$<0.0001). Scale bar: 100 $\mu$m.

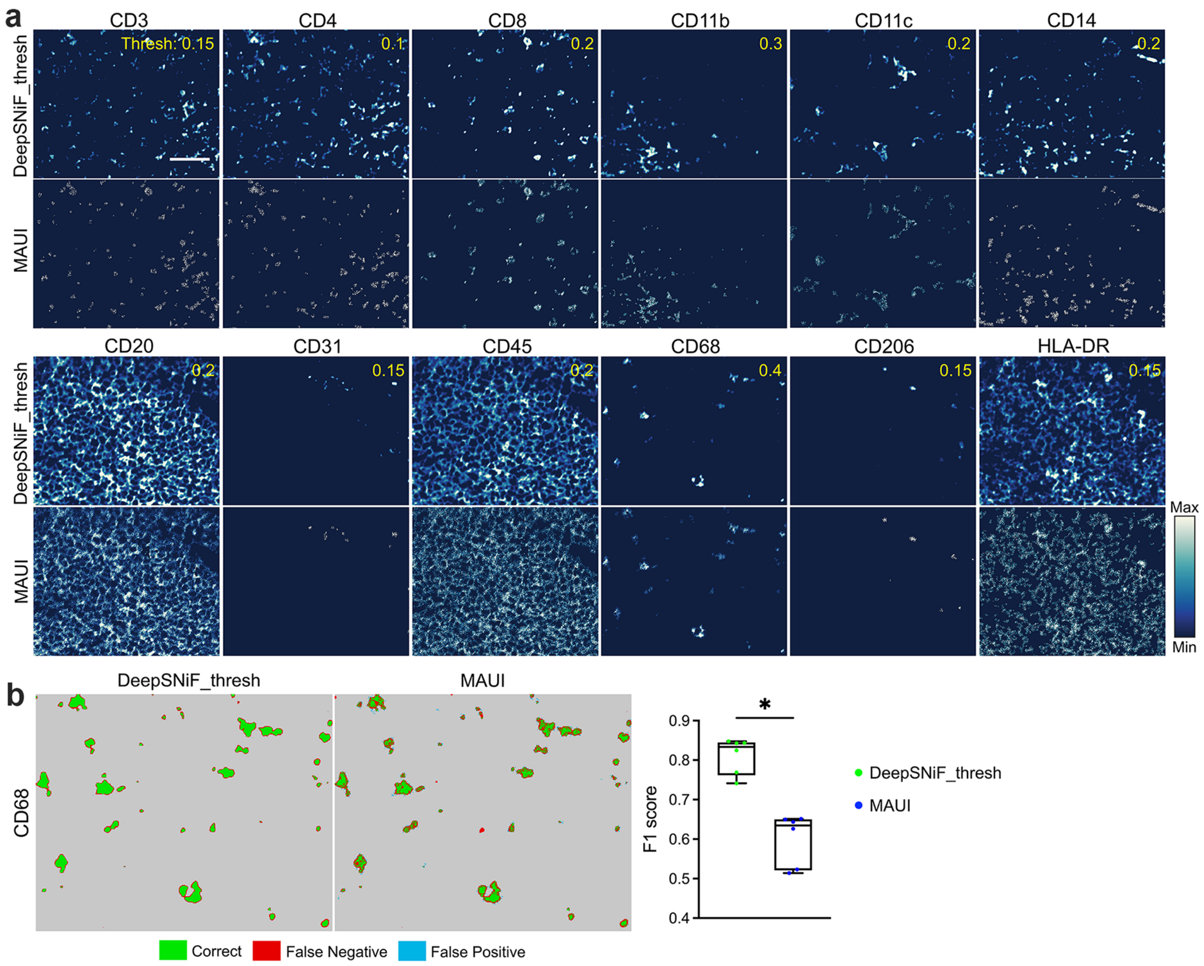


**Supplementary Figure 30.** DeepSNiF eliminates background noise of the MIBI images (Supplementary Fig. 20). (a) Visual inspection of background removal results of DeepSNiF and MAUI-processed images. Each marker images are binarized with the thresholds on the upper right corner in every image, respectively. (b) Manual annotated images are served as ground truths. The background removal accuracy of DeepSNiF is higher than that of MAUI in terms of F1 score, for CD68 ($n = 6$ independent images), as an example. Box center indicates median, box edges 25th and 75th percentile, and whiskers minimum and maximum percentile. $P$ values were calculated through two-sided Wilcoxon matched-paired test (*$P$<0.05). Scale bar: 25 $\mu$m.

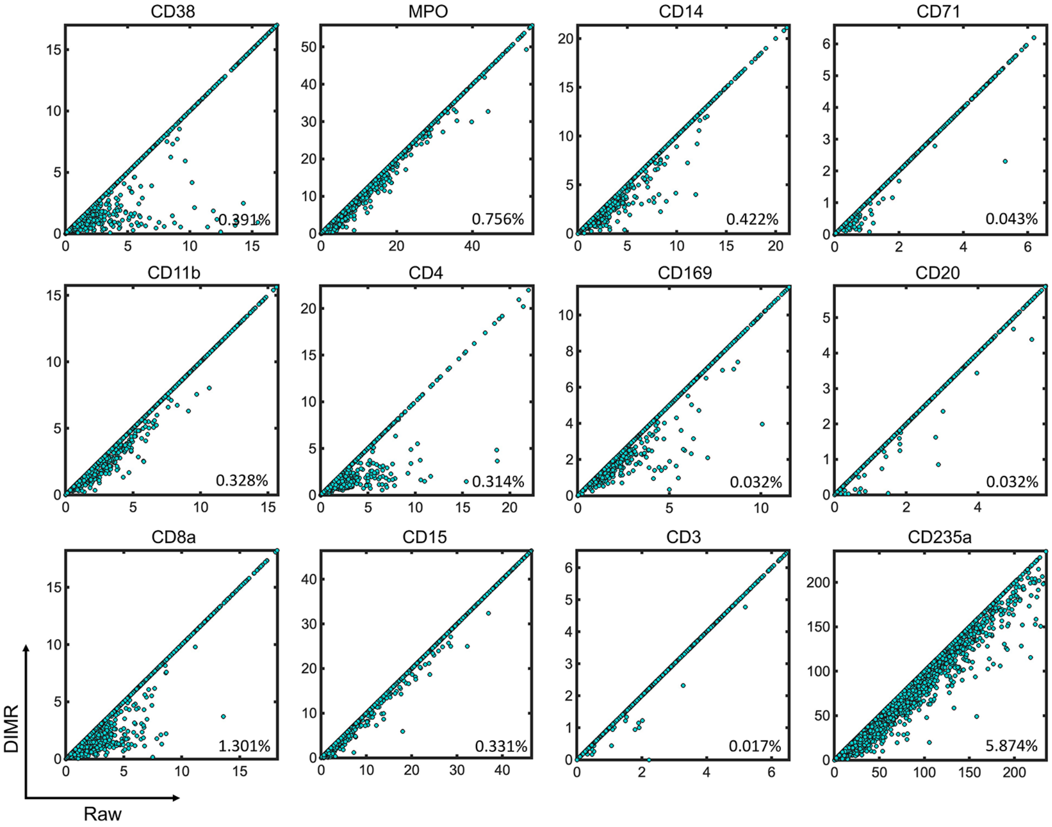


**Supplementary Figure 31.** The impact of DIMR on single cell data extracted from DeepSNiF-based cell segmentaton masks. Each sub-figure represents the one-on-one relationship between the raw and DIMR data of a particular marker in single cell scale. The bottom right value in each sub-figure represents the percentage of the difference between the raw and DIMR data.

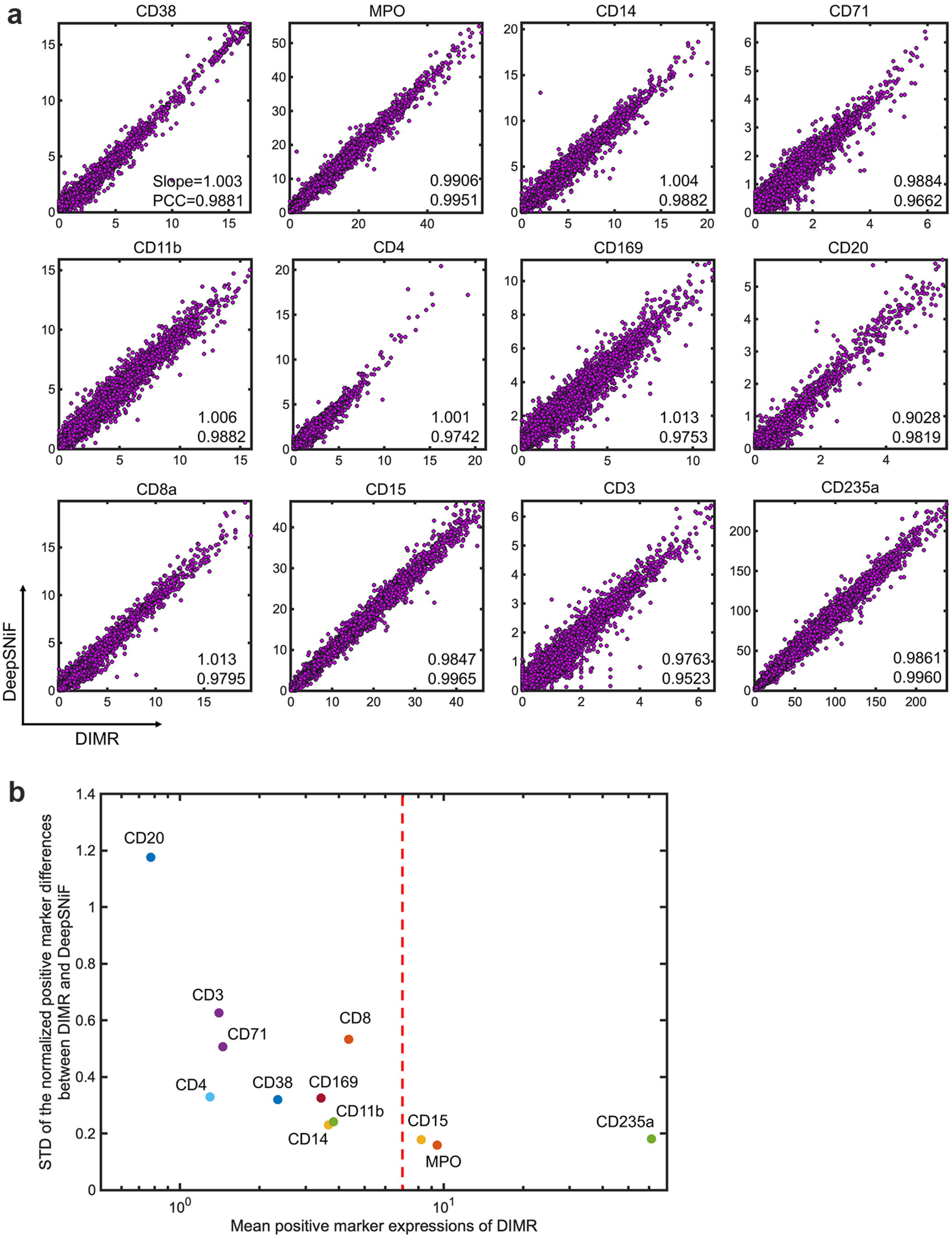


**Supplementary Figure 32.** The impact of DeepSNiF on single cell data extracted from DeepSNiF-based cell segmentaton masks. (a) Each sub-figure represents the one-on-one relationship between the DIMR and DeepSNiF data of a particular marker in single cell scale. The bottom right value in each sub-figure represents the slope of the line fitting results and the PCC between the DIMR and DeepSNiF data. These values indicate the DIMR and DeepSNiF single cell data are at the same scale and linearly correlated. (b) Since the DIMR and DeepSNiF data are highly correlated, the standard deviation (STD) of the normalized positive marker differences between DIMR and DeepSNiF are utilized to evaluate the impact of DeepSNiF on single cell data. For almost all the markers, the larger the mean positive marker expressions, the smaller the STD will be, and then the lighter the impact of DeepSNiF will be. This agrees with the fact that the larger the ion count is, the lower the shot noise level will be. Note that in (b), the data was 99th-percentile normalized before calculation to mitigate the impact of extreme values.

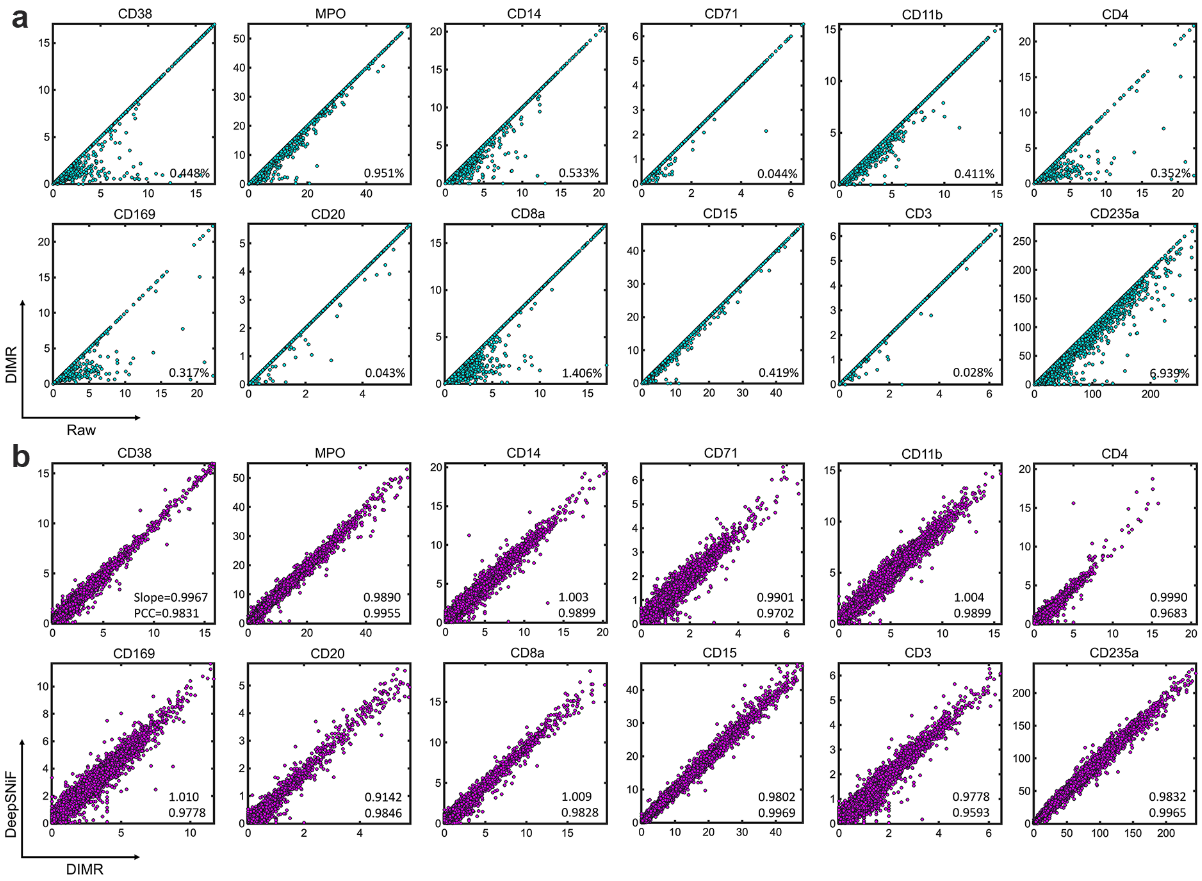


**Supplementary Figure 33.** The impact of DIMR on single cell data extracted from DIMR-based cell segmentaton masks. (a) Each sub-figure represents the one-on-one relationship between the raw and DIMR data of a particular marker in single cell scale. The bottom right value in each sub-figure represents the percentage of the difference between the raw and DIMR data. (b) Each sub-figure represents the one-on-one relationship between the DIMR and DeepSNiF data of a particular marker in single cell scale. The bottom right value in each sub-figure represents the slope of the line fitting results and the PCC between the DIMR and DeepSNiF data. These values indicate the DIMR and DeepSNiF single cell data are at the same scale and linearly correlated.

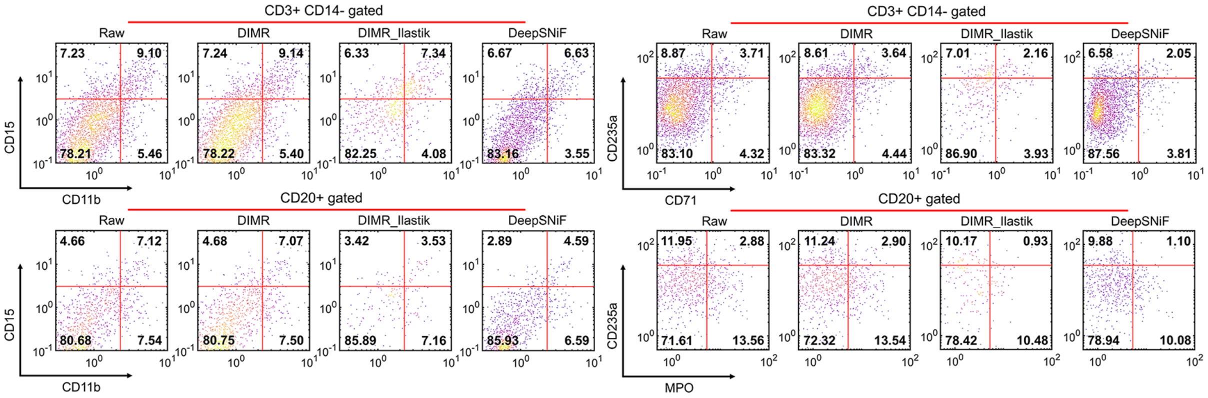


**Supplementary Figure 34.** Evaluations of denoising algorithms with manual gating strategies on single cell data extracted from DIMR-based cell segmentaton masks. The numbers in these panels are the cell percentages of the corresponding ranges. DIMR slightly enhances the single cell analysis over raw data, while DeepSNiF further enhances the DIMR results and performs better than semi-automated DIMR_Ilastik-processing.

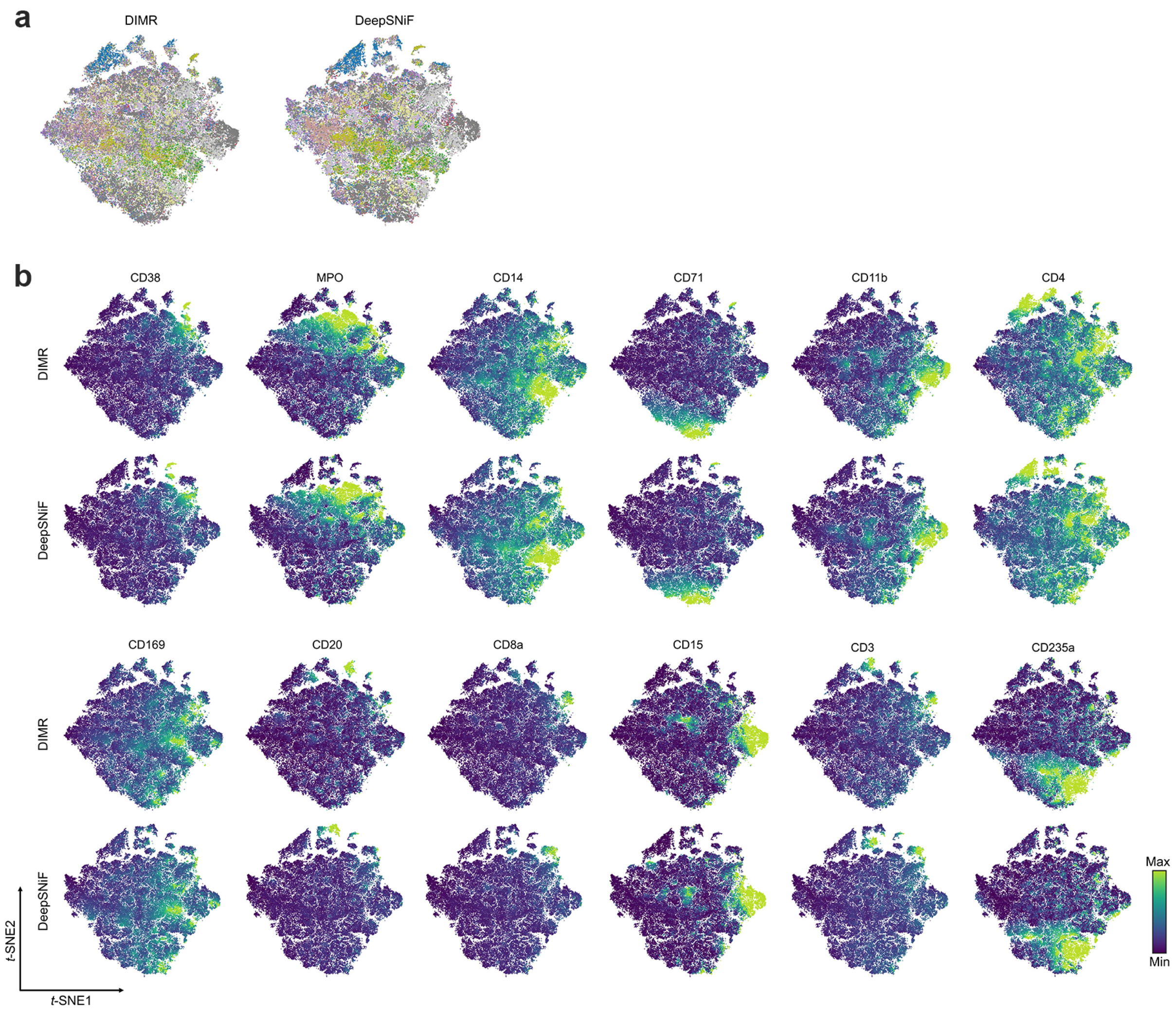


**Supplementary Figure 35.** *t*-SNE plots of the single cell data of the human bone marrow IMC dataset extracted from DeepSNiF-based cell segmentaton masks. (a) *t*-SNE plots colored by the cells from different tissues. (b) *t*-SNE plots colored by the single cell marker expressions of DIMR and DeepSNiF, respectively.

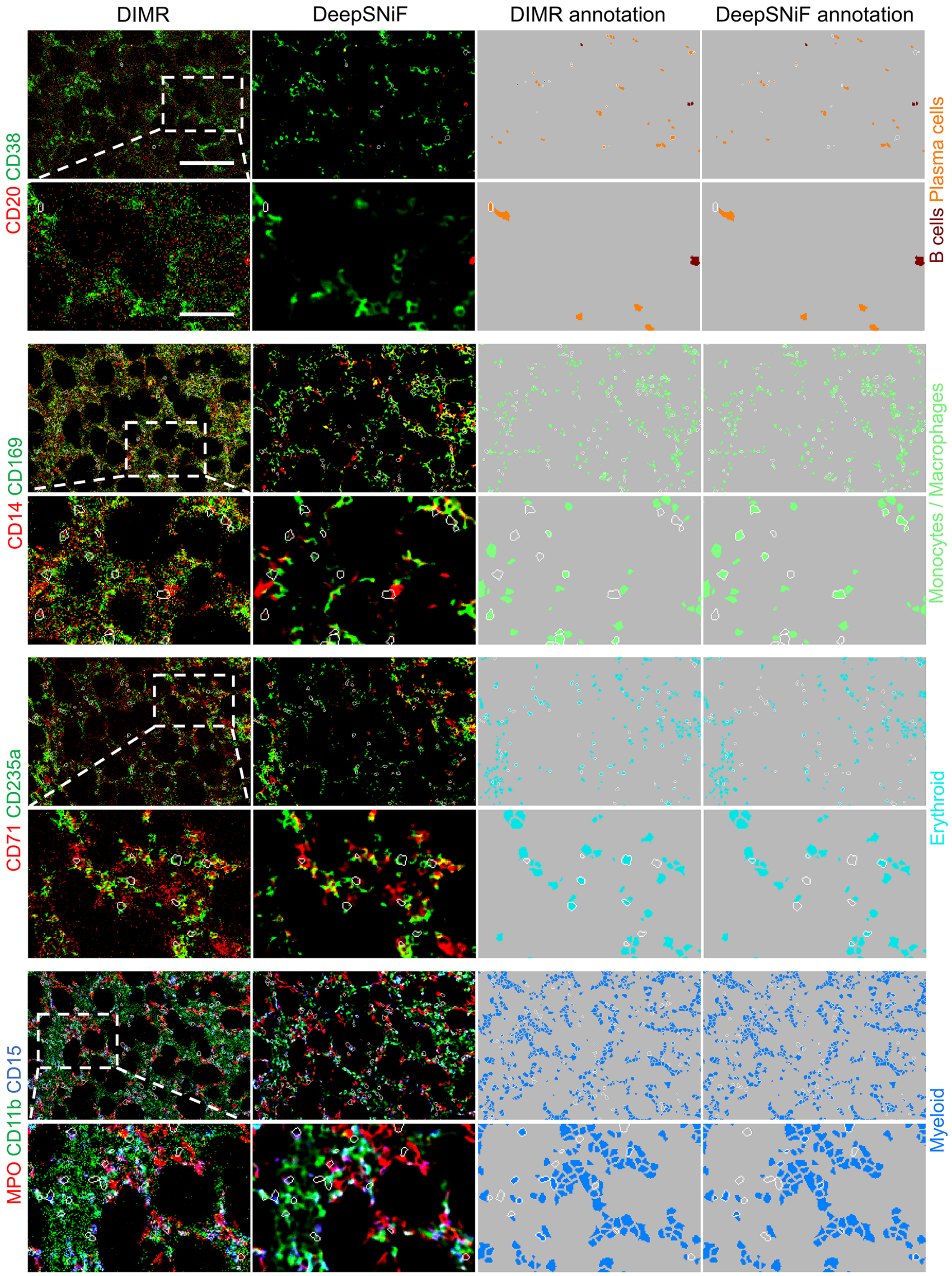


**Supplementary Figure 36.** Comparisons of DIMR and DeepSNiF-processed IMC images labeled with different cell markers, and the corresponding cell annotation results with the DeepSNiF-based cell segmentaton masks (Fig. 3(c)). The bottom row corresponds to the white dashed box regions in the top row images. The white contours represent the different phenotyping results between DIMR and DeepSNiF. Scale bar: Top: 145 $\mu$m, bottom: 50 $\mu$m.

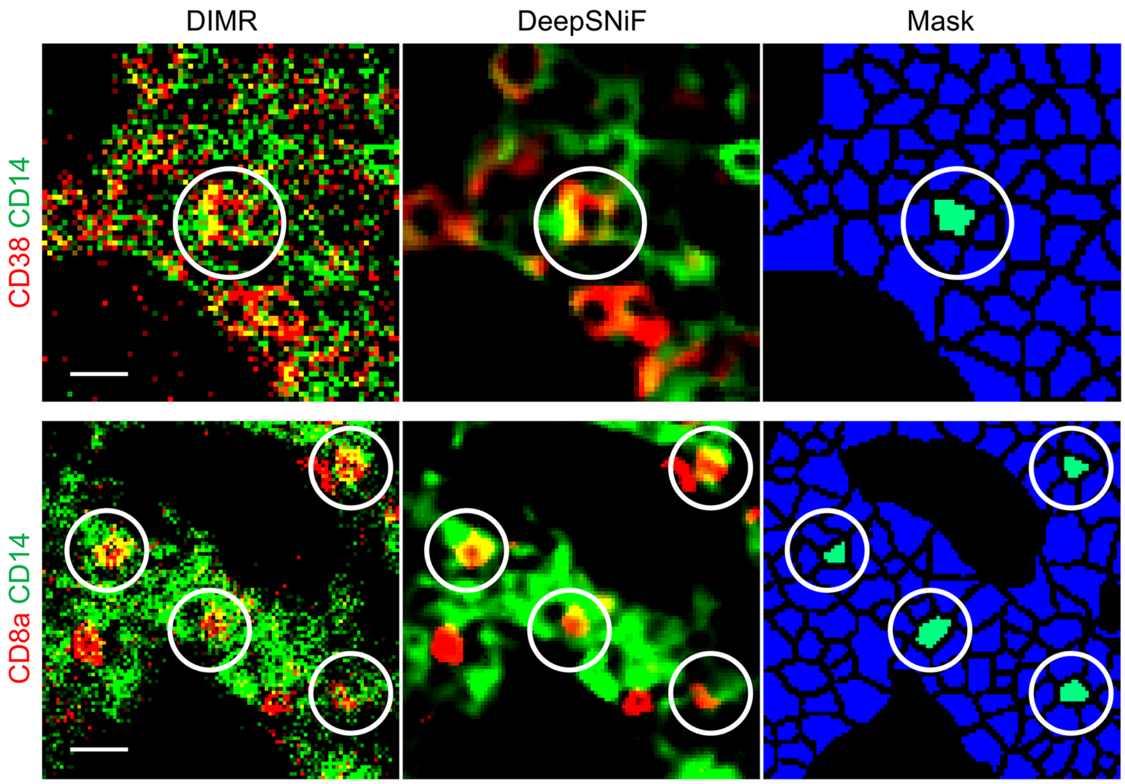


**Supplementary Figure 37.** Examples of double CD38+ CD14+ (top) and double CD8a+ CD14+ (bottom) cells. Scale bar: top: 11 $\mu$m, bottom: 16 $\mu$m.

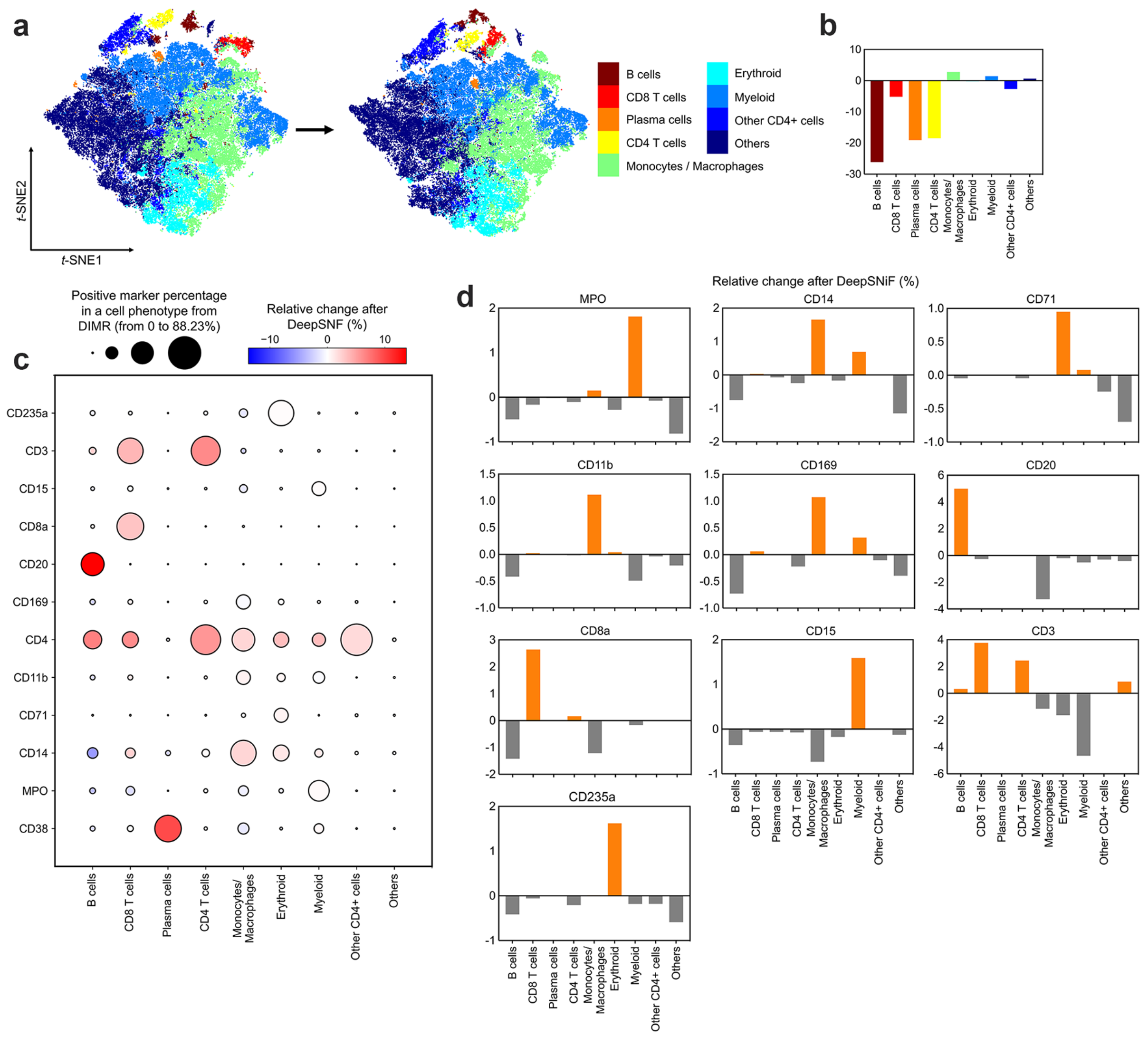


**Supplementary Figure 38.** DeepSNiF enhances automated cell phenotyping on human bone marrow IMC data with DIMR-based cell segmentaton masks. (a) *t*-SNE plots of DIMR and DeepSNiF with cell phenotyping results. (b) The relative change in cell phenotypes before and after DeepSNiF. (c) DeepSNiF enhances the sensitivity of cell phenotyping. After DeepSNiF processing, the non-specific marker signals reduce while the specific ones enrich in the cell types, respectively. The circle size indicates the positive marker percentage in a particular phenotype of DIMR, and the circle colour indicates the relative changes of the positive rate for the particular markers after DeepSNiF enhancement. (d) DeepSNiF enhances the specificity of cell phenotyping. With DeepSNiF denoising, the ratios of specific phenotypes increase while those of non-specific phenotypes decrease in the positive markers. The relative change is the difference in percentage composition of each cell type before and after DeepSNiF enhancement.

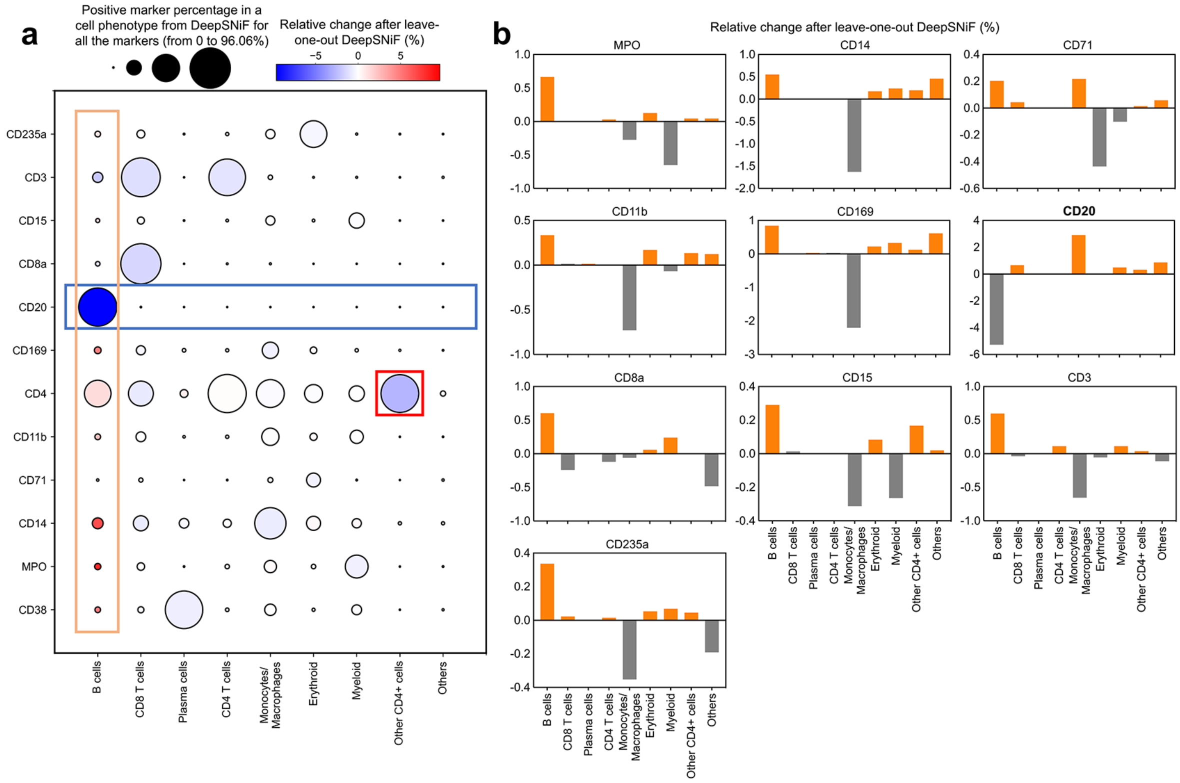


**Supplementary Figure 39.** The impact of CD20 denoising on cell phenotyping with DeepSNiF-based cell segmentaton masks. Only CD20 was processed by DIMR while all the other markers were denoised by both DIMR and DeepSNiF. Then the leave-one-out DeepSNiF phenotyping result was compared to the DeepSNiF for all the markers. (a) The sensitivity of cell phenotyping reduces for B cells. The specific marker signals reduce while the non-specific ones enrich in the B cells, respectively. The impacts on other cell types are limited compared to B cells. The circle size indicates the positive marker percentage in a particular phenotype from DeepSNiF for all the markers, and the circle color indicates the relative changes of the positive rate for the particular markers after processed by the leave-one-out DeepSNiF. (b) The specificity of cell phenotyping on CD20+ cells reduces. The ratio of B cells decreases while those of non-specific phenotypes increase in the CD20+ cells. The specificity of cell phenotyping on other positive markers, such as CD14 and CD169, also reduce, but the extent is limited.

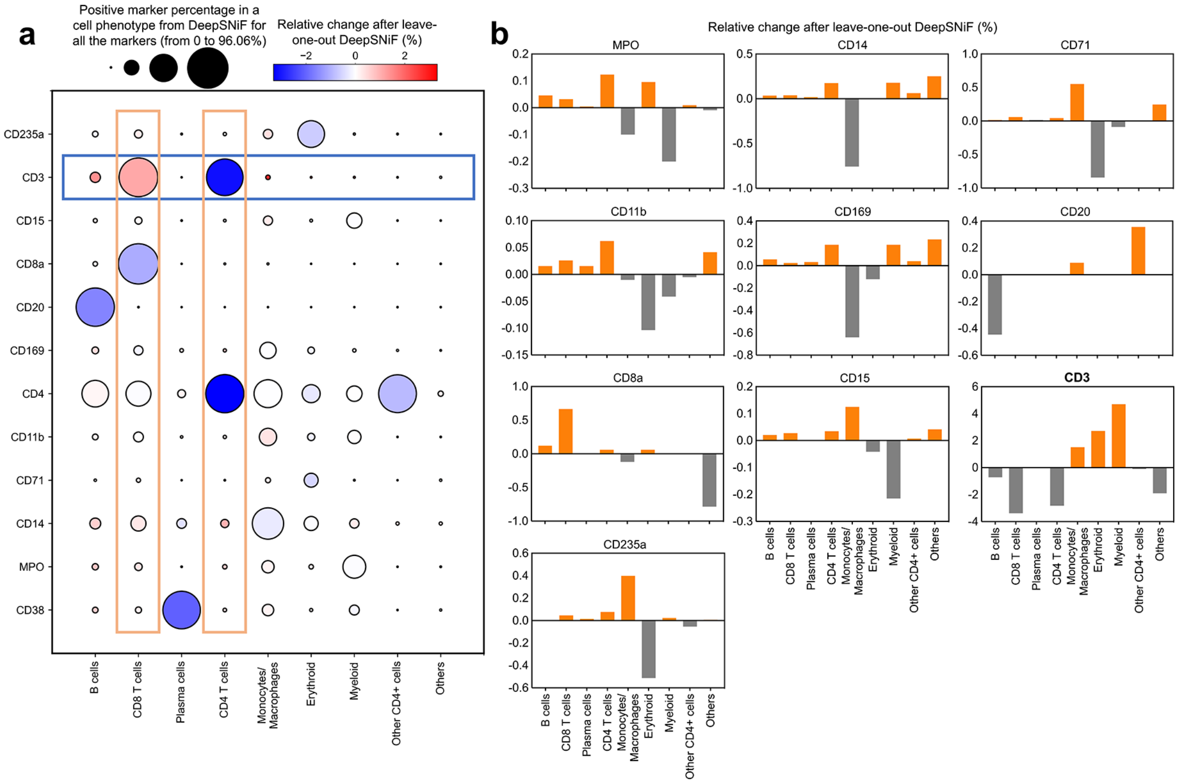

**Supplementary Figure 40.** The impact of CD3 denoising on cell phenotyping with DeepSNiF-based cell segmentaton masks. Only CD3 was processed by DIMR while all the other markers were denoised by both DIMR and DeepSNiF. Then the leave-one-out DeepSNiF phenotyping result was compared to the DeepSNiF for all the markers. (a) The sensitivity of cell phenotyping reduces for CD8 and CD4 T cells. The specific marker signals reduce while the non-specific ones enrich in the CD4 T cells, respectively. For CD8 T cells, even though the ratio of CD3+ cells increases, the ratio of CD8a+ cells decreases slightly. Overall, the sensitivity for CD8 T cells also decreases. The impacts on other cell types are limited. The circle size indicates the positive marker percentage in a particular phenotype from DeepSNiF for all the markers, and the circle color indicates the relative changes of the positive rate for the particular markers after processed by the leave-one-out DeepSNiF. (b) The specificity of cell phenotyping on CD3+ cells reduces. The ratios of CD8 and CD4 T cells decrease while those of non-specific phenotypes increase in the CD3+ cells. The specificity of cell phenotyping on other positive markers, such as MPO and CD8a, also reduces, but the extent is limited.

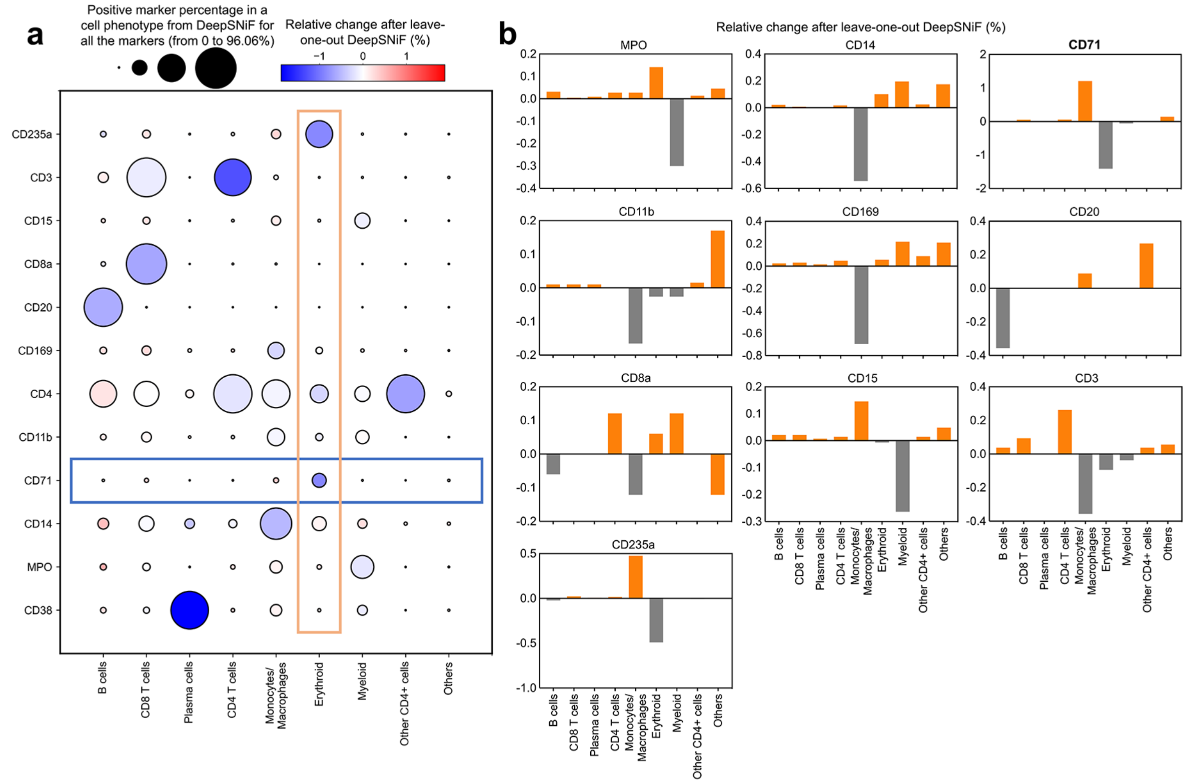


**Supplementary Figure 41.** The impact of CD71 denoising on cell phenotyping with DeepSNiF-based cell segmentaton masks. Only CD71 was processed by DIMR while all the other markers were denoised by both DIMR and DeepSNiF. Then the leave-one-out DeepSNiF phenotyping result was compared to the DeepSNiF for all the markers. (a) The sensitivity of cell phenotyping reduces for erythroid cells. The specific marker signals reduce while the non-specific ones enrich in the erythroids, respectively. The impacts on other cell types are limited compared to erythroid cells. The circle size indicates the positive marker percentage in a particular phenotype from DeepSNiF for all the markers, and the circle color indicates the relative changes of the positive rate for the particular markers after processed by the leave-one-out DeepSNiF. (b) The specificity of cell phenotyping on CD71+ cells reduces. The ratio of erythroids decreases while those of non-specific phenotypes increase in the CD71+ cells. Overall, the impact of CD71 denoising is smaller than those of CD20 and CD3, which corresponds to Supplementary Fig. 32(b).

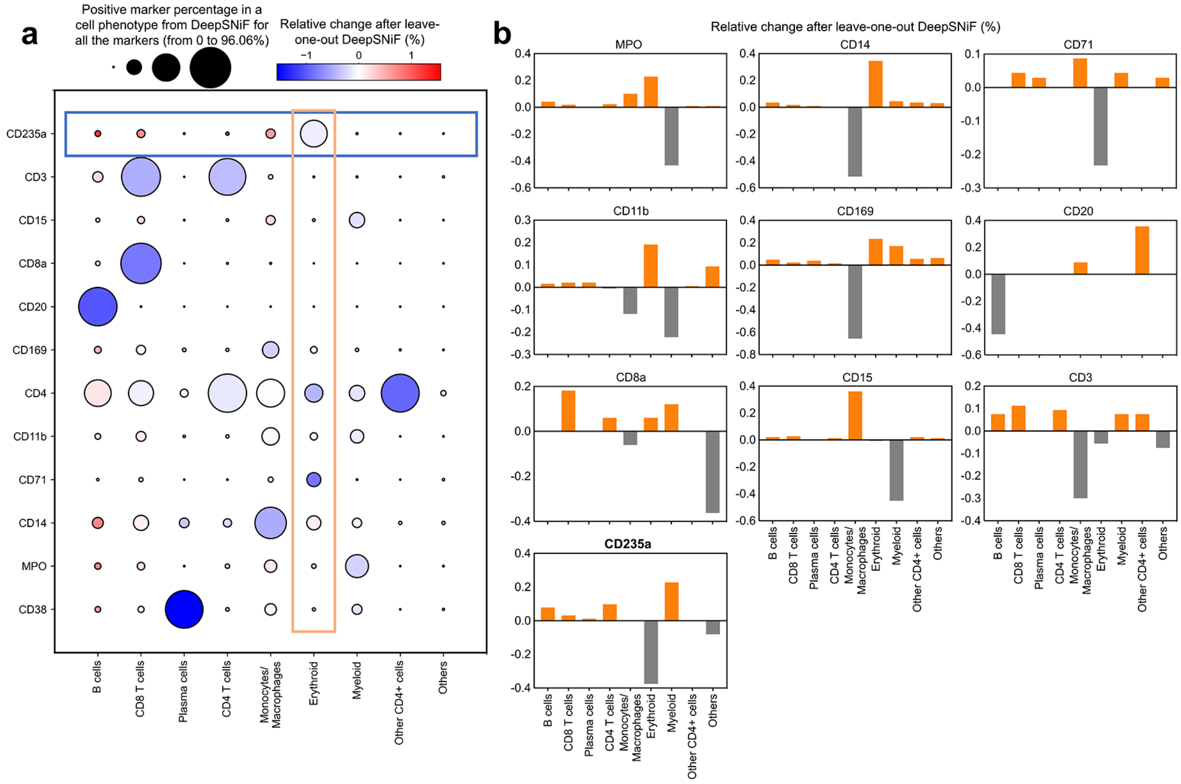


**Supplementary Figure 42.** The impact of CD235a denoising on cell phenotyping with DeepSNiF-based cell segmentaton masks. Only CD235a was processed by DIMR while all the other markers were denoised by both DIMR and DeepSNiF. Then the leave-one-out DeepSNiF phenotyping result was compared to the DeepSNiF for all the markers. (a) The sensitivity of cell phenotyping reduces slightly for erythroid cells. The specific marker signals reduce while the non-specific ones enrich in the erythroids, respectively. The circle size indicates the positive marker percentage in a particular phenotype from DeepSNiF for all the markers, and the circle color indicates the relative changes of the positive rate for the particular markers after processed by the leave-one-out DeepSNiF. (b) The specificity of cell phenotyping on CD235a+ cells reduces slightly. The ratio of erythroid cells decreases while those of non-specific phenotypes increase in the CD235a+ cells. Overall, the impact of CD235a denoising is limited compared to CD3, CD20 and CD71 because of the high SNR of CD235a IMC images, which corresponds to Supplementary Fig. 32(b).

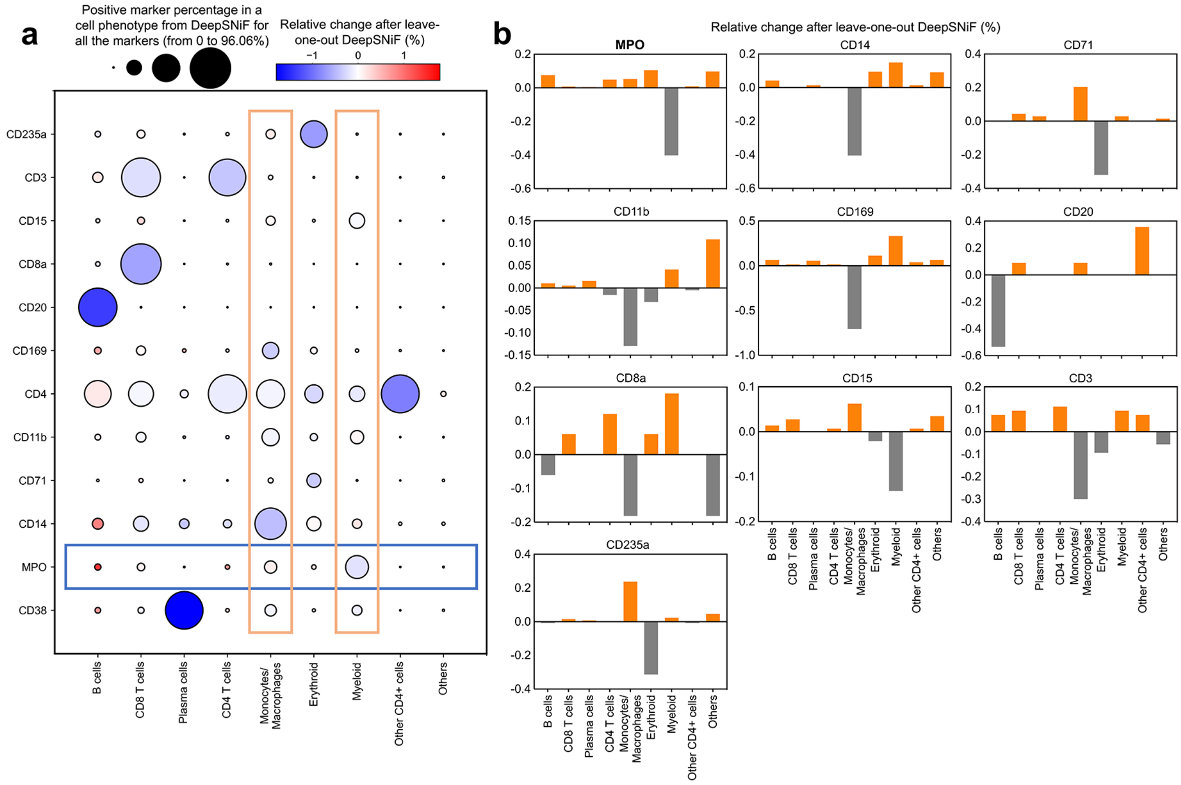


**Supplementary Figure 43.** The impact of MPO denoising on cell phenotyping with DeepSNiF-based cell segmentaton masks. Only MPO was processed by DIMR while all the other markers were denoised by both DIMR and DeepSNiF. Then the leave-one-out DeepSNiF phenotyping result was compared to the DeepSNiF for all the markers. (a) The sensitivity of cell phenotyping reduces slightly for myeloid cells and monocytes/macrophages. The specific marker signals reduce while the non-specific ones enrich in the myeloid cells and monocytes/macrophages, respectively. The circle size indicates the positive marker percentage in a particular phenotype from DeepSNiF for all the markers, and the circle color indicates the relative changes of the positive rate for the particular markers after processed by the leave-one-out DeepSNiF. (b) The specificity of cell phenotyping on MPO+ cells reduces slightly. The ratios of monocytes/macrophages and myeloid cells decrease while those of non-specific phenotypes increase in the MPO+ cells. Overall, the impact of MPO denoising is limited compared to CD3, CD20 and CD71 because of the high SNR of MPO IMC images, which corresponds to Supplementary Fig. 32(b).

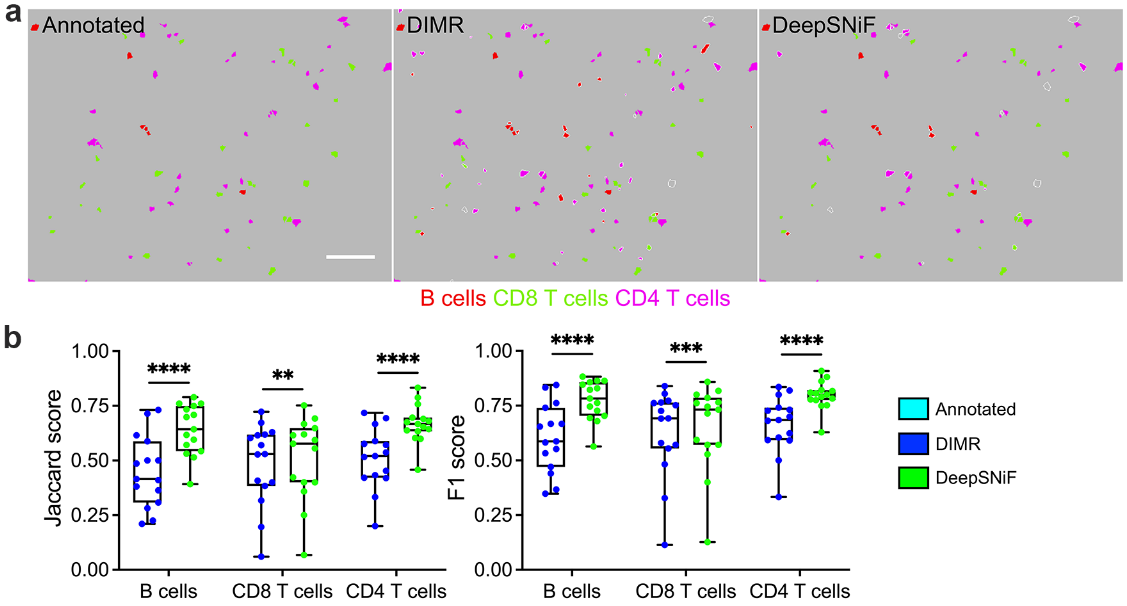


**Supplementary Figure 44.** DeepSNiF enhances lymphocyte analysis. (a) Manual annotations for lymphocytes and comparisons with DIMR and DeepSNiF phenotyping results with DIMR-based cell masks. The white contours represent the differential phenotyping results between the annotated and DIMR/DeepSNiF results. (b) Annotation evaluations of DIMR and DeepSNiF by both Jaccard and F1 scores across the tissues ($n$ = 15 biologically independent samples). Box center indicates median, box edges 25th and 75th percentile, and whiskers minimum and maximum percentile. $P$ values were calculated through two-sided Wilcoxon matched-paired test (**$P$<0.01, ***$P$<0.001 and ****$P$<0.0001). Scale bar: 85 $\mu$m.

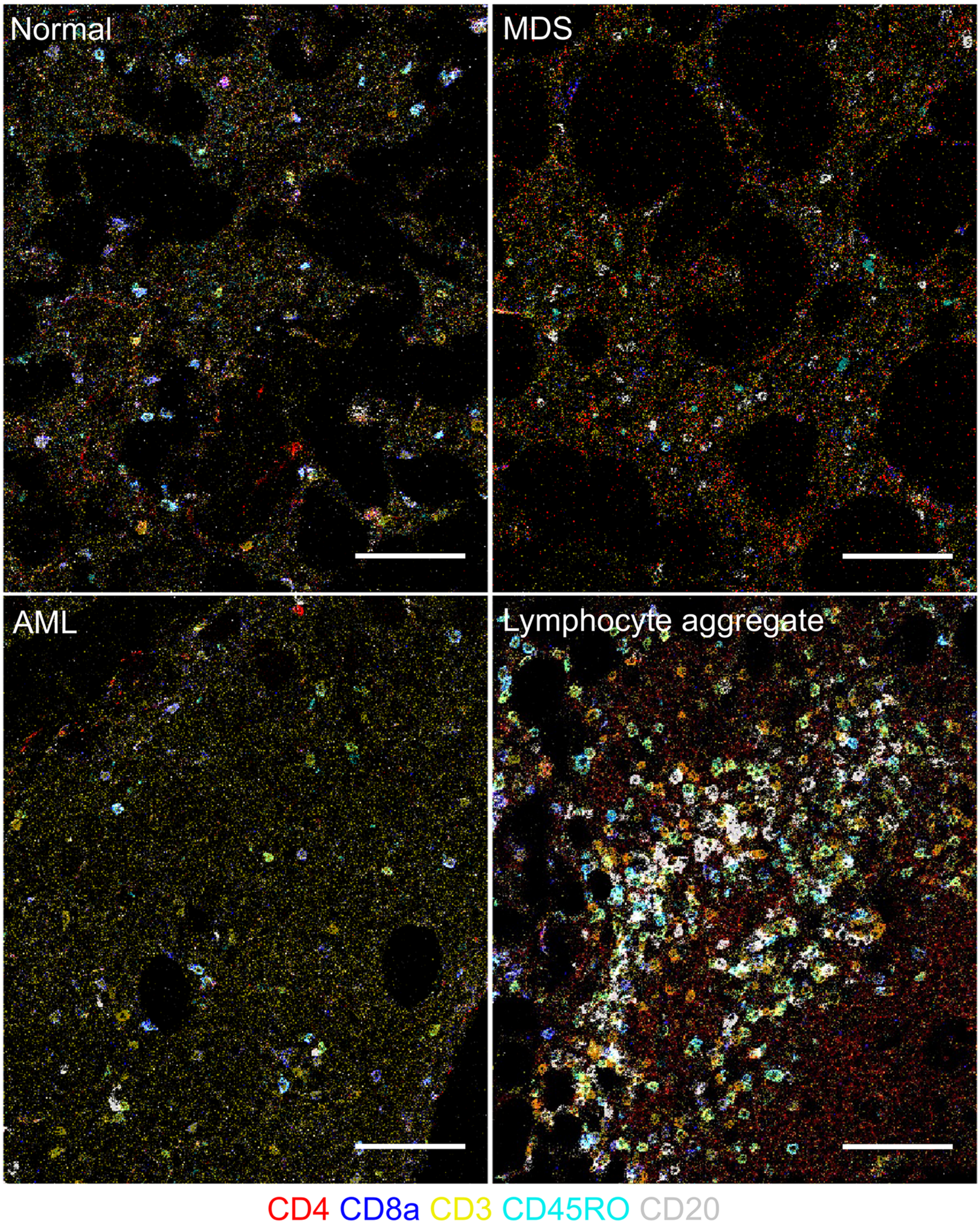


**Supplementary Figure 45.** Representative images of lymphocyte markers before DeepSNiF denoising from specimens of normal (upper left), MDS (upper right), AML (lower left) and AML with lymphoid aggregate (lower right) tissue samples. Scale bar: 112 $\mu$m.

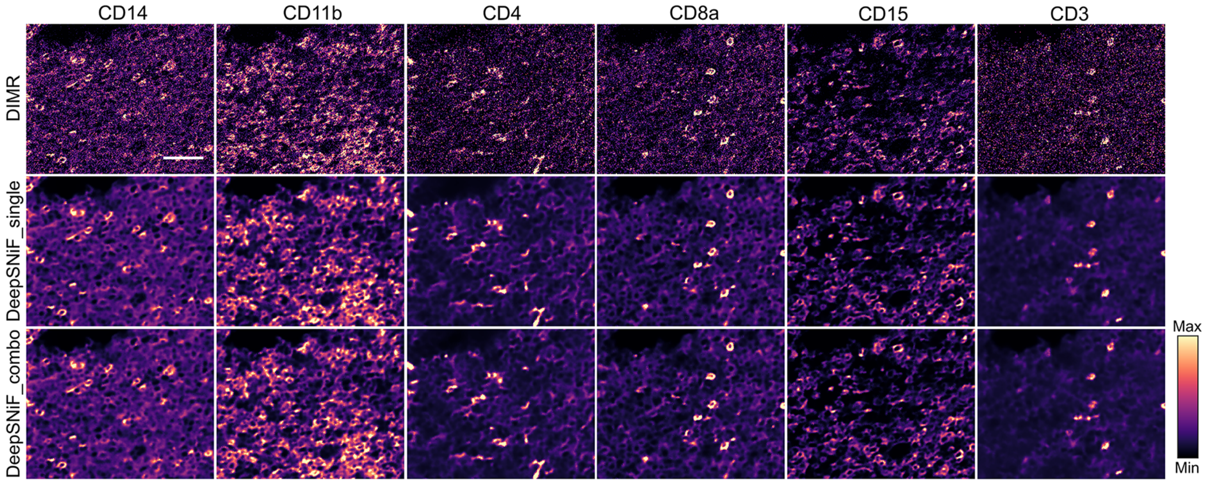


**Supplementary Figure 46.** DeepSNiF works on multiple markers training. The DIMR-processed IMC images were trained by DeepSNiF with single marker in each network (DeepSNiF_single) and all the markers in a single network (DeepSNiF_combo), respectively. The denoising results indicate both approaches enables IMC image quality improvement. Scale bar: 48 $\mu$m.

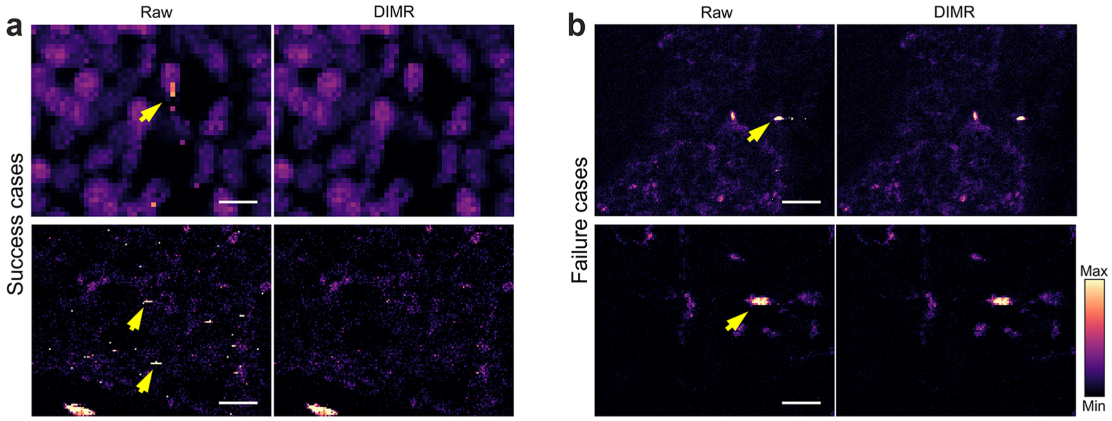


**Supplementary Figure 47.** The limitation of the DIMR algorithm. (a) Success cases of DIMR on challenging hot pixels. (b) Failure cases of DIMR on hot clusters. DIMR is able to remove line-style consecutive hot pixels while fails on hot clusters. Scale bar: (a) Top: 8 $\mu$m, bottom: 24 $\mu$m. (b) Top: 40 $\mu$m, bottom: 20 $\mu$m.

# Other Supplementary Tables

Supplementary Table 1 and details can be found on page 20.

**Supplementary Table 2.** List of cell markers used for Collagen III-labeled tissues in Fig. 1d and Fig. 2a

| Isotope | Metal | Epitope | Clone | Source | Catalog # | Dilution |
|---|---|---|---|---|---|---|
| 141 | Pr | CD235 * | HIR2 | Fluidigm | 3141001B | 1:200 |
| 142 | Nd | MPO * | polyclonal | Dako | A0398 | 1:800 |
| 144 | Nd | CD14 * | EPR3653 | Fluidigm | 3144025D | 1:800 |
| 145 | Nd | CD117 * | YR145 | Abcam | ab216450 | 1:100 |
| 147 | Sm | CD163 * | EDHu-1 | Fluidigm | 3147021D | 1:100 |
| 148 | Nd | CD71 * | MRQ-48 | eBiosciences | 14-0718-93 | 1:200 |
| 149 | Sm | CD11b * | EPR1344 | Fluidigm | 3149028D | 1:150 |
| 151 | Eu | CD31 * | EPR3094 | Fluidigm | 3151025D | 1:100 |
| 152 | Sm | CD34 * | QBend/10 | ThermoFisher | MA1-10202 | 1:400 |
| 153 | Eu | pSTAT5 | 47 | BD | custom quote | 1:100 |
| 154 | Sm | TNFa | TNF706 + P/T2 | Abcam | ab212899 | 1:150 |
| 155 | Gd | IL8 | 807 | Abcam | custom quote | 1:200 |
| 156 | Gd | CD4 * | EPR6855 | Fluidigm | 3156033D | 1:500 |
| 157 | Gd | IL6 | 1936 | R&D | MAB2061 | 1:100 |
| 158 | Gd | pSTAT3 | 4/P-STAT3 | Fluidigm | 3158030D | 1:100 |
| 159 | Tb | CD90 | 5E10 | Fluidigm | 3159007B | 1:100 |
| 160 | Gd | CD61 * | 2f2 | Sigma | custom quote | 1:400 |
| 161 | Dy | CD20 * | H1 | Fluidigm | 3161029D | 1:400 |
| 162 | Dy | CD8a * | C8/144B | Fluidigm | 3162034D | 1:300 |
| 163 | Dy | TGFb | TB21 | Invitrogen | MA1-21595 | 1:800 |
| 164 | Dy | CD15 * | W6D3 | Fluidigm | 3164001B | 1:150 |
| 165 | Ho | pCREB | 87G3 | Fluidigm | 3165009A | 1:400 |
| 166 | Er | p65 pS529 | K10x | Fluidigm | 3166006A | 1:200 |
| 167 | Er | RELA | 2A12A7 | ThermoFisher | 33-9900 | 1:100 |
| 168 | Er | Ki-67 * | B56 | Fluidigm | 3168022D | 1:200 |
| 169 | Tm | pIKK$\alpha/\beta$ | 16A6 | CST | 2697BF | 1:200 |
| 170 | Er | CD3 * | polyclonal | Fluidigm | 3170019D | 1:200 |

**Table 2 continued from previous page**

| | | | | | | |
|---|---|---|---|---|---|---|
| 172 | Yb | Cleaved casp 3 | 5A1E | Fluidigm | 3172027D | 1:300 |
| 174 | Yb | pERK1/2 | D13.14.4E | Fluidigm | 3171021D | 1:100 |
| 175 | Lu | pS6 | N7-548 | Fluidigm | 3175009A | 1:400 |
| 176 | Yb | Histone H3 * | D1H2 | Fluidigm | 3176023D | 1:2000 |
| 209 | Bi | Collagen III * | polyclonal | Southern Biotech | 1330-01 | 1:100 |
| 191/193 | Ir | intercalator * | | | | 1:300 |

* Denotes markers with validated staining patterns on this tissue

**Supplementary Table 3.** List of cell markers for the tissue staining in Fig. 1g

| Isotope | Metal | Epitope | Clone | Source | Catalog # | Dilution |
|---|---|---|---|---|---|---|
| 142 | Nd | MPO * | polyclonal | Dako | A0398 | 1:400 |
| 145 | Nd | CD117 * | YR145 | Abcam | ab216450 | 1:50 |
| 150 | Nd | CXCL12 | 79018 | Novus | MAB350 | 1:75 |
| 153 | Eu | IFNg | IFNG/466 | Novus | NBP2-54394 | 1:25 |
| 154 | Sm | TNFa | TNF706 + P/T2 | Abcam | ab212899 | 1:75 |
| 155 | Gd | IL8 | 807 | Abcam | custom quote | 1:25 |
| 157 | Gd | IL6 | 1936 | R&D | MAB2061 | 1:25 |
| 158 | Gd | pSmad4 | polyclonal | ThermoFisher | PA5-12695 | 1:150 |
| 159 | Tb | CD169 * | SP213 | Abcam | ab245735 | 1:100 |
| 163 | Dy | CD271 | EP1039Y | Abcam | ab256584 | 1:50 |
| 173 | Yb | Collagen III * | polyclonal | Southern Biotech | 1330-01 | 1:400 |
| 209 | Bi | Collagen III * | polyclonal | Southern Biotech | 1330-01 | 1:200 |
| 191/193 | Ir | intercalator | | | | 1:200 |

* Denotes markers with validated staining patterns on this tissue

**Supplementary Table 4.** List of cell markers for the tissue staining in Supplementary Fig. 20

| Isotope | Metal | Epitope | Clone | Source | Catalog # | Dilution |
|---|---|---|---|---|---|---|
| 142 | Nd | MPO * | polyclonal | Dako | A0398 | 1:400 |
| 143 | Nd | TP53 * | DO-7 | Fluidigm | 3143026D | 1:50 |
| 146 | Nd | TP53 * | DO-7 | Biolegend | 645802 | 1:50 |
| 150 | Nd | CXCL12 | 79018 | Novus | MAB350 | 1:50 |
| 158 | Gd | CD56 * | MRQ-42 | CellMarque | custom quote | 1:100 |
| 163 | Dy | CD271 | EP1039Y | Abcam | ab256584 | 1:50 |
| 167 | Er | GranzymeB | EPR20129-217 | Fluidigm | 3167021D | 1:600 |
| 173 | Yb | CD56 * | MRQ-42 | CellMarque | custom quote | 1:100 |
| 191/193 | Ir | intercalator | | | | 1:300 |

* Denotes markers with validated staining patterns on this tissue

**Supplementary Table 5.** List of cell markers used for other IMC images from the human bone marrow IMC dataset

| Isotope | Metal | Epitope | Clone | Source | Catalog # | Dilution |
|---|---|---|---|---|---|---|
| 89 | Yb | Alpha-SMA * | 1A4 | Bio-Rad | MCA5781GA | 1:100 |
| 115 | In | perilipin * | D1D8 | CST | 9349 custom | 1:50 |
| 139 | La | VCAM1 | EPR5047 | Abcam | ab215380 | 1:50 |
| 141 | Pr | CD38 * | EPR4106 | Fluidigm | 3141018D | 1:50 |
| 142 | Nd | MPO * | polyclonal | Dako | A0398 | 1:400 |
| 143 | Nd | vimentin * | RV202 | Fluidigm | 3143029D | 1:200 |
| 144 | Nd | CD14 * | EPR3653 | Fluidigm | 3144025D | 1:400 |
| 145 | Nd | CD117 * | YR145 | Abcam | ab216450 | 1:50 |
| 146 | Nd | CD16 | EPR16784 | Fluidigm | 3146020D | 1:150 |
| 147 | Sm | CD163 * | EDHu-1 | Fluidigm | 3147021D | 1:100 |
| 148 | Nd | CD71 * | MRQ-48 | eBiosciences | 14-0718-93 | 1:50 |
| 149 | Sm | CD11b * | EPR1344 | Fluidigm | 3149028D | 1:150 |
| 150 | Nd | CXCL12 | 79018 | Novus | MAB350 | 1:25 |
| 151 | Eu | CD31 * | EPR3094 | Fluidigm | 3151025D | 1:50 |
| 152 | Sm | CD34 * | QBend/10 | ThermoFisher | MA1-10202 | 1:50 |
| 153 | Eu | IFNg | IFNG/466 | Novus | NBP2-54394 | 1:25 |
| 154 | Sm | TNFa | TNF706 + P/T2 | Abcam | ab212899 | 1:100 |
| 156 | Gd | CD4 * | EPR6855 | Fluidigm | 3156033D | 1:200 |
| 157 | Gd | IL6 | 1936 | R&D | MAB2061 | 1:25 |
| 158 | Gd | pSmad4 | polyclonal | ThermoFisher | PA5-12695 | 1:150 |
| 159 | Tb | CD169 * | SP213 | Abcam | ab245735 | 1:100 |
| 160 | Gd | CD61 * | 2f2 | Sigma | custom quote | 1:100 |
| 161 | Dy | CD20 * | H1 | Fluidigm | 3161029D | 1:400 |
| 162 | Dy | CD8a * | C8/144B | Fluidigm | 3162034D | 1:300 |
| 163 | Dy | CD271 | EP1039Y | Abcam | ab256584 | 1:50 |
| 164 | Dy | CD15 * | W6D3 | Fluidigm | 3164001B | 1:150 |
| 165 | Ho | pH2AX | N1-431 | Fluidigm | 3165036D | 1:150 |
| 166 | Er | p65 pS529 | K10x | Fluidigm | 3166006A | 1:25 |
| 167 | Er | SCF | polyclonal | ThermoFisher | PA5-20746 | 1:25 |

**Table 5 continued from previous page**

| | | | | | | |
|---|---|---|---|---|---|---|
| 168 | Er | Ki-67 * | B56 | Fluidigm | 3168022D | 1:100 |
| 169 | Tm | Collagen I * | polyclonal | Fluidigm | 3169023D | 1:2000 |
| 170 | Er | CD3 * | polyclonal | Fluidigm | 3170019D | 1:100 |
| 171 | Yb | pERK1/2 | D13.14.4E | Fluidigm | 3171021D | 1:50 |
| 172 | Yb | Cleaved casp 3 | 5A1E | Fluidigm | 3172027D | 1:25 |
| 173 | Yb | CD45RO * | UCHL1 | Fluidigm | 3173016D | 1:500 |
| 174 | Yb | HLA-DR * | YE2/36HLK | Fluidigm | 3174023D | 1:100 |
| 175 | Lu | CD235a * | HIR2 | Fluidigm | 3175029D | 1:200 |
| 176 | Yb | Histone H3 * | D1H2 | Fluidigm | 3176023D | 1:2000 |
| 209 | Bi | Collagen III * | polyclonal | Southern Biotech | 1330-01 | 1:75 |
| 191/193 | Ir | intercalator | | | | 1:200 |

Note: The tissues with headers of K, L do not have CXCL12.

* Denotes markers with validated staining patterns on this tissue.

**Supplementary Table 6.** Training details for the simulation datasets

| #Patches | Normalzed percentile | training time |
|---|---|---|
| 12000 | 99.999 | 89 min |

**Supplementary Table 7.** Training details for the Collagen III-labeled images in Fig. 1d, g and Fig.2a

| Marker | #Patches | Normalzed percentile | Background thresh $\rho$ | training time |
|---|---|---|---|---|
| Collagen III | 1992 | 99.9 | 0.55 | 17 min |

**Supplementary Table 8.** Training details for the other markers-labeled images from the human bone marrow IMC dataset

| Marker | #Patches | Normalzed percentile | Background thresh $\rho$ | training time |
|---|---|---|---|---|
| CD38 | 21768 | 99.999 | 0.9 | 160 min |
| MPO | 20800 | 99.999 | 0.8 | 153 min |
| CD14 | 21784 | 99.999 | 0.9 | 160 min |
| CD71 | 14960 | 99.999 | 0.9 | 110 min |
| CD11b | 20096 | 99.999 | 0.9 | 147 min |
| CD31 | 9040 | 99.99 | 0.75 | 67 min |
| CD34 | 15208 | 99.9 | 0.85 | 114 min |
| CD4 | 20832 | 99.9 | 0.9 | 154 min |
| CD169 | 20800 | 99.999 | 0.9 | 153 min |
| CD61 | 3360 | 99.9 | 0.75 | 27 min |
| CD20 | 12304 | 99.9 | 0.95 | 90 min |
| CD8a | 19360 | 99.999 | 0.9 | 144 min |
| CD15 | 17456 | 99.99 | 0.9 | 127 min |
| Ki-67 | 18032 | 99.999 | 0.9 | 134 min |
| CD3 | 16728 | 99.99 | 0.9 | 124 min |
| CD45RO | 11672 | 99.99 | 0.75 | 87 min |
| CD235a | 21144 | 99.999 | 0.7 | 154 min |
| Histone H3 | 14952 | 99.999 | 0.5 | 108 min |
| DNA2 | 22136 | 99.999 | 0.4 | 161 min |
| Combinations of CD4, CD8a, CD3, CD14, CD11b, CD71 and CD15 | 131216 | 99.9 | | 15.8 h |

**Supplementary Table 9.** Training details for the images from human breast cancer IMC dataset

| Marker | #Patches | Normalzed percentile | Background thresh $\rho$ | training time |
|---|---|---|---|---|
| CD3 | 12592 | 99.9 | 0.95 | 94 min |
| CD20 | 17592 | 99.9 | 0.95 | 133 min |
| CD45 | 9368 | 99.999 | 0.9 | 72 min |
| CD68 | 13024 | 99.999 | 0.85 | 97 min |
| c-Myc | 14056 | 99.999 | 0.85 | 104 min |
| EGFR | 17624 | 99.999 | 0.85 | 135 min |
| EpCAM | 15064 | 99.999 | 0.8 | 112 min |
| Ki-67 | 10824 | 99.999 | 0.9 | 80 min |
| Rabbit IgG H L | 10928 | 99.999 | 0.9 | 84 min |
| Slug | 11856 | 99.999 | 0.9 | 90 min |
| Twist | 14496 | 99.999 | 0.9 | 107 min |
| vWF | 17928 | 99.999 | 0.95 | 136 min |

**Supplementary Table 10.** Training details for the images from the human pancreatic cancer IMC dataset

| Marker | #Patches | Normalzed percentile | Background thresh $\rho$ | training time |
|---|---|---|---|---|
| CD3 | 16304 | 99.99 | 0.75 | 120 min |
| CD4 | 14344 | 99.999 | 0.7 | 107 min |
| CD8 | 9792 | 99.9 | 0.77 | 74 min |
| CD11b | 21120 | 99.99 | 0.3 | 154 min |
| CD14 | 10896 | 99.99 | 0.5 | 82 min |
| CD31 | 17088 | 99.99 | 0.75 | 126 min |
| CD44 | 17392 | 99.99 | 0.5 | 127 min |
| CD45 | 21984 | 99.99 | 0.7 | 161 min |
| CD45RO | 18056 | 99.99 | 0.6 | 134 min |
| CD56 | 11168 | 99.99 | 0.5 | 84 min |
| Foxp3 | 9240 | 99.99 | 0.4 | 70 min |
| pS6 | 19440 | 99.99 | 0.2 | 144 min |

**Supplementary Table 11.** Training details for the images from the IMC dataset

| Marker | #Patches | Normalzed percentile | Background thresh $\rho$ | training time |
|---|---|---|---|---|
| CD3 | 5096 | 99.999 | 0.95 | 43 min |
| CD4 | 4120 | 99.999 | 0.95 | 34 min |
| CD8 | 7472 | 99.999 | 0.98 | 57 min |
| CD11b | 11400 | 99.999 | 0.95 | 87 min |
| CD11c | 6720 | 99.999 | 0.96 | 50 min |
| CD14 | 9768 | 99.999 | 0.95 | 74 min |
| CD20 | 13184 | 99.999 | 0.95 | 97 min |
| CD31 | 7272 | 99.999 | 0.99 | 57 min |
| CD45 | 13792 | 99.999 | 0.95 | 104 min |
| CD68 | 8368 | 99.999 | 0.98 | 64 min |
| CD206 | 10672 | 99.999 | 0.98 | 80 min |
| HLA-DR | 13296 | 99.999 | 0.95 | 100 min |

**Supplementary Table 12.** The estimated thresholds for positive markers extracted from DeepSNiF-based segmented masks (round to 4 decimal places)

| Marker | Value | Marker | Value |
|---|---|---|---|
| CD38 | 1.3981 | CD169 | 2.5625 |
| MPO | 5.2270 | CD20 | 0.6300 |
| CD14 | 2.3947 | CD8a | 3.3559 |
| CD71 | 0.9478 | CD15 | 3.0374 |
| CD11b | 2.2740 | CD3 | 0.9180 |
| CD4 | 0.7830 | CD235a | 35.7895 |

**Supplementary Table 13.** The estimated thresholds for positive markers extracted from DIMR-based segmented masks (round to 4 decimal places)

| Marker | Value | Marker | Value |
|---|---|---|---|
| CD38 | 1.3612 | CD169 | 2.5800 |
| MPO | 5.2992 | CD20 | 0.6061 |
| CD14 | 2.4294 | CD8a | 3.3333 |
| CD71 | 0.9567 | CD15 | 3.0613 |
| CD11b | 2.2878 | CD3 | 0.9167 |
| CD4 | 0.8081 | CD235a | 35.1900 |

# References


[1] Chevrier, S. et al. Compensation of signal spillover in suspension and imaging mass cytometry. *Cell Syst.* **6**, 612–620 (2018).

[2] Lu, P. et al. Blind image restoration enhances digital autoradiographic imaging of radiopharmaceutical tissue distribution. *J. Nucl. Med.* **63**, 591–597 (2022).

[3] Anscombe, F. J. The transformation of poisson, binomial and negative-binomial data. *Biometrika* **35**, 246–254 (1948).

[4] Bar-Lev, S. K. & Enis, P. On the classical choice of variance stabilizing transformations and an application for a poisson variate. *Biometrika* **75**, 803–804 (1988).

[5] Russell, M. F. T. B. C. & Freeman, W. T. Exploiting the sparse derivative prior for super-resolution and image demosaicing. *SCTV*, 1–28 (2003).

[6] Rudin, W. PRINCIPLES OF MATHEMATICAL ANALYSIS (McGraw-hill, New York, 1964).

[7] Silverman, B. W. DENSITY ESTIMATION FOR STATISTICS AND DATA ANALYSIS (Routledge, New York, 2018).

[8] Makitalo, M. & Foi, A. Optimal inversion of the Anscombe transformation in low-count Poisson image denoising. *IEEE Trans. Image Process.* **20**, 99–109 (2010).

[9] Finesso, L. & Spreij, P. Nonnegative matrix factorization and I-divergence alternating minimization. *Linear Algebra Its Appl.* **416**, 270–287 (2006).

[10] Krull, A., Buchholz, T. O. & Jug, F. Noise2void-learning denoising from single noisy images. *CVPR*, 2129–2137 (2019).

[11] Batson, J. & Royer, L. Noise2self: Blind denoising by self-supervision. *PMLR*, 524–533 (2019).

[12] Huang, X. et al. Fast, long-term, super-resolution imaging with hessian structured illumination microscopy. *Nat. Biotechnol.* **36**, 451–459 (2018).

[13] Zhao, W. et al. Sparse deconvolution improves the resolution of live-cell super-resolution fluorescence microscopy. *Nat. Biotechnol.* **40**, 606–617 (2022).

[14] Zanotelli, V. & Bodenmiller, B. IMC segmentation pipeline: a pixel classification based multiplexed image segmentation pipeline. Zenodo, https://doi.org/10.5281/zenodo.3841960 (2017).

[15] Rendeiro, A. F. et al. The spatial landscape of lung pathology during COVID-19 progression. *Nature* **593**, 564–569 (2021).

[16] Wu, M. et al. Single-cell analysis of the human pancreas in type 2 diabetes using multi-spectral imaging mass cytometry. *Cell Rep.* **37**, 109919 (2021).

[17] Wang, Y. J. et al. Multiplexed in situ imaging mass cytometry analysis of the human endocrine pancreas and immune system in type 1 diabetes. *Cell Metab.* **29**, 769–783 (2019).

[18] Xu, B., Wang, N., Chen, T. & Li, M. Empirical evaluation of rectified activations in convolutional network. Preprint at *arXiv* https://arxiv.org/abs/1505.00853 (2015).

[19] Weigert, M. et al. Content-aware image restoration: pushing the limits of fluorescence microscopy. *Nat. Methods* **15**, 1090–1097 (2018).

[20] Buades, A., Coll, B. & Morel, J. M. A non-local algorithm for image denoising. *CVPR* **2**, 60–65 (2005).

[21] Dabov, K., Foi, A., Katkovnik, V. & Egiazarian, K. Image denoising by sparse 3-D transform-domain collaborative filtering. *IEEE Trans. Image Process.* **16**, 2080–2095 (2007).

[22] Rashid, R. et al. Highly multiplexed immunofluorescence images and single-cell data of immune markers in tonsil and lung cancer. *Sci. Data* **6**, 1–10 (2019).

[23] Wang, Z., Bovik, A. C., Sheikh, H. R. & Simoncelli, E. P. Image quality assessment: from error visibility to structural similarity. *IEEE Trans. Image Process.* **13**, 600–612 (2004).

[24] Garnett, R., Huegerich, T., Chui, C. & He, W. A universal noise removal algorithm with an impulse detector. *IEEE Trans. Image Process.* **11**, 1747–1754 (2005).

[25] Dong, Y., Chan, R. H. & Xu, S. A detection statistic for random-valued impulse noise. *IEEE Trans. Image Process.* **16**, 1112–1120 (2007).
