# Supplementary material for: IMC-Denoise: a content aware denoising pipeline to enhance Imaging Mass Cytometry
Source: Nat Commun. 2023 Mar 23;14:1601. doi: 10.1038/s41467-023-37123-6 (PMC10036333; doi:10.1038/s41467-023-37123-6)
Supplement: Supplementary file 3 — Reporting Summary [file 41467_2023_37123_MOESM3_ESM.pdf]

nature portfolio

Corresponding author(s): Daniel Thorek

Last updated by author(s): Feb 2, 2023

Reporting Summary

Nature Portfolio wishes to improve the reproducibility of the work that we publish. This form provides structure for consistency and transparency in reporting. For further information on Nature Portfolio policies, see our Editorial Policies and the Editorial Policy Checklist.

Statistics

For all statistical analyses, confirm that the following items are present in the figure legend, table legend, main text, or Methods section.

n/a	Confirmed	
[ ]	[x]	The exact sample size (*n*) for each experimental group/condition, given as a discrete number and unit of measurement
[ ]	[x]	A statement on whether measurements were taken from distinct samples or whether the same sample was measured repeatedly
[ ]	[x]	The statistical test(s) used AND whether they are one- or two-sided<br>*Only common tests should be described solely by name; describe more complex techniques in the Methods section.*
[ ]	[x]	A description of all covariates tested
[ ]	[x]	A description of any assumptions or corrections, such as tests of normality and adjustment for multiple comparisons
[ ]	[x]	A full description of the statistical parameters including central tendency (e.g. means) or other basic estimates (e.g. regression coefficient) AND variation (e.g. standard deviation) or associated estimates of uncertainty (e.g. confidence intervals)
[ ]	[x]	For null hypothesis testing, the test statistic (e.g. *F*, *t*, *r*) with confidence intervals, effect sizes, degrees of freedom and *P* value noted<br>*Give P values as exact values whenever suitable.*
[x]	[ ]	For Bayesian analysis, information on the choice of priors and Markov chain Monte Carlo settings
[x]	[ ]	For hierarchical and complex designs, identification of the appropriate level for tests and full reporting of outcomes
[ ]	[x]	Estimates of effect sizes (e.g. Cohen's *d*, Pearson's *r*), indicating how they were calculated

Our web collection on statistics for biologists contains articles on many of the points above.

Software and code

Policy information about availability of computer code

Data collection	Fluidigm CyTOF Software versino 7.08493 for IMC data, and LAS X software version 3.7.3.23245 of IF data acquisition.
Data analysis	Data simulation was performed using custom Matlab (R2021a, MathWorks) scripts. All the accuracy metrics, Gaussian and NLM filtering were computed using Matlab (R2021a, MathWorks) built-in functions. The BM3D algorithm software package (version 3.0.9) was downloaded from https://webpages.tuni.fi/foi/GCF-BM3D/index.html#ref_software. The exact unbiased inverse Anscombe transformation software package (version 1.0.0) was downloaded from https://webpages.tuni.fi/foi/invansc/.<br>Deep learning models reported in this work were implemented with standard libraries of Python (3.6.13), TensorFlow (2.2.0, Google), and Keras (2.3.1, Google). The IMC-Denoise package is available through the github reposity at https://github.com/PENGLU-WashU/IMC_Denoise. All necessary datasets and pre-trained networks are available through the zenodo reposities at https://doi.org/10.5281/zenodo.6533905 and https://doi.org/10.5281/zenodo.6516116.<br>For segmentation and single-cell feature extraction CellProfiler version 3.1.8, Ilastik version 1.3.2post1 and histoCAT version 1.76 were used. For semi-automated background noise removal, Ilastik version 1.3.2 was used. The MAUI software was downloaded from https://github.com/angelolab/MAUI . For positive cell identification, Scikit-learn version (1.0.2) was used. The Jaccard graph construction and Leiden algorithm were implemented by the software packages from https://github.com/jacoblevine/PhenoGraph and https://github.com/vtraag/leidenalg, respectively. The fast interpolation-based t-SNE algorithm was implemented by the software package in https://github.com/KlugerLab/FItSNE. Prism 9 was used for statistical analysis.

For manuscripts utilizing custom algorithms or software that are central to the research but not yet described in published literature, software must be made available to editors and reviewers. We strongly encourage code deposition in a community repository (e.g. GitHub). See the Nature Portfolio guidelines for submitting code & software for further information.

Data

Policy information about availability of data

All manuscripts must include a data availability statement. This statement should provide the following information, where applicable:

- Accession codes, unique identifiers, or web links for publicly available datasets
- A description of any restrictions on data availability
- For clinical datasets or third party data, please ensure that the statement adheres to our policy

The human bone marrow IMC data and simulated data are available from Zenodo https://doi.org/10.5281/zenodo.6533905. The human pancreatic cancer IMC dataset [10], the human breast cancer IMC dataset [12], and the MIBI data set [19] can be downloaded through the links provided by the authors in their papers, respectively. All the other data supporting the results in this paper can be accessed from https://doi.org/10.5281/zenodo.7336448. A previous link at https://doi.org/10.5281/zenodo.6516116 (shared in a preliminary non peer reviewed version of the paper (biorxiv)) should also take reviewers and readers to an updated repository.

Human research participants

Policy information about studies involving human research participants and Sex and Gender in Research.

Reporting on sex and gender	Sex and gender information has not been used in this secondary analysis of archival data.
Population characteristics	Samples were selected from a tissue repository. Patients who underwent bone marrow biopsy with longitudinal timepoints throughout treatment were selected. No selection criteria were applied for particular patient characteristics for the current report. Patient samples were obtained from four patients with untreated myelodysplastic syndromes (age range 50-63 years old, 3 male, 1 female) and subsequent timepoint samples from these patients were obtained during progression to acute myeloid leukemia during treatment with hypomethylating agents (3 patients) or supportive care (1 patient). Normal tissue samples were obtained from anonymous surgical pathology specimens.
Recruitment	This was a secondary analysis of archival tissue biospecimens collected from patients, under IRB 201912110.
Ethics oversight	Washington University in St. Louis School of Medicine Institutional Review Board.

Note that full information on the approval of the study protocol must also be provided in the manuscript.

Field-specific reporting

Please select the one below that is the best fit for your research. If you are not sure, read the appropriate sections before making your selection.

[x] Life sciences [ ] Behavioural & social sciences [ ] Ecological, evolutionary & environmental sciences

For a reference copy of the document with all sections, see nature.com/documents/nr-reporting-summary-flat.pdf

Life sciences study design

All studies must disclose on these points even when the disclosure is negative.

Sample size	No sample size calculations were conducted prior to experiments or analysis, except that tissues were used for analysis which enabled >10000 cells to be identified for image analysis (including downstream single cell analyses). Sample number are sufficiently purposive to provide representative pathological data. Sample size was chosen and deemed sufficient as required for training the algorithm, and this was informed by the larger datasets that were available for IMC (used and cited in this work).
Data exclusions	Samples (tissue sections) were excluded from the study if they were poorly stained, or appeared to have significant artifacts.
Replication	Replicates of IMC acquisition from the same tissue area is not possible as the method is destructive. We have developed simulations, analyzed available data sets, and generated new ones and repeatedly run our methods on these datasets, more than 3 times on the bone marrow IMC dataset. We consistently observe data features which indicate the robustness of the approach and applicability, and the IMC-Denoise pipeline was successful as long as sufficient memory was available.
Randomization	Randomization of samples was not necessary in this work. We have randomized some of the analysis, please see the "leave-one-out" evaluation strategy in the Cell Type Annotation section of the results for a full description. Briefly, we randomized the inclusion of the DeepSNF algorithm to restore image data quality and compare this to the raw case in order to determine the impact for specific channel's denoising and restoration
Blinding	Blinding in acquisition is not possible because the samples have to be manually imaged; blinding is also not necessary as images need to have identity to act as training or testing data. Blinding was not necessary in our study for comparison of methods of image analysis.

Reporting for specific materials, systems and methods

We require information from authors about some types of materials, experimental systems and methods used in many studies. Here, indicate whether each material, system or method listed is relevant to your study. If you are not sure if a list item applies to your research, read the appropriate section before selecting a response.

Materials & experimental systems

n/a	Involved in the study
☐	☒ Antibodies
☒	☐ Eukaryotic cell lines
☒	☐ Palaeontology and archaeology
☒	☐ Animals and other organisms
☒	☐ Clinical data
☒	☐ Dual use research of concern

Methods

n/a	Involved in the study
☒	☐ ChIP-seq
☒	☐ Flow cytometry
☒	☐ MRI-based neuroimaging

Antibodies

Antibodies used

Please see the attached supplemental information file with extensive documentation of antibody sourcing, dilution, conjugation and other details.

Validation

Antibody staining patterns were compared with marker patterns in the literature, atlases, and established clinical staining patterns in this tissue when possible. Cell lineage markers were reviewed across each panel in the normal specimens for consistent co-expression and mutually exclusive expression patterns of known cell populations for validation, and are denoted with asterisk in the Supplementary Tables 2—5. Only fully validated markers are included in further analysis. A list of the antibodies and their validation references are included below. Each antibody epitope is followed by its clone, source, catalog number, RRID, and references.

Alpha-SMA 1A4 Bio-Rad MCA5781GA RRID: AB_262054, PMID: 34977680, Invitrogen advanced verification https://www.thermofisher.com/antibody/product/Alpha-Smooth-Muscle-Actin-Antibody-clone-1A4-Monoclonal/14-9760-82

CD117 YR145 Abcam ab216450 AB_2868615 knockout validated https://www.abcam.com/c-kit-antibody-yr145-ab32363.html

CD11b EPR1344 Fluidigm 3149028D AB_2891189 PMID:28525591, PMID:29275866, PMID:29706577, PMID:29937354, PMID:29958804, PMID:29964156, PMID:29993362, PMID:30057172, PMID:30225910, PMID:30332632, PMID:30827729, PMID:31061090, PMID:31327655, PMID:31373638, PMID:31563141, PMID:32390866

CD14 EPR3653 Fluidigm 3144025D RRID: AB_2889158, PMID: 34977680, IVD: https://www.cellmarque.com/antibodies/CM/2066/CD14_EPR3653

CD15 W6D3 Fluidigm 3164001B AB_2810970 PMID: 32573435

CD163 EDHu-1 Fluidigm 3147021D AB_2892115 PMID: 34873687, PMID: 17353345

CD169 SP213 Abcam ab245735 PMID: 25891017

CD20 H1 Fluidigm 3161029D AB_2811016 PMID: 34977680, PMID: 29605184

CD235a HIR2 Fluidigm 3141001B, 3175029D AB_2651154 also known as GA-R2, IVD: https://www.cellmarque.com/antibodies/CM/91/Glycophorin-A_GA-R2

CD3 polyclonal Fluidigm 3170019D AB_2811048 PMID: 34977680

CD31 EPR3094 Fluidigm 3151025D AB_2890140 PMID: 34157151

CD34 QBend/10 ThermoFisher MA1-10202 AB_11156010 PMID:10627452, IVD: https://www.cellmarque.com/antibodies/CM/43/CD34_QBEnd-10

CD38 EPR4106 Fluidigm 3141018D RRID:AB_2864383, PMID: 30713109

CD4 EPR6855 Fluidigm 3156033D AB_2811051 PMID: 34977680, PMID:31730855

CD45RO UCHL1 Fluidigm 3173016D AB_2811052 PMID: 34977680, IVD: https://www.cellmarque.com/antibodies/CM/48/CD45RO_UCHL-1

CD56 MRQ-42 CellMarque custom carrier free RRID:AB_2861293, IVD: https://www.cellmarque.com/antibodies/CM/2092/CD56_MRQ-42

CD61 2f2 Sigma custom carrier free RRID:AB_1158112, IVD: https://www.cellmarque.com/antibodies/CM/51/CD61_2f2

CD71 MRQ-48 eBiosciences 14-0718-93 AB_2637425 IVD: https://www.cellmarque.com/antibodies/CM/2116/CD71_MRQ-48

CD8a C8/144B Fluidigm 3162034D AB_2811053 PMID:20591136, PMID:21818290, PMID:29993362, PMID:9763512, IVD: https://www.cellmarque.com/antibodies/CM/35/CD8_C8-144B

Collagen I polyclonal Fluidigm 3169023D AB_2810857 PMID:31368890

Collagen III polyclonal Southern Biotech 1330-01 AB_2794734 PMID: 8055531, PMID:31722201

Histone H3 D1H2 Fluidigm 3176023D AB_2811058 PMID: 34977680

HLA-DR YE2/36HLK Fluidigm 3174023D AB_2811059 PMID: 27285579

Ki-67 B56 Fluidigm 3168022D AB_2811061 PMID: 34977680, knockout validated https://www.abcam.com/ki67-antibody-b56-ab279653.html

MPO polyclonal Dako A0398 AB_2335676 PMID:25763638, PMID:27355490, PMID:30107174, PMID:30713109, PMID:30979687, PMID:31526760, IVD https://www.agilent.com/store/productDetail.jsp?catalogId=A039829-2

perilipin D1D8 CST 9349 AB_10829911 PMID:28709001, PMID:28860152, PMID:30016424, PMID:30773468, PMID:31809738, PMID:31940497

TP53 DO-7 Biolegend 645802 AB_2206460 PMID: 30792212

vimentin RV202 Fluidigm 3143029D AB_2811069 PMID:25520056, PMID:27432616, knockout validated https://www.abcam.com/

vimentin-antibody-rv202-cytoskeleton-marker-ab8978.html
IVD: In vitro diagnostic use.
